# Feature engineering of environmental covariates improves plant genomic-enabled prediction

**DOI:** 10.3389/fpls.2024.1349569

**Published:** 2024-05-15

**Authors:** Osval A. Montesinos-López, Leonardo Crespo-Herrera, Carolina Saint Pierre, Bernabe Cano-Paez, Gloria Isabel Huerta-Prado, Brandon Alejandro Mosqueda-González, Sofia Ramos-Pulido, Guillermo Gerard, Khalid Alnowibet, Roberto Fritsche-Neto, Abelardo Montesinos-López, José Crossa

**Affiliations:** ^1^ Facultad de Telemática, Universidad de Colima, Colima, Mexico; ^2^ International Maize and Wheat Improvement Center (CIMMYT), Texcoco, Edo. de Mexico, Mexico; ^3^ Facultad de Ciencias, Universidad Nacioanl Autónoma de México (UNAM), México City, Mexico; ^4^ Independent consultant, Zinacatepec, Puebla, Mexico; ^5^ Centro de Investigación en Computación (CIC), Instituto Politécnico Nacional (IPN), México City, Mexico; ^6^ Centro Universitario de Ciencias Exactas e Ingenierías (CUCEI), Universidad de Guadalajara, Guadalajara, Jalisco, Mexico; ^7^ Department of Statistics and Operations Research, King Saud University, Riyah, Saudi Arabia; ^8^ Louisiana State University, Baton Rouge, LA, United States; ^9^ Distinguished Scientist Fellowship Program, King Saud University, Riyah, Saudi Arabia; ^10^ Instituto de Socieconomia, Estadistica e Informatica, Colegio de Postgraduados, Montecillos, Edo. de México, Texcoco, Mexico

**Keywords:** genomic selection, plant breeding, environmental covariates, feature engineering, feature selection

## Abstract

**Introduction:**

Because Genomic selection (GS) is a predictive methodology, it needs to guarantee high-prediction accuracies for practical implementations. However, since many factors affect the prediction performance of this methodology, its practical implementation still needs to be improved in many breeding programs. For this reason, many strategies have been explored to improve the prediction performance of this methodology.

**Methods:**

When environmental covariates are incorporated as inputs in the genomic prediction models, this information only sometimes helps increase prediction performance. For this reason, this investigation explores the use of feature engineering on the environmental covariates to enhance the prediction performance of genomic prediction models.

**Results and discussion:**

We found that across data sets, feature engineering helps reduce prediction error regarding only the inclusion of the environmental covariates without feature engineering by 761.625% across predictors. These results are very promising regarding the potential of feature engineering to enhance prediction accuracy. However, since a significant gain in prediction accuracy was observed in only some data sets, further research is required to guarantee a robust feature engineering strategy to incorporate the environmental covariates.

## Introduction

The global population’s rapid growth is increasing food demand, but climate change impacts crop productivity. Plant breeding is essential for high-yield, quality cultivars. Wheat production soared from 200 million tons in 1961 to 775 million tons in 2023 without expanding cultivation, thanks to improved cultivars and agricultural practices (FAO, 2023). Traditional methods used pedigree and observable traits, but DNA sequencing introduced genomic insights. Genomic selection (GS) relies on DNA markers, offering advantages over traditional methods (Crossa et al., 2017).

Numerous studies have investigated the efficacy of GS compared to traditional phenotypic selection across various crops and livestock. Butoto et al. (2022) observed that both GS and phenotypic selection were equally effective in enhancing resistance to Fusarium ear rot and reducing feminizing contamination in maize. Similarly, Sallam and Smith (2016) demonstrated that integrating GS into barley breeding programs targeting yield and Fusarium head blight (FHB) resistance yielded comparable gains in selection response to traditional phenotypic methods. Moreover, GS offered the added benefits of shorter breeding cycles and reduced costs. In contrast, research in maize breeding conducted by Beyene et al. (2015) and Gesteiro et al. (2023) revealed that GS outperformed phenotypic selection, resulting in superior genetic gains. These comparative findings underscore the considerable advantages of GS in optimizing breeding outcomes across diverse agricultural settings.

GS revolutionizes plant and animal breeding by leveraging high-density markers across the genome. It operates on the principle that at least one genetic marker is in linkage disequilibrium with a causative QTL (Quantitative Trait Locus) for the desired trait (Meuwissen et al., 2001). This method transforms breeding in several ways: a) Identifying promising genotypes before planting; b) Improving precision in selecting superior individuals; c) Saving resources by reducing extensive phenotyping; d) Accelerating variety development by shortening breeding cycles; e) Intensifying selection efforts; f) Facilitating the selection of traits difficult to measure; g) Enhancing the accuracy of the selection process (Bernardo and Yu, 2007; Heffner et al., 2009; Desta and Ortiz, 2014; Abed et al., 2018; Budhlakoti et al., 2022).

The GS methodology, embraced widely, expedites genetic improvements in plant breeding programs (Desta and Ortiz, 2014; Bassi et al., 2016; Xu et al., 2020). Utilizing advanced statistical and machine learning models (Montesinos-López et al., 2022), GS efficiently selects individuals within breeding populations. Deep learning, a subset of machine learning, has also shown promise in GS (Montesinos-López et al., 2021; Wang et al., 2023). This selection process relies on data from a training population, encompassing both phenotypic and genotypic information (Crossa et al., 2017).

The Deep Neural Network Genomic Prediction (DNNGP) method of Wang et al. (2023) represents a novel advanced on deep-learning genomic predictive approach. The authors compared the DNNGP with other genomic prediction methods for various traits using genotypic and transcriptomics on maize data. They demonstrated that DNNGP outperformed GBLUP in most datasets. For instance, for maize days to anthesis (DTA) trait, DNNGP showed superiority over GBLUP by 619.840% and 16.420% using gene expression and Single Nucleotide Polymorphism (SNP) data, respectively. When utilizing genotypic data, DNNGP achieved a prediction accuracy of 0.720 for DTA, while GBLUP reached 0.580. However, the study found varied patterns in prediction accuracy for other traits.

Following rigorous training, these models utilize genotypic data to predict breeding or phenotypic values for traits within a target population (Budhlakoti et al., 2022). The GS methodology is versatile, accommodating various scenarios including multi-trait considerations (Calus and Veerkamp, 2011), known major genes and marker-trait associations, Genotype × Environment interaction (GE) (Crossa et al., 2017), and integration of other omics data (Hu et al., 2021; Wu et al., 2022) such as transcriptomics, metabolomics, and proteomics. GE influences phenotypic trait values across diverse environments, underscoring its importance in association and prediction models. Jarquin et al. (2014) introduced a framework significantly improving prediction accuracy in the presence of GE, yet without considering environmental covariates. To enhance accuracy further, recent studies are integrating environmental information into genomic prediction models.

Jarquin et al. (2014) framework lacks consideration of environmental covariates, prompting recent studies to integrate such information to enhance prediction accuracy. For instance, Montesinos-López et al. (2023) and Costa-Neto et al. (2021a, 2021b) demonstrated significant improvements. Conversely, studies by Monteverde et al. (2019); Jarquin et al. (2020), and Rogers et al. (2021) showed modest or negligible enhancements, revealing the ongoing challenge of effectively integrating environmental data into genomic prediction models.

Achieving high prediction accuracy in GS faces significant challenges due to genetic complexities, environmental variations, and data constraints (Juliana et al., 2018). Complex traits involve multiple gene influences, while environmental conditions can alter trait expression (Desta and Ortiz, 2014; Crossa et al., 2017). Phenotyping and marker data quality are critical, and issues like overfitting and population structure can compromise prediction precision (Budhlakoti et al., 2022). Ongoing research focuses on improving models, increasing marker density, and enhancing data quality to refine genomic prediction accuracy (Crossa et al., 2017; Budhlakoti et al., 2022).

Ongoing efforts focus on refining GS accuracy through various optimizations. This includes fine-tuning training and testing sets for improved precision (Rincent et al., 2012; Akdemir et al., 2015). Researchers are also evaluating diverse statistical machine learning methods to develop robust models with minimal fine-tuning yet high accuracy (Montesinos-López et al., 2022). Moreover, integrating additional omics data, such as phenomics and transcriptomics, aims to bolster GS accuracy and identify potent predictors for target traits (Montesinos-López et al., 2017; Krause et al., 2019; Monteverde et al., 2019; Hu et al., 2021; Costa-Neto et al., 2021a, b; Rogers and Holland, 2022; Wu et al., 2022). These endeavors seek to enhance GS predictive capabilities by leveraging diverse information sources.

Feature engineering (FE) is crucial in improving machine learning model performance by selecting, modifying, or creating new features from raw data. It transforms input data into a more representative and informative format, capturing relevant patterns and relationships, and enhancing the model’s generalization ability. FE involves various tasks like selecting optimal features, generating new features, normalization/scaling, handling missing values, and encoding categorical variables. For instance, techniques like Principal Component Analysis (PCA) can transform correlated features into uncorrelated ones (Lam et al., 2017; Dong and Liu, 2018; Khurana et al., 2018). FE’s popularity is rising due to its ability to enhance model performance, extract meaningful information from complex data, improve interpretability, and boost efficiency. Successful implementations include sentiment analysis, image recognition, and predictive maintenance, showcasing FE’s effectiveness across domains (Nargesian et al., 2017; Carrillo-de-Albornoz et al., 2018; Yurek and Birant, 2019). In genomic prediction, FE has also been successful, as demonstrated by Bermingham et al. (2015) and Afshar and Usefi (2020). These examples underscore FE critical role in various domains, leading to more accurate machine learning applications (Dong and Liu, 2018).

The impact of feature engineering (FE) on reducing prediction error varies depending on the dataset, problem, and quality of FE. Well-crafted features can notably minimize prediction error in some cases, but the exact improvement is context-specific and not guaranteed. Effective FE can enhance model performance significantly, albeit its extent varies case by case (Heaton, 2016; Dong and Liu, 2018).

To optimize genomic selection’s predictive accuracy, it’s vital to adopt innovative methodologies that account for its multifaceted influences. FE in genomic prediction offers a promising approach by enhancing prediction quality, uncovering genetic insights, customizing models to specific needs, improving interpretability, and minimizing data noise. In this paper, we investigate FE applied to environmental covariates to assess its potential in enhancing prediction performance within the context of genomic selection.

## Materials and methods

### Dataset USP

The University of São Paulo (USP) Maize, *Zea mays* L., dataset is sourced from germplasm developed by the Luiz de Queiroz College of Agriculture at the University of São Paulo, Brazil. An experiment was conducted between 2016 and 2017 involving 49 inbred lines, yielding a total of 906 F1 hybrids, of which 570 were assessed across eight diverse environments for grain yield (GY). These environments were created by combining two locations, two years, and two nitrogen levels. However, we specifically used data from four distinct environments for this research, each containing 100 hybrids. It’s important to note that these environments had varying soil types and climatic conditions, and the study integrated data from 248 covariates related to these environmental factors. The parent lines underwent genotyping through the Affymetrix Axiom Maize Genotyping Array, resulting in a dataset of 54,113 high-quality SNPs after applying stringent quality control procedures. Please refer to Costa-Neto et al. (2021a) for further comprehensive information on this dataset.

### Dataset Japonica

The Japonica dataset comprises 320 rice (*Oryza sativa L.*) genotypes drawn from the Japonica tropical rice population. This dataset underwent evaluations for the same four traits (GY, PHR: percentage of head rice, GC: percentage of chalky grains, PH: plant height) as the Indica population, but in this case, it was conducted across five distinct environments spanning from 2009 to 2013. Covariates were meticulously measured three times a year, covering three developmental stages (maturation, reproductive, and vegetative). This dataset comprises a non-balanced set of 1,051 assessments recorded across these five diverse environments. Additionally, each genotype within this dataset was meticulously evaluated for 16,383 SNP markers that remained after rigorous quality control procedures, with each marker being represented as 0, 1, or 2. For more comprehensive information on this dataset, please refer to Monteverde et al. (2019).

### Dataset G2F

These three distinct datasets correspond to the Maize Crop, *Zea mays* L., for years 2014 (G2F_2014), 2015 (G2F_2015), and 2016 (G2F_2016) from the Genomes to Fields maize project (Lawrence-Dill, 2017), as outlined by Rogers and Holland (2022). These datasets collectively encompass a wealth of phenotypic, genotypic, and environmental information. To narrow the focus, our analysis primarily includes four specific traits: Grain_Moisture_BLUE (GM_BLUE), Grain_Moisture_weight (GM_Weight), Yield_Mg_ha_BLUE (YM_BLUE), and Yield_Mg_ha_weight (YM_Weight), carefully selected from a larger pool of traits detailed by Rogers and Holland (2022). Across these three years, the study involves 18, 12, and 18 distinct environments for the years 2014 (G2F_2014), 2015 (G2F_2015) and 2016 (G2F_2016), respectively. Regarding genotype numbers, the dataset for 2014 consisted of 781 genotypes, the dataset for 2015 featured 1,011 genotypes, and the dataset for 2016 comprised 456 genotypes. The analysis relies on 20,373 SNP markers that have already undergone imputation and filtering, following the methodology outlined by Rogers et al. (2021) and Rogers and Holland (2022). Additive allele calls are documented as minor allele counts, represented as 0, 1, or 2. For more detailed insights into these datasets, we recommend consulting the comprehensive description provided in Lawrence-Dill (2017) and Rogers and Holland (2022).

It is worth noting that each data set presents unique sets of environments. However, concerning traits, the G2F_2014, G2F_2015, and G2F_2016 datasets share identical traits, as do the Japonica dataset.

### Statistical models

The four predictors under a genomic best linear unbiased predictor (GBLUP; Habier et al., 2007; VanRaden, 2008) model are described below.

#### Predictor P1: E+G

This predictor is represented as


(1) $$Y_{ij}=\mu +E_{i}+g_{j}+\epsilon_{ij},$$

where $Y_{ij}$ denotes the response variable in environment i and genotype j. μ denotes the population mean; $E_{i}$ are the random effects of environments, $g_{j},\;j=1,\ldots ,J$ , denotes the random effects of lines, and $\epsilon_{ij}$ denotes the random error components in the model assumed to be independent normal random variables with mean 0 and variance $\sigma^{2}$ . In the context of this predictor **E+G, *X***, denotes the matrix of markers and M the matrix of centered and standardized markers. Then $G=\frac{MM^{T}}{p}$ (VanRaden, 2008), where p is the number of markers. $Z_{g}$ is the design matrix of genotypes (lines) of order n×J, G is the genomic relationship-matrix computed using markers (VanRaden, 2008). Therefore, the random effect of lines is distributed as $ɡ={\left(g_{1},\ldots ,g_{J}\right)}^{T}∼N_{J}\left(0,\sigma_{g}^{2}Z_{g}\text{G}Z_{g}^{T}\right)$ . This model (1) was implemented in the BGLR library of Pérez and de los Campos (2014). Therefore, the linear kernel matrix for the genotype effect was determined by calculating the “covariance” structure of the genotype predictor ( $Z_{g}$ ***g***) as $K_{g}=Z_{g}\text{G}Z_{g}^{T}$ .

On the other hand, the linear kernel matrix for the Environment effect was computed using three different techniques: not using environmental covariates (NoEC), with environmental covariates (EC), and with environmental covariates with FE.

∘ **NoEC**: Under this NoEC technique, the resulting linear kernel of environments was computed as $K_{E}=X_{E}X_{E}^{T}/I$ , where I denotes the number of environments and $X_{E}$ the design matrix of environments with zeros and ones, with ones in positions of specific environments.
∘ **EC**: The EC technique involved selecting and scaling the environmental covariates (EC) that exhibited a relevant Pearson´s correlation with the response variable. Covariates are selected if their Pearson’s correlation with the response variable exceeds 0.5 in each training set per trait. Notably, covariate selection excludes response variables in the testing set, representing the environment to predict. Covariates meeting a correlation of at least 0.5 are used; otherwise, lower thresholds like 0.3 or 0.4 are considered. Correlations below these values indicate training without environmental covariates.
∘ The resulting set of selected EC’s was then used to compute an environmental linear kernel, denoted as $K_{EC}$ of order I×I . After using this kernel, the expanded environmental kernel was computed as $K_{E_{EC}}=\;X_{E}K_{EC}X_{E}^{T}/I$ , which was used in the Bayesian model. The scaling of each environmental covariate was done by subtracting its respective mean and dividing by its corresponding standard deviation.
∘ **FE**: The Feature Engineering (FE) technique involved computing various mathematical transformations between all possible pairs of ECs, including addition, difference, product, and ratio, as well as other commonly used transformations such as inverses, square powers, root squares, logarithms, and some Box-Cox transformations for each EC. These transformations were used to generate new variables through FE. The transformation of addition, difference, product and ratio were implemented for each pair of environmental covariates, that is, there were built a total the n_cov choose two new covariates, with n_cov denoting the number of environmental covariates in each data set. While with transformations such as inverses ( 1/x ), square powers ( $x^{2})$ , root squares ( $\sqrt{x})$ , natural logarithms [ ln(x) ], and Box-Cox transformations for each environmental covariate was created only one new environmental covariate. Then the original and new environmental covariates were concatenated in a matrix and then were submitted to the selection process explained above. Then under the FE approach these resulting covariates are used to compute the new environmental kernel matrix $\left(K_{E_{FE}}\right)$ .

#### Predictor P2: E+G+GE

The E+G+GE predictor is similar to P1 (Equation 1) but also accounts for the differential response of cultivars in environments, that is GE. This is achieved by taking the product of the kernel matrices of the genotype (G) and environment (E) predictors, that is, they were computed as $K_{g}{}^{\circ}K_{E_{NoEC}}$ (for NoEC), $K_{g}{}^{\circ}K_{E_{EC}}$ (for EC) or $K_{g}{}^{\circ}K_{E_{FE}}$ (for FE), which serves as the kernel matrix for the GE. In general, adding the GE interaction to the statistical machine learning model increases the genomic prediction accuracy (Jarquin et al., 2014; Crossa et al., 2017). Also, it is important to point out that under this predictor (P2) variance components and heritability of each trait in each data set were obtained under a Bayesian framework using the complete data set (i.e., no missing values allowed). For this computation all the terms were entered as random effects into the model but without taking into account the environmental covariates.

#### Predictor P3: E+G+BRR

The E+G+BRR predictor is similar to P1 (Equation 1), but incorporating the ECs as fixed effects in a Bayesian Ridge Regression (BRR) framework, that is, regression coefficients are assigned normal independent and identically distributed normal distributions, with mean zero and variance $\sigma_{\beta }^{2}$ . See details of BRR in Pérez and de los Campos (2014).

#### Predictor P4: E+G+GE+BRR

The E+G+GE+BRR predictor is similar to P2, but also incorporates ECs as fixed effects in a Bayesian Ridge Regression (BRR) framework (see **Appendix** for brief details on Bayesian Ridge Regression). The priors used for GBLUP and BRR in BGLR are those default settings which are given with details in Pérez and de los Campos (2014). In this study, we found these default settings to be suitable, as we experimented with various configurations of the prior hyperparameters for the GBLUP and BRR models on the USP and G2F_2014 datasets. Remarkably, all configurations yielded identical predictions. Consequently, for the remaining datasets, we opted to utilize only the default settings.

### Evaluation of prediction performance

The cross-validation approach used in this study involved leaving one environment out. In each iteration, the data from a single-environment served as the testing set, while the data from all other families constituted the training set (Montesinos-López et al., 2022). The number of iterations was equal to the number of environments to ensure that each environment was used as the testing set exactly one time. This method was employed to assess the model’s ability to predict information from a complete environment using data from other environments.

To evaluate the predictive performance we used the Mean Square Error (MSE) that quantifies the prediction error by measuring the squared deviation between observed and predicted values on the testing set. The MSE was computed for each scenario evaluated (NoEC, EC and FE) and then for comparing these three scenarios was computed the relative efficiencies as:


$$RE_{NoEC\_vs\_EC}=\left(\frac{MSE\left(NoEC\right)}{MSE\left(EC\right)}\right)$$

$$RE_{NoEC\_vs\_FE}=\left(\frac{MSE\left(NoEC\right)}{MSE\left(FE\right)}\right)\;$$

$$RE_{EC\_vs\_FE}=\left(\frac{MSE\left(EC\right)}{MSE\left(FE\right)}\right)\;$$

$RE_{NoEC\_vs\_EC}$ compares the prediction performance of EC vs NoEC, $RE_{NoEC\_vs\_FE}$ compares the prediction performance of FE vs NoEC and $RE_{EC\_vs\_FE}$ compares the prediction performance of FE vs EC. When $RE_{NoEC\_vs\_EC}>1$ the best prediction performance was obtained by the EC strategy, while when $RE_{NoEC\_vs\_EC}<1$ the strategy NoEC was the best. While when the relative efficiencies are equal to 1 means that both methods had equal prediction performance. The same interpretation applies for the other comparisons in terms of RE.

## Results

The results are given in three sections for three datasets (Japonica, USP and G2F_2016). For each section we provided the results for the four predictor models under study (E+G, E+G+GE, E+G+BRR, E+G+GE+BRR) and under each predictor we compared three strategies for the use of the environmental covariates: NoEC, using environmental covariables (EC) and using environmental covariables with FE. Additionally, **Appendix A** contains comprehensive details of the BRR model utilized in this study. Furthermore, **Appendix B** offers extensive information on the outcomes for Japonica, USP, and G2F_2016 datasets, which are outlined in **Table B1**–**Table B2**, **Table B3**, **Table B4**, **Table B4**–**Table B5** respectively. Additionally, **Table B7** in this appendix presents the variance components and heritability of each trait within every dataset. For the results pertaining to datasets G2F_2014 and G2F_2015, please refer to the **Supplementary Materials** section.

**Table B1:** The prediction performance and the relative efficiency (RE) for **Japonica dataset** in terms of mean squared error (MSE) for each Environment and for each trait, for the predictors E+G and E+G+GE under three different techniques to compute the Kernel for the effect of the Environment: without Environmental Covariates (NoEC), using Environmental covariates (EC) and using Environmental Covariates with Feature Engineering (FE).

Predictor	Trait	Env	NoEC	EC	FE	NoEC_vs_EC	EC_vs_FE	NoEC_vs_FE
E+G	GC	2009	0.004	0.005	0.009	0.729	0.522	0.380
E+G	GC	2010	0.011	0.017	0.013	0.663	1.287	0.853
E+G	GC	2011	0.002	0.009	0.004	0.202	2.686	0.543
E+G	GC	2012	0.028	0.039	0.034	0.719	1.140	0.819
E+G	GC	2013	0.002	0.009	0.006	0.185	1.586	0.293
E+G	GC	Across	–	–	–	0.500	1.444	0.578
E+G	GY	2009	3049246.325	796963.009	1025847.337	3.826	0.777	2.972
E+G	GY	2010	5683515.755	2488872.780	3046722.045	2.284	0.817	1.866
E+G	GY	2011	4024422.454	2853854.731	2615758.363	1.410	1.091	1.539
E+G	GY	2012	2050745.031	1157280.313	1436429.272	1.772	0.806	1.428
E+G	GY	2013	405886.860	410565.496	377719.356	0.989	1.087	1.075
E+G	GY	Across	–	–	–	2.056	0.916	1.776
E+G	PH	2009	58.674	16.561	15.872	3.543	1.043	3.697
E+G	PH	2010	27.005	12.127	10.959	2.227	1.107	2.464
E+G	PH	2011	13.534	28.641	28.573	0.473	1.002	0.474
E+G	PH	2012	175.254	168.840	164.039	1.038	1.029	1.068
E+G	PH	2013	18.363	23.009	22.729	0.798	1.012	0.808
E+G	PH	Across	–	–	–	1.616	1.039	1.702
E+G	PHR	2009	0.001	0.001	0.001	0.750	1.333	1.000
E+G	PHR	2010	0.001	0.002	0.001	0.750	1.600	1.200
E+G	PHR	2011	0.002	0.001	0.002	1.643	0.778	1.278
E+G	PHR	2012	0.006	0.007	0.006	0.797	1.095	0.873
E+G	PHR	2013	0.001	0.001	0.001	0.750	1.333	1.000
E+G	PHR	Across	–	–	–	0.938	1.228	1.070
E+G+GE	GC	2009	0.001	0.001	0.003	0.769	0.433	0.333
E+G+GE	GC	2010	0.013	0.034	0.032	0.394	1.053	0.414
E+G+GE	GC	2011	0.002	0.006	0.003	0.281	2.462	0.692
E+G+GE	GC	2012	0.025	0.025	0.029	1.004	0.839	0.843
E+G+GE	GC	2013	0.006	0.003	0.003	2.148	1.039	2.231
E+G+GE	GC	Across	–	–	–	0.919	1.165	0.903
E+G+GE	GY	2009	3242702.030	1152261.036	1460144.165	2.814	0.789	2.221
E+G+GE	GY	2010	4339466.437	3653811.519	4302236.223	1.188	0.849	1.009
E+G+GE	GY	2011	1834248.259	3337540.514	3251492.136	0.550	1.027	0.564
E+G+GE	GY	2012	1894112.619	989127.176	1358843.398	1.915	0.728	1.394
E+G+GE	GY	2013	1924915.862	416054.225	370980.321	4.627	1.122	5.189
E+G+GE	GY	Across	–	–	–	2.219	0.903	2.075
E+G+GE	PH	2009	56.517	20.261	17.631	2.789	1.149	3.206
E+G+GE	PH	2010	17.957	12.954	16.142	1.386	0.803	1.112
E+G+GE	PH	2011	44.689	77.310	64.564	0.578	1.197	0.692
E+G+GE	PH	2012	164.891	175.005	168.680	0.942	1.038	0.978
E+G+GE	PH	2013	59.136	24.696	23.544	2.395	1.049	2.512
E+G+GE	PH	Across	–	–	–	1.618	1.047	1.700
E+G+GE	PHR	2009	0.001	0.001	0.001	0.750	1.333	1.000
E+G+GE	PHR	2010	0.002	0.002	0.002	0.818	1.467	1.200
E+G+GE	PHR	2011	0.002	0.001	0.001	1.727	0.917	1.583
E+G+GE	PHR	2012	0.005	0.007	0.006	0.783	1.095	0.857
E+G+GE	PHR	2013	0.001	0.001	0.001	0.750	1.600	1.200
E+G+GE	PHR	Across	–	–	–	0.966	1.282	1.168

**Table B2:** The prediction performance and the relative efficiency (RE) for Japonica dataset in terms of mean squared error (MSE) for each Environment and for each trait, for the predictors E+G+BRR and E+G+GE+BRR under three different techniques to compute the Kernel for the effect of the Environment: without Environmental Covariates (NoEC), using Environmental covariates (EC) and using Environmental Covariate*s with Feature Engineering (FE)*.

Predictor	Trait	Env	NoEC	EC	FE	NoEC_vs_EC	EC_vs_FE	NoEC_vs_FE
E+G+BRR	GC	2009	0.004	0.008	0.007	0.417	1.151	0.480
E+G+BRR	GC	2010	0.011	0.009	0.007	1.196	1.353	1.618
E+G+BRR	GC	2011	0.002	0.009	0.005	0.207	2.044	0.422
E+G+BRR	GC	2012	0.028	0.010	0.010	2.755	1.063	2.927
E+G+BRR	GC	2013	0.002	0.002	0.003	0.944	0.529	0.500
E+G+BRR	GC	Across	–	–	–	1.104	1.228	1.189
E+G+BRR	GY	2009	3049246.325	1221342.669	1607864.482	2.497	0.760	1.897
E+G+BRR	GY	2010	5683515.755	7662804.296	7449307.222	0.742	1.029	0.763
E+G+BRR	GY	2011	4024422.454	3689043.326	3841776.983	1.091	0.960	1.048
E+G+BRR	GY	2012	2050745.031	2749626.084	5697594.878	0.746	0.483	0.360
E+G+BRR	GY	2013	405886.860	743092.012	988735.462	0.546	0.752	0.411
E+G+BRR	GY	Across	–	–	–	1.124	0.797	0.896
E+G+BRR	PH	2009	58.674	15.466	15.281	3.794	1.012	3.840
E+G+BRR	PH	2010	27.005	22.962	27.436	1.176	0.837	0.984
E+G+BRR	PH	2011	13.534	29.033	25.921	0.466	1.120	0.522
E+G+BRR	PH	2012	175.254	165.479	159.312	1.059	1.039	1.100
E+G+BRR	PH	2013	18.363	10.981	14.450	1.672	0.760	1.271
E+G+BRR	PH	Across	–	–	–	1.634	0.954	1.543
E+G+BRR	PHR	2009	0.001	0.001	0.001	1.000	1.000	1.000
E+G+BRR	PHR	2010	0.001	0.001	0.001	1.714	1.167	2.000
E+G+BRR	PHR	2011	0.002	0.001	0.001	2.875	0.889	2.556
E+G+BRR	PHR	2012	0.006	0.006	0.011	0.887	0.554	0.491
E+G+BRR	PHR	2013	0.001	0.001	0.001	1.200	1.000	1.200
E+G+BRR	PHR	Across	–	–	–	1.535	0.922	1.449
E+G+GE+BRR	GC	2009	0.001	0.007	0.006	0.154	1.083	0.167
E+G+GE+BRR	GC	2010	0.013	0.017	0.008	0.796	2.012	1.602
E+G+GE+BRR	GC	2011	0.002	0.007	0.003	0.273	2.000	0.546
E+G+GE+BRR	GC	2012	0.025	0.024	0.019	1.029	1.278	1.316
E+G+GE+BRR	GC	2013	0.006	0.004	0.002	1.526	2.111	3.222
E+G+GE+BRR	GC	Across	–	–	–	0.756	1.697	1.371
E+G+GE+BRR	GY	2009	3242702.030	1333530.864	1860560.276	2.432	0.717	1.743
E+G+GE+BRR	GY	2010	4339466.437	7649947.049	7468881.672	0.567	1.024	0.581
E+G+GE+BRR	GY	2011	1834248.259	4157537.398	4872981.083	0.441	0.853	0.376
E+G+GE+BRR	GY	2012	1894112.619	1690390.524	4082192.704	1.121	0.414	0.464
E+G+GE+BRR	GY	2013	1924915.862	584945.854	681359.148	3.291	0.859	2.825
E+G+GE+BRR	GY	Across	–	–	–	1.570	0.773	1.198
E+G+GE+BRR	PH	2009	56.517	18.089	17.332	3.124	1.044	3.261
E+G+GE+BRR	PH	2010	17.957	14.956	26.970	1.201	0.555	0.666
E+G+GE+BRR	PH	2011	44.689	22.351	22.026	1.999	1.015	2.029
E+G+GE+BRR	PH	2012	164.891	171.095	167.745	0.964	1.020	0.983
E+G+GE+BRR	PH	2013	59.136	11.071	12.138	5.342	0.912	4.872
E+G+GE+BRR	PH	Across	–	–	–	2.526	0.909	2.362
E+G+GE+BRR	PHR	2009	0.001	0.001	0.001	1.000	1.000	1.000
E+G+GE+BRR	PHR	2010	0.002	0.001	0.001	2.571	1.000	2.571
E+G+GE+BRR	PHR	2011	0.002	0.001	0.001	2.375	1.000	2.375
E+G+GE+BRR	PHR	2012	0.005	0.006	0.011	0.871	0.554	0.482
E+G+GE+BRR	PHR	2013	0.001	0.001	0.001	1.200	0.833	1.000
E+G+GE+BRR	PHR	Across	–	–	–	1.604	0.877	1.486

**Table B3:** The prediction performance and the relative efficiency (RE) for **USP dataset** in terms of mean squared error (MSE) for each Environment and for each trait, for the predictors E+G and E+G+GE under three different techniques to compute the Kernel for the effect of the Environment: without Environmental Covariates (NoEC), using Environmental covariates (EC) and using Environmental Covariates with Feature Engineering (FE).

Predictor	Trait	Env	NoEC	EC	FE	NoEC_vs_EC	EC_vs_FE	NoEC_vs_FE
E+G	GY	Env1	3.476	3.281	3.141	1.059	1.045	1.107
E+G	GY	Env2	4.073	4.689	4.475	0.869	1.048	0.910
E+G	GY	Env3	5.246	6.317	6.455	0.831	0.979	0.813
E+G	GY	Env4	8.174	7.814	8.262	1.046	0.946	0.989
E+G	GY	Across	–	–	–	0.951	1.004	0.955
E+G+GE	GY	Env1	3.254	2.939	2.789	1.107	1.054	1.167
E+G+GE	GY	Env2	4.708	4.898	4.636	0.961	1.057	1.016
E+G+GE	GY	Env3	5.833	6.307	6.396	0.925	0.986	0.912
E+G+GE	GY	Env4	8.730	7.792	8.206	1.120	0.950	1.064
E+G+GE	GY	Across	–	–	–	1.028	1.012	1.040

**Table B4:** The prediction performance and the relative efficiency (RE) for **USP dataset** in terms of mean squared error (MSE) for each Environment and for each trait, for the predictors E+G+BRR and E+G+GE+BRR under three different techniques to compute the Kernel for the effect of the Environment: without Environmental Covariates (NoEC), using Environmental covariates (EC) and using Environmental Covariates with Feature Engineering (FE).

Predictor	Trait	Env	NoEC	EC	FE	NoEC_vs_EC	EC_vs_FE	NoEC_vs_FE
E+G+BRR	GY	Env1	3.476	2.968	2.859	1.171	1.038	1.216
E+G+BRR	GY	Env2	4.073	4.951	5.301	0.823	0.934	0.768
E+G+BRR	GY	Env3	5.246	6.279	4.413	0.836	1.423	1.189
E+G+BRR	GY	Env4	8.174	5.638	5.696	1.450	0.990	1.435
E+G+BRR	GY	Across	–	–	–	1.070	1.096	1.152
E+G+GE+BRR	GY	Env1	3.254	2.769	2.644	1.175	1.047	1.231
E+G+GE+BRR	GY	Env2	4.708	4.917	5.224	0.958	0.941	0.901
E+G+GE+BRR	GY	Env3	5.833	6.373	4.265	0.915	1.494	1.368
E+G+GE+BRR	GY	Env4	8.730	5.958	5.856	1.465	1.017	1.491
E+G+GE+BRR	GY	Across	–	–	–	1.128	1.125	1.248

**Table B5:** The prediction performance and the relative efficiency (RE) for **G2F_2016 dataset** in terms of mean squared error (MSE) for each Environment and for each trait, for the predictors E+G and E+G+GE under three different techniques to compute the Kernel for the effect of the Environment: without Environmental Covariates (NoEC), using Environmental covariates (EC) and using Environmental Covariates with Feature Engineering (FE).

Predictor	Trait	Env	NoEC	EC	FE	NoEC_vs_EC	EC_vs_FE	NoEC_vs_FE
E+G	Grain_Moisture_BLUE	ARH1_2016	1.733	6.108	1.886	0.284	3.238	0.919
E+G	Grain_Moisture_BLUE	DEH1_2016	7.863	5.829	4.645	1.349	1.255	1.693
E+G	Grain_Moisture_BLUE	GAH1_2016	6.686	5.107	2.154	1.309	2.371	3.105
E+G	Grain_Moisture_BLUE	IAH1_2016	9.814	7.419	2.703	1.323	2.745	3.632
E+G	Grain_Moisture_BLUE	IAH2_2016	3.124	0.866	1.694	3.608	0.511	1.844
E+G	Grain_Moisture_BLUE	IAH3_2016	1.456	2.981	1.486	0.489	2.006	0.980
E+G	Grain_Moisture_BLUE	IAH4_2016	2.495	0.506	0.467	4.932	1.084	5.344
E+G	Grain_Moisture_BLUE	ILH1_2016	4.556	3.436	9.783	1.326	0.351	0.466
E+G	Grain_Moisture_BLUE	INH1_2016	1.934	9.982	2.887	0.194	3.457	0.670
E+G	Grain_Moisture_BLUE	MIH1_2016	2.988	3.101	3.366	0.963	0.922	0.888
E+G	Grain_Moisture_BLUE	MNH1_2016	17.117	4.471	4.483	3.829	0.997	3.818
E+G	Grain_Moisture_BLUE	MOH1_2016	0.809	3.068	0.668	0.264	4.593	1.211
E+G	Grain_Moisture_BLUE	NCH1_2016	21.208	10.860	3.598	1.953	3.018	5.895
E+G	Grain_Moisture_BLUE	NEH1_2016	6.193	4.897	10.060	1.265	0.487	0.616
E+G	Grain_Moisture_BLUE	NYH2_2016	7.475	3.625	2.092	2.062	1.732	3.573
E+G	Grain_Moisture_BLUE	OHH1_2016	4.840	2.834	5.728	1.708	0.495	0.845
E+G	Grain_Moisture_BLUE	WIH1_2016	5.143	2.599	3.788	1.979	0.686	1.358
E+G	Grain_Moisture_BLUE	WIH2_2016	4.219	6.224	1.601	0.678	3.887	2.634
E+G	Grain_Moisture_BLUE	Across	–	–	–	1.640	1.880	2.194
E+G	Grain_Moisture_weight	ARH1_2016	30.391	24.235	30.934	1.254	0.783	0.982
E+G	Grain_Moisture_weight	DEH1_2016	14.987	0.207	2.910	72.261	0.071	5.150
E+G	Grain_Moisture_weight	GAH1_2016	1.272	2.339	7.133	0.544	0.328	0.178
E+G	Grain_Moisture_weight	IAH1_2016	401.574	481.573	510.263	0.834	0.944	0.787
E+G	Grain_Moisture_weight	IAH2_2016	6.212	2.568	25.510	2.419	0.101	0.244
E+G	Grain_Moisture_weight	IAH3_2016	0.199	10.913	31.831	0.018	0.343	0.006
E+G	Grain_Moisture_weight	IAH4_2016	311.023	244.775	180.018	1.271	1.360	1.728
E+G	Grain_Moisture_weight	ILH1_2016	5.447	2.172	25.900	2.507	0.084	0.210
E+G	Grain_Moisture_weight	INH1_2016	1.274	0.210	0.325	6.058	0.647	3.916
E+G	Grain_Moisture_weight	MIH1_2016	0.715	8.311	0.872	0.086	9.531	0.820
E+G	Grain_Moisture_weight	MNH1_2016	7.866	43.427	6.379	0.181	6.808	1.233
E+G	Grain_Moisture_weight	MOH1_2016	27.122	7.450	44.113	3.640	0.169	0.615
E+G	Grain_Moisture_weight	NCH1_2016	1.174	4.278	10.042	0.274	0.426	0.117
E+G	Grain_Moisture_weight	NEH1_2016	42.758	63.944	64.869	0.669	0.986	0.659
E+G	Grain_Moisture_weight	NYH2_2016	1.893	2.551	11.545	0.742	0.221	0.164
E+G	Grain_Moisture_weight	OHH1_2016	63.776	0.454	27.250	140.383	0.017	2.340
E+G	Grain_Moisture_weight	WIH1_2016	1.373	7.073	1.371	0.194	5.160	1.001
E+G	Grain_Moisture_weight	WIH2_2016	16.972	0.194	0.403	87.485	0.482	42.125
E+G	Grain_Moisture_weight	Across	–	–	–	17.823	1.581	3.460
E+G	Yield_Mg_ha_BLUE	ARH1_2016	3.713	3.199	14.552	1.161	0.220	0.255
E+G	Yield_Mg_ha_BLUE	DEH1_2016	5.330	3.354	4.354	1.589	0.770	1.224
E+G	Yield_Mg_ha_BLUE	GAH1_2016	3.580	10.264	4.606	0.349	2.229	0.777
E+G	Yield_Mg_ha_BLUE	IAH1_2016	3.187	2.897	1.395	1.100	2.077	2.286
E+G	Yield_Mg_ha_BLUE	IAH2_2016	7.921	7.684	8.073	1.031	0.952	0.981
E+G	Yield_Mg_ha_BLUE	IAH3_2016	5.918	4.741	3.772	1.248	1.257	1.569
E+G	Yield_Mg_ha_BLUE	IAH4_2016	2.576	2.718	3.708	0.948	0.733	0.695
E+G	Yield_Mg_ha_BLUE	ILH1_2016	8.719	4.698	6.260	1.856	0.750	1.393
E+G	Yield_Mg_ha_BLUE	INH1_2016	2.415	3.018	2.406	0.800	1.254	1.004
E+G	Yield_Mg_ha_BLUE	MIH1_2016	4.045	5.627	16.686	0.719	0.337	0.242
E+G	Yield_Mg_ha_BLUE	MNH1_2016	1.268	1.301	1.270	0.975	1.025	0.999
E+G	Yield_Mg_ha_BLUE	MOH1_2016	7.968	4.191	10.428	1.901	0.402	0.764
E+G	Yield_Mg_ha_BLUE	NCH1_2016	4.467	10.571	3.293	0.423	3.211	1.357
E+G	Yield_Mg_ha_BLUE	NEH1_2016	4.993	4.832	4.188	1.033	1.154	1.192
E+G	Yield_Mg_ha_BLUE	NYH2_2016	16.252	22.790	16.626	0.713	1.371	0.978
E+G	Yield_Mg_ha_BLUE	OHH1_2016	1.830	4.790	2.558	0.382	1.872	0.715
E+G	Yield_Mg_ha_BLUE	WIH1_2016	3.665	5.021	3.785	0.730	1.326	0.968
E+G	Yield_Mg_ha_BLUE	WIH2_2016	4.630	4.588	5.420	1.009	0.846	0.854
E+G	Yield_Mg_ha_BLUE	Across	–	–	–	0.998	1.210	1.014
E+G	Yield_Mg_ha_weight	ARH1_2016	0.989	1.000	1.542	0.989	0.649	0.641
E+G	Yield_Mg_ha_weight	DEH1_2016	0.163	0.078	0.230	2.088	0.340	0.710
E+G	Yield_Mg_ha_weight	GAH1_2016	0.035	0.439	0.288	0.079	1.522	0.120
E+G	Yield_Mg_ha_weight	IAH1_2016	3.743	3.345	3.151	1.119	1.061	1.188
E+G	Yield_Mg_ha_weight	IAH2_2016	0.175	0.668	0.077	0.262	8.629	2.261
E+G	Yield_Mg_ha_weight	IAH3_2016	0.788	1.704	1.583	0.462	1.076	0.498
E+G	Yield_Mg_ha_weight	IAH4_2016	0.498	0.091	0.105	5.491	0.861	4.729
E+G	Yield_Mg_ha_weight	ILH1_2016	1.113	0.351	0.754	3.172	0.465	1.476
E+G	Yield_Mg_ha_weight	INH1_2016	0.055	0.077	0.052	0.709	1.486	1.054
E+G	Yield_Mg_ha_weight	MIH1_2016	0.121	0.116	0.132	1.042	0.875	0.912
E+G	Yield_Mg_ha_weight	MNH1_2016	0.393	0.391	0.711	1.005	0.551	0.553
E+G	Yield_Mg_ha_weight	MOH1_2016	0.232	1.501	0.172	0.155	8.721	1.348
E+G	Yield_Mg_ha_weight	NCH1_2016	0.083	0.343	0.085	0.241	4.062	0.978
E+G	Yield_Mg_ha_weight	NEH1_2016	0.036	0.038	0.029	0.963	1.279	1.231
E+G	Yield_Mg_ha_weight	NYH2_2016	0.402	0.087	0.139	4.601	0.630	2.899
E+G	Yield_Mg_ha_weight	OHH1_2016	0.533	1.326	0.876	0.402	1.514	0.608
E+G	Yield_Mg_ha_weight	WIH1_2016	0.117	0.063	0.209	1.877	0.299	0.561
E+G	Yield_Mg_ha_weight	WIH2_2016	0.055	0.019	0.180	2.860	0.107	0.306
E+G	Yield_Mg_ha_weight	Across	–	–	–	1.529	1.896	1.226
E+G+GE	Grain_Moisture_BLUE	ARH1_2016	2.003	6.545	4.641	0.306	1.410	0.432
E+G+GE	Grain_Moisture_BLUE	DEH1_2016	5.256	5.689	10.400	0.924	0.547	0.505
E+G+GE	Grain_Moisture_BLUE	GAH1_2016	5.841	3.993	2.715	1.463	1.471	2.152
E+G+GE	Grain_Moisture_BLUE	IAH1_2016	2.857	5.585	3.541	0.512	1.577	0.807
E+G+GE	Grain_Moisture_BLUE	IAH2_2016	0.713	1.504	1.785	0.475	0.843	0.400
E+G+GE	Grain_Moisture_BLUE	IAH3_2016	2.933	4.648	2.860	0.631	1.625	1.025
E+G+GE	Grain_Moisture_BLUE	IAH4_2016	1.622	0.519	0.695	3.123	0.747	2.333
E+G+GE	Grain_Moisture_BLUE	ILH1_2016	8.071	4.093	9.622	1.972	0.425	0.839
E+G+GE	Grain_Moisture_BLUE	INH1_2016	5.315	10.531	4.891	0.505	2.153	1.087
E+G+GE	Grain_Moisture_BLUE	MIH1_2016	2.448	3.313	5.501	0.739	0.602	0.445
E+G+GE	Grain_Moisture_BLUE	MNH1_2016	13.571	5.813	6.414	2.335	0.906	2.116
E+G+GE	Grain_Moisture_BLUE	MOH1_2016	3.450	5.296	1.357	0.651	3.904	2.543
E+G+GE	Grain_Moisture_BLUE	NCH1_2016	14.869	8.231	2.333	1.806	3.528	6.374
E+G+GE	Grain_Moisture_BLUE	NEH1_2016	12.527	5.166	10.466	2.425	0.494	1.197
E+G+GE	Grain_Moisture_BLUE	NYH2_2016	9.727	4.423	5.172	2.199	0.855	1.881
E+G+GE	Grain_Moisture_BLUE	OHH1_2016	6.975	2.849	6.176	2.448	0.461	1.129
E+G+GE	Grain_Moisture_BLUE	WIH1_2016	6.024	3.056	5.975	1.971	0.512	1.008
E+G+GE	Grain_Moisture_BLUE	WIH2_2016	21.532	6.235	1.739	3.454	3.585	12.382
E+G+GE	Grain_Moisture_BLUE	Across	–	–	–	1.552	1.425	2.147
E+G+GE	Grain_Moisture_weight	ARH1_2016	14.116	3.206	9.123	4.403	0.351	1.547
E+G+GE	Grain_Moisture_weight	DEH1_2016	1.608	10.772	0.132	0.149	81.919	12.231
E+G+GE	Grain_Moisture_weight	GAH1_2016	0.862	0.883	4.521	0.976	0.195	0.191
E+G+GE	Grain_Moisture_weight	IAH1_2016	501.269	514.108	546.300	0.975	0.941	0.918
E+G+GE	Grain_Moisture_weight	IAH2_2016	43.354	23.631	36.310	1.835	0.651	1.194
E+G+GE	Grain_Moisture_weight	IAH3_2016	11.456	7.015	0.418	1.633	16.769	27.387
E+G+GE	Grain_Moisture_weight	IAH4_2016	265.697	167.322	139.446	1.588	1.200	1.905
E+G+GE	Grain_Moisture_weight	ILH1_2016	35.818	2.973	32.902	12.047	0.090	1.089
E+G+GE	Grain_Moisture_weight	INH1_2016	51.327	1.919	3.812	26.741	0.504	13.465
E+G+GE	Grain_Moisture_weight	MIH1_2016	18.430	38.977	1.668	0.473	23.368	11.049
E+G+GE	Grain_Moisture_weight	MNH1_2016	11.304	39.937	1.316	0.283	30.345	8.589
E+G+GE	Grain_Moisture_weight	MOH1_2016	3.665	14.395	291.204	0.255	0.049	0.013
E+G+GE	Grain_Moisture_weight	NCH1_2016	7.758	7.873	6.953	0.985	1.132	1.116
E+G+GE	Grain_Moisture_weight	NEH1_2016	113.669	88.519	99.451	1.284	0.890	1.143
E+G+GE	Grain_Moisture_weight	NYH2_2016	80.595	16.174	5.565	4.983	2.906	14.482
E+G+GE	Grain_Moisture_weight	OHH1_2016	12.108	0.596	0.195	20.319	3.054	62.060
E+G+GE	Grain_Moisture_weight	WIH1_2016	11.902	4.475	1.508	2.660	2.967	7.892
E+G+GE	Grain_Moisture_weight	WIH2_2016	0.917	1.365	3.320	0.672	0.411	0.276
E+G+GE	Grain_Moisture_weight	Across	–	–	–	4.570	9.319	9.253
E+G+GE	Yield_Mg_ha_BLUE	ARH1_2016	3.928	2.896	14.301	1.357	0.203	0.275
E+G+GE	Yield_Mg_ha_BLUE	DEH1_2016	5.964	3.522	3.831	1.694	0.919	1.557
E+G+GE	Yield_Mg_ha_BLUE	GAH1_2016	3.379	10.667	4.157	0.317	2.566	0.813
E+G+GE	Yield_Mg_ha_BLUE	IAH1_2016	2.287	2.778	2.820	0.823	0.985	0.811
E+G+GE	Yield_Mg_ha_BLUE	IAH2_2016	7.505	8.311	7.733	0.903	1.075	0.971
E+G+GE	Yield_Mg_ha_BLUE	IAH3_2016	7.908	6.619	5.280	1.195	1.254	1.498
E+G+GE	Yield_Mg_ha_BLUE	IAH4_2016	2.565	2.895	3.811	0.886	0.760	0.673
E+G+GE	Yield_Mg_ha_BLUE	ILH1_2016	8.036	4.761	5.919	1.688	0.804	1.358
E+G+GE	Yield_Mg_ha_BLUE	INH1_2016	6.533	2.424	1.994	2.696	1.216	3.277
E+G+GE	Yield_Mg_ha_BLUE	MIH1_2016	4.748	7.252	19.667	0.655	0.369	0.241
E+G+GE	Yield_Mg_ha_BLUE	MNH1_2016	1.422	1.479	1.265	0.961	1.169	1.124
E+G+GE	Yield_Mg_ha_BLUE	MOH1_2016	12.381	5.928	9.392	2.089	0.631	1.318
E+G+GE	Yield_Mg_ha_BLUE	NCH1_2016	5.713	11.008	3.515	0.519	3.132	1.626
E+G+GE	Yield_Mg_ha_BLUE	NEH1_2016	5.446	5.707	5.214	0.954	1.095	1.045
E+G+GE	Yield_Mg_ha_BLUE	NYH2_2016	17.271	24.594	19.504	0.702	1.261	0.886
E+G+GE	Yield_Mg_ha_BLUE	OHH1_2016	2.503	4.763	2.138	0.526	2.227	1.171
E+G+GE	Yield_Mg_ha_BLUE	WIH1_2016	2.210	5.855	3.805	0.378	1.539	0.581
E+G+GE	Yield_Mg_ha_BLUE	WIH2_2016	4.667	4.667	5.288	1.000	0.883	0.883
E+G+GE	Yield_Mg_ha_BLUE	Across	–	–	–	1.075	1.227	1.117
E+G+GE	Yield_Mg_ha_weight	ARH1_2016	2.359	1.339	1.540	1.762	0.869	1.532
E+G+GE	Yield_Mg_ha_weight	DEH1_2016	0.051	0.124	0.284	0.410	0.437	0.179
E+G+GE	Yield_Mg_ha_weight	GAH1_2016	0.026	0.357	0.258	0.074	1.385	0.102
E+G+GE	Yield_Mg_ha_weight	IAH1_2016	2.914	3.540	3.508	0.823	1.009	0.831
E+G+GE	Yield_Mg_ha_weight	IAH2_2016	0.069	0.410	0.076	0.168	5.378	0.903
E+G+GE	Yield_Mg_ha_weight	IAH3_2016	0.670	0.608	1.186	1.102	0.513	0.565
E+G+GE	Yield_Mg_ha_weight	IAH4_2016	0.199	0.110	0.082	1.807	1.343	2.426
E+G+GE	Yield_Mg_ha_weight	ILH1_2016	0.751	0.468	0.539	1.605	0.868	1.394
E+G+GE	Yield_Mg_ha_weight	INH1_2016	0.112	0.056	0.046	1.981	1.227	2.429
E+G+GE	Yield_Mg_ha_weight	MIH1_2016	0.055	0.189	0.172	0.291	1.098	0.320
E+G+GE	Yield_Mg_ha_weight	MNH1_2016	0.146	0.352	0.502	0.415	0.701	0.291
E+G+GE	Yield_Mg_ha_weight	MOH1_2016	0.283	0.295	0.263	0.959	1.122	1.076
E+G+GE	Yield_Mg_ha_weight	NCH1_2016	0.113	0.388	0.104	0.292	3.730	1.090
E+G+GE	Yield_Mg_ha_weight	NEH1_2016	0.033	0.073	0.081	0.458	0.900	0.412
E+G+GE	Yield_Mg_ha_weight	NYH2_2016	0.449	0.781	0.709	0.575	1.102	0.633
E+G+GE	Yield_Mg_ha_weight	OHH1_2016	1.202	1.667	1.328	0.721	1.255	0.905
E+G+GE	Yield_Mg_ha_weight	WIH1_2016	0.204	0.096	0.132	2.125	0.729	1.550
E+G+GE	Yield_Mg_ha_weight	WIH2_2016	0.185	0.091	0.204	2.036	0.443	0.903
E+G+GE	Yield_Mg_ha_weight	Across	–	–	–	0.978	1.339	0.974

**Table B6:** The prediction performance and the relative efficiency (RE) for **G2F_2016 dataset** in terms of mean squared error (MSE) for each Environment and for each trait, for the predictor E+G+BRR and E+G+GE+BRR under three different techniques to compute the Kernel for the effect of the Environment: without Environmental Covariates (NoEC), using Environmental covariates (EC) and using Environmental Covariates with Feature Engineering (FE).

Predictor	Trait	Env	NoEC	EC	FE	NoEC_vs_EC	EC_vs_FE	NoEC_vs_FE
E+G+BRR	Grain_Moisture_BLUE	ARH1_2016	1.733	8.086	2.856	0.214	2.832	0.607
E+G+BRR	Grain_Moisture_BLUE	DEH1_2016	7.863	5.151	4.376	1.526	1.177	1.797
E+G+BRR	Grain_Moisture_BLUE	GAH1_2016	6.686	5.025	2.002	1.331	2.511	3.341
E+G+BRR	Grain_Moisture_BLUE	IAH1_2016	9.814	3.372	2.036	2.911	1.656	4.821
E+G+BRR	Grain_Moisture_BLUE	IAH2_2016	3.124	1.172	1.650	2.665	0.711	1.894
E+G+BRR	Grain_Moisture_BLUE	IAH3_2016	1.456	2.060	1.237	0.707	1.665	1.178
E+G+BRR	Grain_Moisture_BLUE	IAH4_2016	2.495	0.515	0.496	4.845	1.039	5.031
E+G+BRR	Grain_Moisture_BLUE	ILH1_2016	4.556	2.970	9.745	1.534	0.305	0.468
E+G+BRR	Grain_Moisture_BLUE	INH1_2016	1.934	12.122	2.526	0.160	4.798	0.766
E+G+BRR	Grain_Moisture_BLUE	MIH1_2016	2.988	3.562	3.335	0.839	1.068	0.896
E+G+BRR	Grain_Moisture_BLUE	MNH1_2016	17.117	3.852	3.685	4.444	1.045	4.645
E+G+BRR	Grain_Moisture_BLUE	MOH1_2016	0.809	1.362	0.678	0.594	2.009	1.193
E+G+BRR	Grain_Moisture_BLUE	NCH1_2016	21.208	10.060	3.499	2.108	2.875	6.061
E+G+BRR	Grain_Moisture_BLUE	NEH1_2016	6.193	2.846	9.795	2.176	0.291	0.632
E+G+BRR	Grain_Moisture_BLUE	NYH2_2016	7.475	2.344	2.213	3.189	1.059	3.378
E+G+BRR	Grain_Moisture_BLUE	OHH1_2016	4.840	2.898	5.870	1.670	0.494	0.825
E+G+BRR	Grain_Moisture_BLUE	WIH1_2016	5.143	3.045	4.014	1.689	0.759	1.281
E+G+BRR	Grain_Moisture_BLUE	WIH2_2016	4.219	6.235	1.648	0.677	3.785	2.560
E+G+BRR	Grain_Moisture_BLUE	Across	–	–	–	1.849	1.671	2.299
E+G+BRR	Grain_Moisture_weight	ARH1_2016	30.391	7.962	7.088	3.817	1.123	4.288
E+G+BRR	Grain_Moisture_weight	DEH1_2016	14.987	0.443	5.442	33.869	0.081	2.754
E+G+BRR	Grain_Moisture_weight	GAH1_2016	1.272	5.393	2.233	0.236	2.415	0.569
E+G+BRR	Grain_Moisture_weight	IAH1_2016	401.574	459.125	508.319	0.875	0.903	0.790
E+G+BRR	Grain_Moisture_weight	IAH2_2016	6.212	1.611	176.584	3.855	0.009	0.035
E+G+BRR	Grain_Moisture_weight	IAH3_2016	0.199	51.438	110.303	0.004	0.466	0.002
E+G+BRR	Grain_Moisture_weight	IAH4_2016	311.023	188.044	160.261	1.654	1.173	1.941
E+G+BRR	Grain_Moisture_weight	ILH1_2016	5.447	22.946	64.425	0.237	0.356	0.085
E+G+BRR	Grain_Moisture_weight	INH1_2016	1.274	0.691	0.685	1.843	1.009	1.860
E+G+BRR	Grain_Moisture_weight	MIH1_2016	0.715	31.083	1.554	0.023	20.002	0.460
E+G+BRR	Grain_Moisture_weight	MNH1_2016	7.866	43.882	6.124	0.179	7.165	1.284
E+G+BRR	Grain_Moisture_weight	MOH1_2016	27.122	21.394	393.212	1.268	0.054	0.069
E+G+BRR	Grain_Moisture_weight	NCH1_2016	1.174	5.985	24.041	0.196	0.249	0.049
E+G+BRR	Grain_Moisture_weight	NEH1_2016	42.758	57.295	90.340	0.746	0.634	0.473
E+G+BRR	Grain_Moisture_weight	NYH2_2016	1.893	0.666	46.015	2.842	0.015	0.041
E+G+BRR	Grain_Moisture_weight	OHH1_2016	63.776	7.228	19.206	8.823	0.376	3.321
E+G+BRR	Grain_Moisture_weight	WIH1_2016	1.373	13.412	1.266	0.102	10.595	1.084
E+G+BRR	Grain_Moisture_weight	WIH2_2016	16.972	7.246	4.891	2.342	1.482	3.470
E+G+BRR	Grain_Moisture_weight	Across	–	–	–	3.495	2.673	1.254
E+G+BRR	Yield_Mg_ha_BLUE	ARH1_2016	3.713	3.799	14.569	0.977	0.261	0.255
E+G+BRR	Yield_Mg_ha_BLUE	DEH1_2016	5.330	2.922	3.946	1.824	0.740	1.351
E+G+BRR	Yield_Mg_ha_BLUE	GAH1_2016	3.580	11.055	5.613	0.324	1.970	0.638
E+G+BRR	Yield_Mg_ha_BLUE	IAH1_2016	3.187	1.743	1.393	1.829	1.252	2.289
E+G+BRR	Yield_Mg_ha_BLUE	IAH2_2016	7.921	7.568	8.528	1.047	0.888	0.929
E+G+BRR	Yield_Mg_ha_BLUE	IAH3_2016	5.918	5.873	6.247	1.008	0.940	0.947
E+G+BRR	Yield_Mg_ha_BLUE	IAH4_2016	2.576	2.618	3.773	0.984	0.694	0.683
E+G+BRR	Yield_Mg_ha_BLUE	ILH1_2016	8.719	4.687	7.329	1.860	0.640	1.190
E+G+BRR	Yield_Mg_ha_BLUE	INH1_2016	2.415	2.675	2.435	0.903	1.098	0.992
E+G+BRR	Yield_Mg_ha_BLUE	MIH1_2016	4.045	6.342	17.412	0.638	0.364	0.232
E+G+BRR	Yield_Mg_ha_BLUE	MNH1_2016	1.268	1.350	1.270	0.939	1.063	0.999
E+G+BRR	Yield_Mg_ha_BLUE	MOH1_2016	7.968	4.093	10.724	1.947	0.382	0.743
E+G+BRR	Yield_Mg_ha_BLUE	NCH1_2016	4.467	9.870	3.889	0.453	2.538	1.149
E+G+BRR	Yield_Mg_ha_BLUE	NEH1_2016	4.993	4.703	3.515	1.062	1.338	1.421
E+G+BRR	Yield_Mg_ha_BLUE	NYH2_2016	16.252	22.892	17.091	0.710	1.339	0.951
E+G+BRR	Yield_Mg_ha_BLUE	OHH1_2016	1.830	4.374	2.456	0.418	1.781	0.745
E+G+BRR	Yield_Mg_ha_BLUE	WIH1_2016	3.665	4.548	2.558	0.806	1.778	1.433
E+G+BRR	Yield_Mg_ha_BLUE	WIH2_2016	4.630	4.859	5.700	0.953	0.853	0.812
E+G+BRR	Yield_Mg_ha_BLUE	Across	–	–	–	1.038	1.107	0.986
E+G+BRR	Yield_Mg_ha_weight	ARH1_2016	0.989	1.219	1.311	0.811	0.930	0.755
E+G+BRR	Yield_Mg_ha_weight	DEH1_2016	0.163	0.029	0.076	5.723	0.375	2.143
E+G+BRR	Yield_Mg_ha_weight	GAH1_2016	0.035	0.251	0.134	0.138	1.870	0.259
E+G+BRR	Yield_Mg_ha_weight	IAH1_2016	3.743	3.506	3.050	1.068	1.150	1.227
E+G+BRR	Yield_Mg_ha_weight	IAH2_2016	0.175	0.372	3.081	0.471	0.121	0.057
E+G+BRR	Yield_Mg_ha_weight	IAH3_2016	0.788	0.976	2.401	0.808	0.407	0.328
E+G+BRR	Yield_Mg_ha_weight	IAH4_2016	0.498	0.065	0.179	7.678	0.362	2.782
E+G+BRR	Yield_Mg_ha_weight	ILH1_2016	1.113	0.336	0.581	3.316	0.578	1.916
E+G+BRR	Yield_Mg_ha_weight	INH1_2016	0.055	0.044	0.058	1.239	0.761	0.943
E+G+BRR	Yield_Mg_ha_weight	MIH1_2016	0.121	0.297	0.300	0.406	0.992	0.402
E+G+BRR	Yield_Mg_ha_weight	MNH1_2016	0.393	0.721	0.682	0.546	1.057	0.577
E+G+BRR	Yield_Mg_ha_weight	MOH1_2016	0.232	0.521	0.252	0.445	2.066	0.920
E+G+BRR	Yield_Mg_ha_weight	NCH1_2016	0.083	0.311	0.078	0.266	4.012	1.066
E+G+BRR	Yield_Mg_ha_weight	NEH1_2016	0.036	0.030	0.031	1.203	0.984	1.183
E+G+BRR	Yield_Mg_ha_weight	NYH2_2016	0.402	0.419	0.700	0.960	0.598	0.574
E+G+BRR	Yield_Mg_ha_weight	OHH1_2016	0.533	1.561	1.276	0.341	1.224	0.418
E+G+BRR	Yield_Mg_ha_weight	WIH1_2016	0.117	0.067	0.207	1.746	0.324	0.566
E+G+BRR	Yield_Mg_ha_weight	WIH2_2016	0.055	0.424	0.469	0.130	0.904	0.118
E+G+BRR	Yield_Mg_ha_weight	Across	–	–	–	1.516	1.040	0.902
E+G+GE+BRR	Grain_Moisture_BLUE	ARH1_2016	2.003	8.861	8.335	0.226	1.063	0.240
E+G+GE+BRR	Grain_Moisture_BLUE	DEH1_2016	5.256	4.281	9.799	1.228	0.437	0.536
E+G+GE+BRR	Grain_Moisture_BLUE	GAH1_2016	5.841	4.396	2.596	1.329	1.693	2.250
E+G+GE+BRR	Grain_Moisture_BLUE	IAH1_2016	2.857	3.613	2.833	0.791	1.275	1.008
E+G+GE+BRR	Grain_Moisture_BLUE	IAH2_2016	0.713	1.881	1.708	0.379	1.101	0.418
E+G+GE+BRR	Grain_Moisture_BLUE	IAH3_2016	2.933	3.283	2.610	0.893	1.258	1.124
E+G+GE+BRR	Grain_Moisture_BLUE	IAH4_2016	1.622	0.519	0.724	3.127	0.717	2.241
E+G+GE+BRR	Grain_Moisture_BLUE	ILH1_2016	8.071	4.964	9.657	1.626	0.514	0.836
E+G+GE+BRR	Grain_Moisture_BLUE	INH1_2016	5.315	11.384	4.258	0.467	2.674	1.248
E+G+GE+BRR	Grain_Moisture_BLUE	MIH1_2016	2.448	3.737	3.645	0.655	1.025	0.672
E+G+GE+BRR	Grain_Moisture_BLUE	MNH1_2016	13.571	4.762	6.621	2.850	0.719	2.050
E+G+GE+BRR	Grain_Moisture_BLUE	MOH1_2016	3.450	2.107	1.221	1.637	1.725	2.825
E+G+GE+BRR	Grain_Moisture_BLUE	NCH1_2016	14.869	7.756	2.226	1.917	3.485	6.681
E+G+GE+BRR	Grain_Moisture_BLUE	NEH1_2016	12.527	6.047	10.506	2.072	0.576	1.192
E+G+GE+BRR	Grain_Moisture_BLUE	NYH2_2016	9.727	4.030	5.378	2.414	0.749	1.809
E+G+GE+BRR	Grain_Moisture_BLUE	OHH1_2016	6.975	3.072	8.466	2.270	0.363	0.824
E+G+GE+BRR	Grain_Moisture_BLUE	WIH1_2016	6.024	3.495	6.151	1.723	0.568	0.979
E+G+GE+BRR	Grain_Moisture_BLUE	WIH2_2016	21.532	5.216	1.584	4.128	3.293	13.594
E+G+GE+BRR	Grain_Moisture_BLUE	Across	–	–	–	1.652	1.291	2.252
E+G+GE+BRR	Grain_Moisture_weight	ARH1_2016	14.116	33.005	48.706	0.428	0.678	0.290
E+G+GE+BRR	Grain_Moisture_weight	DEH1_2016	1.608	0.595	0.683	2.701	0.872	2.355
E+G+GE+BRR	Grain_Moisture_weight	GAH1_2016	0.862	3.261	1.258	0.264	2.593	0.685
E+G+GE+BRR	Grain_Moisture_weight	IAH1_2016	501.269	360.363	452.522	1.391	0.796	1.108
E+G+GE+BRR	Grain_Moisture_weight	IAH2_2016	43.354	1.219	28.797	35.562	0.042	1.506
E+G+GE+BRR	Grain_Moisture_weight	IAH3_2016	11.456	92.472	220.035	0.124	0.420	0.052
E+G+GE+BRR	Grain_Moisture_weight	IAH4_2016	265.697	120.354	139.962	2.208	0.860	1.898
E+G+GE+BRR	Grain_Moisture_weight	ILH1_2016	35.818	10.357	65.451	3.459	0.158	0.547
E+G+GE+BRR	Grain_Moisture_weight	INH1_2016	51.327	29.589	16.709	1.735	1.771	3.072
E+G+GE+BRR	Grain_Moisture_weight	MIH1_2016	18.430	47.158	9.360	0.391	5.039	1.969
E+G+GE+BRR	Grain_Moisture_weight	MNH1_2016	11.304	52.703	0.445	0.215	118.486	25.414
E+G+GE+BRR	Grain_Moisture_weight	MOH1_2016	3.665	5.633	128.039	0.651	0.044	0.029
E+G+GE+BRR	Grain_Moisture_weight	NCH1_2016	7.758	2.025	11.167	3.831	0.181	0.695
E+G+GE+BRR	Grain_Moisture_weight	NEH1_2016	113.669	56.705	50.862	2.005	1.115	2.235
E+G+GE+BRR	Grain_Moisture_weight	NYH2_2016	80.595	2.534	6.431	31.802	0.394	12.532
E+G+GE+BRR	Grain_Moisture_weight	OHH1_2016	12.108	4.124	14.744	2.936	0.280	0.821
E+G+GE+BRR	Grain_Moisture_weight	WIH1_2016	11.902	7.400	2.403	1.608	3.080	4.954
E+G+GE+BRR	Grain_Moisture_weight	WIH2_2016	0.917	4.113	0.764	0.223	5.385	1.201
E+G+GE+BRR	Grain_Moisture_weight	Across	–	–	–	5.085	7.900	3.409
E+G+GE+BRR	Yield_Mg_ha_BLUE	ARH1_2016	3.928	14.301	15.060	0.275	0.950	0.261
E+G+GE+BRR	Yield_Mg_ha_BLUE	DEH1_2016	5.964	3.831	3.763	1.557	1.018	1.585
E+G+GE+BRR	Yield_Mg_ha_BLUE	GAH1_2016	3.379	4.157	4.699	0.813	0.885	0.719
E+G+GE+BRR	Yield_Mg_ha_BLUE	IAH1_2016	2.287	2.820	2.767	0.811	1.019	0.826
E+G+GE+BRR	Yield_Mg_ha_BLUE	IAH2_2016	7.505	7.733	8.012	0.971	0.965	0.937
E+G+GE+BRR	Yield_Mg_ha_BLUE	IAH3_2016	7.908	5.280	4.834	1.498	1.092	1.636
E+G+GE+BRR	Yield_Mg_ha_BLUE	IAH4_2016	2.565	3.811	3.842	0.673	0.992	0.668
E+G+GE+BRR	Yield_Mg_ha_BLUE	ILH1_2016	8.036	5.919	7.366	1.358	0.804	1.091
E+G+GE+BRR	Yield_Mg_ha_BLUE	INH1_2016	6.533	1.994	2.069	3.277	0.964	3.158
E+G+GE+BRR	Yield_Mg_ha_BLUE	MIH1_2016	4.748	19.667	20.508	0.241	0.959	0.232
E+G+GE+BRR	Yield_Mg_ha_BLUE	MNH1_2016	1.422	1.265	1.248	1.124	1.014	1.140
E+G+GE+BRR	Yield_Mg_ha_BLUE	MOH1_2016	12.381	9.392	11.632	1.318	0.807	1.064
E+G+GE+BRR	Yield_Mg_ha_BLUE	NCH1_2016	5.713	3.515	3.888	1.626	0.904	1.470
E+G+GE+BRR	Yield_Mg_ha_BLUE	NEH1_2016	5.446	5.214	4.593	1.045	1.135	1.186
E+G+GE+BRR	Yield_Mg_ha_BLUE	NYH2_2016	17.271	19.504	19.128	0.886	1.020	0.903
E+G+GE+BRR	Yield_Mg_ha_BLUE	OHH1_2016	2.503	2.138	2.234	1.171	0.957	1.121
E+G+GE+BRR	Yield_Mg_ha_BLUE	WIH1_2016	2.210	3.805	2.586	0.581	1.471	0.855
E+G+GE+BRR	Yield_Mg_ha_BLUE	WIH2_2016	4.667	5.288	5.442	0.883	0.972	0.858
E+G+GE+BRR	Yield_Mg_ha_BLUE	Across	–	–	–	1.117	0.996	1.095
E+G+GE+BRR	Yield_Mg_ha_weight	ARH1_2016	2.359	0.719	1.152	3.281	0.624	2.047
E+G+GE+BRR	Yield_Mg_ha_weight	DEH1_2016	0.051	0.020	0.186	2.540	0.108	0.273
E+G+GE+BRR	Yield_Mg_ha_weight	GAH1_2016	0.026	0.387	0.568	0.068	0.682	0.046
E+G+GE+BRR	Yield_Mg_ha_weight	IAH1_2016	2.914	2.808	2.836	1.038	0.990	1.027
E+G+GE+BRR	Yield_Mg_ha_weight	IAH2_2016	0.069	0.110	0.135	0.626	0.813	0.509
E+G+GE+BRR	Yield_Mg_ha_weight	IAH3_2016	0.670	1.666	3.383	0.402	0.493	0.198
E+G+GE+BRR	Yield_Mg_ha_weight	IAH4_2016	0.199	0.058	0.113	3.423	0.516	1.766
E+G+GE+BRR	Yield_Mg_ha_weight	ILH1_2016	0.751	0.594	0.808	1.264	0.736	0.930
E+G+GE+BRR	Yield_Mg_ha_weight	INH1_2016	0.112	0.099	0.064	1.130	1.550	1.750
E+G+GE+BRR	Yield_Mg_ha_weight	MIH1_2016	0.055	0.247	0.074	0.223	3.325	0.741
E+G+GE+BRR	Yield_Mg_ha_weight	MNH1_2016	0.146	0.338	0.673	0.432	0.502	0.217
E+G+GE+BRR	Yield_Mg_ha_weight	MOH1_2016	0.283	0.522	0.082	0.542	6.396	3.466
E+G+GE+BRR	Yield_Mg_ha_weight	NCH1_2016	0.113	0.227	0.100	0.499	2.277	1.135
E+G+GE+BRR	Yield_Mg_ha_weight	NEH1_2016	0.033	0.127	0.076	0.263	1.665	0.438
E+G+GE+BRR	Yield_Mg_ha_weight	NYH2_2016	0.449	0.418	0.553	1.074	0.756	0.812
E+G+GE+BRR	Yield_Mg_ha_weight	OHH1_2016	1.202	1.483	0.850	0.811	1.745	1.414
E+G+GE+BRR	Yield_Mg_ha_weight	WIH1_2016	0.204	0.110	0.066	1.858	1.672	3.107
E+G+GE+BRR	Yield_Mg_ha_weight	WIH2_2016	0.185	0.073	1.076	2.524	0.068	0.172
E+G+GE+BRR	Yield_Mg_ha_weight	Across	–	–	–	1.222	1.384	1.114

**Table B7:** Variance components (Var_Comp) for environment (Env) Line and Genotype by environment (Env:Line) interaction for each data set. CV denotes coefficient of variation and n_Env denotes the average of number of environments in each data set.

Data	Component	VarComp	Trait	Heritability	CV	n_Env
Japonica	Env:Line	186065.908	GY	0.285	0.163	3.597
Japonica	Line	257287.998	GY	0.285	0.163	3.597
Japonica	Env	1860782.427	GY	0.285	0.163	3.597
Japonica	Residual	272836.420	GY	0.285	0.163	3.597
Japonica	Env:Line	0.000	PHR	0.462	0.073	3.597
Japonica	Line	0.000	PHR	0.462	0.073	3.597
Japonica	Env	0.001	PHR	0.462	0.073	3.597
Japonica	Residual	0.000	PHR	0.462	0.073	3.597
Japonica	Env:Line	0.000	GC	0.249	0.818	3.597
Japonica	Line	0.001	GC	0.249	0.818	3.597
Japonica	Env	0.006	GC	0.249	0.818	3.597
Japonica	Residual	0.001	GC	0.249	0.818	3.597
Japonica	Env:Line	0.002	PH	0.624	0.097	3.597
Japonica	Line	20.528	PH	0.624	0.097	3.597
Japonica	Env	35.950	PH	0.624	0.097	3.597
Japonica	Residual	8.576	PH	0.624	0.097	3.597
USP	Env:Line	0.983	GY	0.533	0.378	4
USP	Line	1.129	GY	0.533	0.378	4
USP	Env	2.123	GY	0.533	0.378	4
USP	Residual	0.850	GY	0.533	0.378	4
G2F_2014	Env:Line	0.001	Grain_Moisture_BLUE	0.609	0.196	5.376
G2F_2014	Line	3.913	Grain_Moisture_BLUE	0.609	0.196	5.376
G2F_2014	Env	11.492	Grain_Moisture_BLUE	0.609	0.196	5.376
G2F_2014	Residual	2.006	Grain_Moisture_BLUE	0.609	0.196	5.376
G2F_2014	Env:Line	1.061	Grain_Moisture_weight	0.010	1.877	5.376
G2F_2014	Line	0.344	Grain_Moisture_weight	0.010	1.877	5.376
G2F_2014	Env	175.200	Grain_Moisture_weight	0.010	1.877	5.376
G2F_2014	Residual	3.331	Grain_Moisture_weight	0.010	1.877	5.376
G2F_2014	Env:Line	0.697	Yield_Mg_ha_BLUE	0.423	0.271	5.376
G2F_2014	Line	0.822	Yield_Mg_ha_BLUE	0.423	0.271	5.376
G2F_2014	Env	4.475	Yield_Mg_ha_BLUE	0.423	0.271	5.376
G2F_2014	Residual	0.853	Yield_Mg_ha_BLUE	0.423	0.271	5.376
G2F_2014	Env:Line	0.118	Yield_Mg_ha_weight	0.461	0.576	5.376
G2F_2014	Line	0.162	Yield_Mg_ha_weight	0.461	0.576	5.376
G2F_2014	Env	0.699	Yield_Mg_ha_weight	0.461	0.576	5.376
G2F_2014	Residual	0.202	Yield_Mg_ha_weight	0.461	0.576	5.376
G2F_2015	Env:Line	0.001	Grain_Moisture_BLUE	0.603	0.160	4.217
G2F_2015	Line	2.004	Grain_Moisture_BLUE	0.603	0.160	4.217
G2F_2015	Env	3.286	Grain_Moisture_BLUE	0.603	0.160	4.217
G2F_2015	Residual	2.270	Grain_Moisture_BLUE	0.603	0.160	4.217
G2F_2015	Env:Line	0.001	Grain_Moisture_weight	0.109	1.435	4.217
G2F_2015	Line	0.655	Grain_Moisture_weight	0.109	1.435	4.217
G2F_2015	Env	19.808	Grain_Moisture_weight	0.109	1.435	4.217
G2F_2015	Residual	2.699	Grain_Moisture_weight	0.109	1.435	4.217
G2F_2015	Env:Line	1.002	Yield_Mg_ha_BLUE	0.359	0.272	4.217
G2F_2015	Line	0.633	Yield_Mg_ha_BLUE	0.359	0.272	4.217
G2F_2015	Env	2.604	Yield_Mg_ha_BLUE	0.359	0.272	4.217
G2F_2015	Residual	1.164	Yield_Mg_ha_BLUE	0.359	0.272	4.217
G2F_2015	Env:Line	0.007	Yield_Mg_ha_weight	0.361	0.660	4.217
G2F_2015	Line	0.048	Yield_Mg_ha_weight	0.361	0.660	4.217
G2F_2015	Env	0.284	Yield_Mg_ha_weight	0.361	0.660	4.217
G2F_2015	Residual	0.070	Yield_Mg_ha_weight	0.361	0.660	4.217
G2F_2016	Env:Line	0.000	Grain_Moisture_BLUE	0.830	0.142	10.055
G2F_2016	Line	2.387	Grain_Moisture_BLUE	0.830	0.142	10.055
G2F_2016	Env	3.584	Grain_Moisture_BLUE	0.830	0.142	10.055
G2F_2016	Residual	1.335	Grain_Moisture_BLUE	0.830	0.142	10.055
G2F_2016	Env:Line	0.014	Grain_Moisture_weight	0.109	1.259	10.055
G2F_2016	Line	0.468	Grain_Moisture_weight	0.109	1.259	10.055
G2F_2016	Env	34.317	Grain_Moisture_weight	0.109	1.259	10.055
G2F_2016	Residual	4.322	Grain_Moisture_weight	0.109	1.259	10.055
G2F_2016	Env:Line	1.477	Yield_Mg_ha_BLUE	0.736	0.252	10.055
G2F_2016	Line	1.337	Yield_Mg_ha_BLUE	0.736	0.252	10.055
G2F_2016	Env	2.211	Yield_Mg_ha_BLUE	0.736	0.252	10.055
G2F_2016	Residual	1.133	Yield_Mg_ha_BLUE	0.736	0.252	10.055
G2F_2016	Env:Line	0.020	Yield_Mg_ha_weight	0.341	0.598	10.055
G2F_2016	Line	0.023	Yield_Mg_ha_weight	0.341	0.598	10.055
G2F_2016	Env	0.372	Yield_Mg_ha_weight	0.341	0.598	10.055
G2F_2016	Residual	0.051	Yield_Mg_ha_weight	0.341	0.598	10.055

### Japonica dataset

#### Predictor: E+G

**Figure 1:**
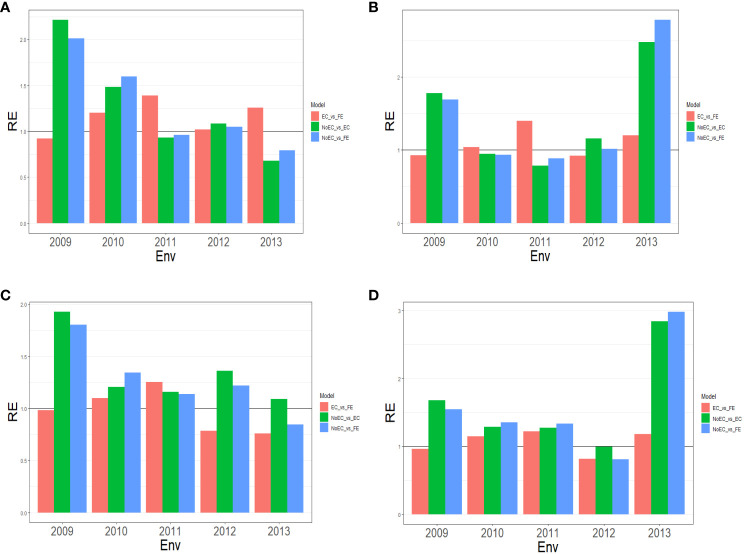
The three relative efficiencies, considering EC_vs_FE, NoEC_vs_EC, and NoEC_vs_FE, for Japonica dataset, for predictors **(A)** E+G, **(B)** E+G+GE, **(C)** E+G+BRR and **(D)** E+G+GE+BRR in terms of mean squared error (MSE) for each Environment across traits.

**Figure 1A** provides a summary of **Table B1** across traits and reveals that FE outperformed EC in most environments with improvements of 20.260% (2010), 38.920% (2011), 1.750% (2012), and 25.470% (2013). This results in an average RE of 1.1567. EC, on the other hand, outperformed NoEC in most environments with improvements of 121.200% (2009), 48.080% (2010), and 8.140% (2012), resulting in an average RE of 1.277. Likewise, FE outperformed NoEC in 101.240% (2009), 59.560% (2010), and 4.710% (2012), with slight losses in other environments, but an average RE of 1.2814. This indicates that using EC and FE surpassed NoEC by 27.730% and 28.140%, respectively. These calculations are derived from the results presented in **Table B1**.

#### Predictor: E+G+GE

**Figure 1B** summarizes the findings from **Table B1** across traits, illustrating the comparative performance of FE, EC, and NoEC techniques in various environments. The results indicate that FE outperformed EC in the majority of environments, with improvements of 4.280% (2010), 40.050% (2011), and 20.220% (2013), resulting in an average RE of 1.099. On the other hand, EC outperformed NoEC in most environments, with improvements of 78.070% (2009), 16.100% (2012), and 147.980% (2013), yielding an average RE of 1.430. Furthermore, FE surpassed the conventional NoEC technique by 68.990% (2009), 1.780% (2012), and 178.280% (2013), with an average RE of 1.462. These results indicate that using EC and FE techniques outperformed the conventional NoEC technique by 43.040% and 46.150%, respectively. The calculations are derived from the outcomes presented in **Table B1**.

#### Predictor: E+G+BRR

**Figure 1C** provides an overview of **Table B2** across traits. It reveals that FE outperformed EC only in environments 2010 (9.630%) and 2011 (25.340%), resulting in an average RE of 0.975. On the other hand, EC outperformed NoEC in all environments, with percentages of improvement of 92.640% (2009), 20.690% (2010), 15.960% (2011), 36.170% (2012), and 9.070% (2013), and an average RE of 1.349. Additionally, FE outperformed the NoEC technique in 80.390% (2009), 34.120% (2010), 13.690% (2011), and 21.950% (2012) of the environments with a slight loss in 2013, but an average RE of 1.269. These findings indicate that using EC and FE techniques surpassed NoEC in 34.910% and 26.940% of the environments, respectively. The calculations are based on the results presented in **Table B2**.

#### Predictor: E+G+GE+BRR

**Figure 1D** summarizes the findings from **Table B2** across traits. It reveals that FE displayed a superior performance over EC in environments 2010 (14.770%), 2011 (21.700%), and 2013 (17.870%), resulting in an average RE of 1.064. On the other hand, EC outperformed NoEC in most environments, namely 67.750% (2009), 28.390% (2010), 27.210% (2011), and 183.970% (2013), with an average RE of 1.614. Moreover, FE outperformed NoEC in most environments, specifically 54.260% (2009), 35.520% (2010), 33.140% (2011), and 197.980% (2013), with an average RE of 1.604. These findings indicate that using EC and FE surpassed NoEC in 61.390% and 60.460% of cases, respectively. The computations for these results were based on the findings presented in **Table B2**.

### USP dataset

#### Predictor: E+G

**Figure 2:**
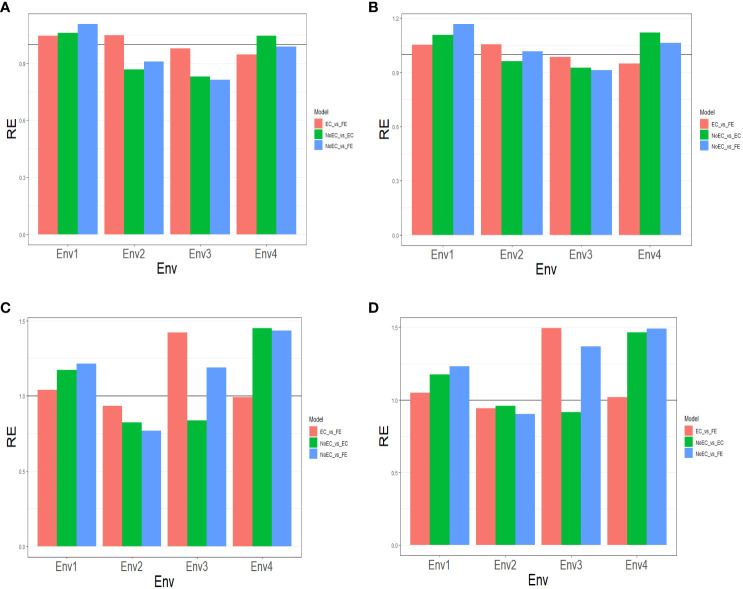
The three relative efficiencies, considering EC_vs_FE, NoEC_vs_EC, and NoEC_vs_FE, for USP dataset, for predictors **(A)** E+G, **(B)** E+G+GE, **(C)** E+G+BRR and **(D)** E+G+GE+BRR in terms of mean squared error (MSE) for each Environment.

**Figure 2A** and **Table B3** provide the results of our comparison between the NoEC and FE techniques using the RE metric. FE outperformed the NoEC technique only in Env1 (1.107), displaying an improvement of 10.670%. However, in Env2 (0.910), Env3 (0.8123), and Env4 (0.989), the NoEC technique surpassed FE, resulting in an average RE of 0.955. This average RE indicates a general loss of 4.520% when using FE compared to NoEC (see **Table B3**).

#### Predictor: E+G+GE

**Figure 2B** and **Table B3** provide the results of our comparison between the NoEC and FE techniques based on the RE metric, including the fact that the use of FE outperformed the use of NoEC in environments Env1 (1.167), Env2 (1.016), and Env4 (1.064), resulting in respective improvements of 16.670%, 1.550%, and 6.390%. However, in Env3 (0.912), the NoEC technique outperformed FE, resulting in an average RE of 1.040. This average RE indicates a general improvement of 4.000% of the FE technique regarding the NoEC method. For more detailed information, see **Table B3**.

#### Predictor: E+G+BRR

Based on **Figure 2C** and **Table B4**, our comparison between the NoEC and FE techniques using the RE metric reveals that FE outperformed the NoEC technique in environments Env1 (1.216), Env3 (1.189), and Env4 (1.435), displaying improvements of 21.580%, 18.890%, and 43.500%, respectively. However, in Env2 (0.768), the NoEC technique outperformed using FE. In general, FE outperformed NoEC by 15.200% since an average RE of 1.152 was observed (see **Table B4**).

#### Predictor: E+G+GE+BRR

Finally, based on the analysis presented in **Figure 2D** and **Table B4**, we compared the NoEC and FE techniques using the RE metric. The results indicate that FE outperformed NoEC in Env1 (1.231), Env3 (1.368), and Env4 (1.491), displaying improvements of 23.090%, 36.760%, and 49.080%, respectively. However, in Env2 (0.901), the NoEC technique outperformed FE, although, FE outperformed the NoEC in general terms, since an average RE of 1.248 was observed (see **Table B4**).

### G2F_2016 dataset

#### Predictor: E+G

**Figure 3:**
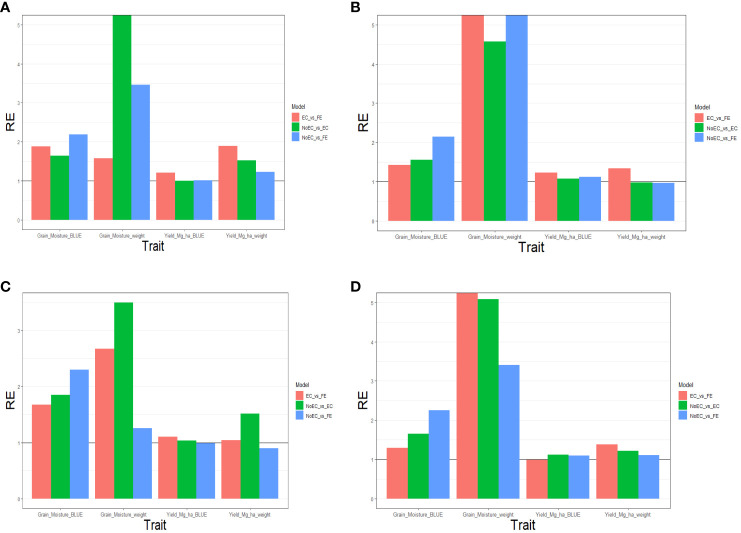
The three relative efficiencies, considering EC_vs_FE, NoEC_vs_EC, and NoEC_vs_FE, for G2F_2016 dataset, for predictors **(A)** E+G, **(B)** E+G+GE, **(C)** E+G+BRR and **(D)** E+G+GE+BRR in terms of mean squared error (MSE) for each trait across environments.

**Figure 3A** summarizes **Table B5** across different environments for each trait. It reveals that FE outperformed EC in all traits, achieving improvements of 87.970% (Grain_Moisture_BLUE), 58.100% (Grain_Moisture_weight), 21.030% (Yield_Mg_ha_BLUE), and 89.600% (Yield_Mg_ha_weight), resulting in an average RE of 1.642. In contrast, EC outperformed NoEC in most traits, with improvements of 63.960% (Grain_Moisture_BLUE), 1682.340% (Grain_Moisture_weight), and 52.860% (Yield_Mg_ha_weight), yielding an average RE of 5.497. Additionally, FE surpassed NoEC in all traits, with enhancements of 119.370% (Grain_Moisture_BLUE), 245.980% (Grain_Moisture_weight), 1.400% (Yield_Mg_ha_BLUE), and 22.630% (Yield_Mg_ha_weight), resulting in an average RE of 1.974. These findings indicate that both EC and FE techniques outperformed NoEC by 449.740% and 97.350%, respectively. The computations are based on the results presented in **Table 5B**.

#### Predictor: E+G+GE

**Figure 3B** and **Table B5** shows that for the Yield_Mg_ha_weight trait, the NoEC technique achieved the best performance in most environments, as shown by the MSE values (DEH1_2016 [0.051], GAH1_2016 [0.026], IAH1_2016 [2.914], IAH2_2016 [0.069], MIH1_2016 [0.055], MNH1_2016 [0.146], NEH1_2016 [0.033], NYH2_2016 [0.449] and OHH1_2016 [1.202]). On average, there were slight losses of 2.210% and 2.570% when comparing EC versus NoEC and FE versus NoEC, respectively. This suggests that EC and FE techniques could have performed more adequately than the conventional NoEC technique. However, comparing EC and FE techniques based on RE showed that FE outperformed EC in most environments under NoEC, resulting in an average RE of 1.339, indicating a superiority of 33.930% for FE (see **Table 5B**).

#### Predictor: E+G+BRR

**Figure 3C** summarizes the findings from **Table B6** across environments for each trait. It shows that FE outperformed EC in all characteristics, with improvements of 67.090% (Grain_Moisture_BLUE), 167.270% (Grain_Moisture_weight), 10.650% (Yield_Mg_ha_BLUE), and 3.960% (Yield_Mg_ha_weight), resulting in an average RE of 1.622. Additionally, EC outperformed NoEC in all traits, with improvements of 84.880% (Grain_Moisture_BLUE), 249.510% (Grain_Moisture_weight), 3.780% (Yield_Mg_ha_BLUE), and 51.630% (Grain_Moisture_weight), resulting in an average RE of 1.975. Furthermore, FE outperformed NoEC only in the traits Grain_Moisture_BLUE (129.850%) and Grain_Moisture_weight (25.410%), with an average RE of 1.360. These results indicate that EC and FE techniques outperformed the conventional NoEC technique in 62.240% and 36.020% of cases, respectively. These calculations are derived from the results presented in **Table B6**.

#### Predictor: E+G+GE+BRR

**Figure 3D** summarizes the results from **Table B6** across different traits. It shows that FE outperformed EC in the majority of traits, specifically by 29.090% for Grain_Moisture_BLUE, 689.960% for Grain_Moisture_weight, and 38.420% for Yield_Mg_ha_weight. This leads to an average RE of 2.893. On the other hand, EC outperformed NoEC in all traits, with improvements of 65.180% for Grain_Moisture_BLUE, 408.510% for Grain_Moisture_weight, 11.690% for Yield_Mg_ha_BLUE, and 22.200% for Yield_Mg_ha_weight. The average RE for EC compared to NoEC is 2.269. Furthermore, FE outperformed NoEC in all traits, with improvements of 125.150% for Grain_Moisture_BLUE, 240.900% for Grain_Moisture_weight, 9.490% for Yield_Mg_ha_BLUE, and 11.380% for Yield_Mg_ha_weight. The average RE for FE compared to NoEC is 1.967. These results indicate that using EC and FE outperformed NoEC by 126.890% and 96.730%, respectively. These computations are derived from the outcomes of **Table B6**.

### Summary across data sets for each predictor

In **Table 1** we can observe that in any of the four predictors using environmental covariates improve prediction accuracy at least 61.400% regarding of not using the environmental covariates (NoEC_vs_EC). Also, we can see in this same table that using FE improves the prediction performance in the four predictors regarding of using the original environmental covariates (EC_vs_FE) in at least 347.300%. Regarding using FE and not using environmental covariates (NoEC_vs_FE) we can observe that also in the four predictors using FE outperform by at least 113.100% not using the environmental covariates. Also, we observed that in many cases adding directly the environmental covariates (EC) not improve (and even reduce) the prediction performance and for this reason, we observe that the gain in terms of prediction performance of NoEC_vs_FE is less pronounced regarding comparing EC_vs_FE.

**Table 1:** Summary of relative efficiencies (RE) across data sets for each predictor.

Predictor	NoEC_vs_EC_	EC_vs_FE	NoEC_vs_FE
E+G	2.573	8.419	2.131
E+G+BRR	1.614	6.574	2.641
E+G+GE	2.489	4.473	3.141
E+G+GE+BRR	4.882	12.138	7.692
Average	2.889	7.901	3.901

NoEC_vs_EC denotes the RE of no using environmental covariates (NoEC) vs using environmental covatiates (EC), EC_vs_FE denotes the RE efficiency of comparing using EC vs using the environmental covatiates with feature engineering (FE) and NoEC_vs_FE is the RE of using FE regarding of no using environmental covariates (NoEC).

## Discussions

Due to the fact, that still the practical implementation of the GS methodology is challenging since not always is possible to guarantee high genomic-enabled prediction accuracy, many strategies had been developed to improve the machine learning genomic prediction ability (Sallam and Smith, 2016). For this reason, since the GS methodology is still not optimal, this investigation explored FE on the environmental covariates. FE is a crucial step in machine learning and data science that involves creating new features or modifying existing ones to improve the performance of a model. FE is a creative and essential aspect of the machine learning workflow, and it can significantly impact the success of one’s models. It is a skill that improves with experience and a deep understanding of the data and problem. For this reason, FE has been applied successfully in solving natural language processing, computer vision, time series and other issues.

FE is not new in the context of GS, since some studies had been conducted exploring feature engineering techniques from the feature selection point of view. For example, Long et al. (2011) used dimension reduction and variable selection for genomic selection to predict milk yield in Holsteins. Tadist et al. (2019) present a systematic and structured literature review of the feature-selection techniques used in studies related to big genomic data analytics. While Meuwissen et al. (2017) proposed variable selection models for genomic selection using whole-genome sequence data and singular value decomposition. More recently Montesinos-López et al. (2023) proposed feature selection methods for selecting environmental covariables to enhance genomic prediction accuracy. However, these studies are only focused on feature selection and not create new features from the original inputs.

From our results across traits and data sets, we can state that including environmental covariates significantly improves the prediction performance, since comparing no environmental covariates (NoEC) vs adding environmental covariates (EC), the resulting improvement was of 167.900% (RE=2.679 of NoEC_vs_EC), 142.100 (RE=2.242 of NoEC_vs_EC), 56.100% (RE=1.561 of NoEC_vs_EC) and 421.300% (RE=5.213 of NoEC_vs_EC) under predictor E+G, E+G+GE, E+G+BRR and E+G+GE+BRR respectively. However, it is very interesting to point out that the prediction performance can be even improved when the covariates are included but using FE. We found that the improvement of the prediction performance using FE only including only the EC was of 816.600% (RE=9.166 of EC_vs_FE), 372.900% (RE=4.729 of EC_vs_FE), 616.100% (RE=716.100 of EC_vs_FE) and 1240.900% (RE=13.409% of EC_vs_FE) under predictors E+G, E+G+GE, E+G+BRR and E+G+GE+BRR respectively. The larger gain in prediction performance was observed under the most complex predictor (E+G+GE+BRR), while the lowest gain was observed under predictor E+G+GE. Our results show that FE in genomic prediction holds tremendous potential for advancing our understanding of genetics and improving predictions related to various aspects of genomics. For this reason, FE should be considered an important tool to unlock the potential of genomic data for research and practical applications of genomic prediction.

Although our results are very promising for the use of FE, its practical implementation is very challenging, since we observed a significant improvement in some data sets but not in all, and for practical implementations, we need to be able to identify with a high degree of accuracy when the use of FE will be beneficial and when the use of this approach will not be successful. Also, it is important to point out that we have opted against utilizing the Pearson’s correlation coefficient as a performance metric for predicting outcomes. This decision is principally rooted in the lack of substantial improvement linked to this measure we observed. The marginal benefits observed with this metric can be partly ascribed to our exclusive focus on feature selection within the realm of environmental covariates. Additionally, this can be attributed to the assessment of environmental covariates not at the genotype level but rather at the environmental (location) level.

Three reasons why the FE works well for some data but not very well for others are: (1) that those data sets with low efficiency with FE are those in which the environmental covariates are less correlated with the response variable, (2) that we speculate that not for all data sets the type of FE we implemented are efficient and (3) FE capture complex relationships between the inputs and the response variable. These mean that the nature of each data set affects substantially the performance of any FE strategy. For these reasons some challenges for its implementation are: *a) Domain Knowledge Requirement*: Effective FE often requires a deep understanding of the domain. With domain expertise, it can be easier to identify relevant features or transformations that could enhance model performance; *b) Data Quality and Quantity*: Obtaining high-quality and sufficient data for FE can be challenging in many practical scenarios. Limited or noisy data can hinder the creation of meaningful features; *c) Time and Resource Constraints*: Implementing FE can be time-consuming, and in some real-world applications, there might be strict time and resource constraints. This makes exploring and experimenting with a wide range of FE techniques challenging; *d) Dynamic Data:* Real-world data often changes over time. Features that are effective at one point in time may become less relevant or even obsolete as the data distribution evolves. Maintaining and updating features in dynamic environments can be challenging; *e) Overfitting Risks*: Aggressive. FE can lead to overfitting, especially when the number of features is large compared to the amount of available data. Overfit models perform well on training data but generalize poorly to new, unseen data; *f) Complexity and Interpretability*: As the number and complexity of features increase, the resulting models can become difficult to interpret. This lack of interpretability can be challenging, especially in applications where understanding the model’s decisions is crucial*; g*) Automated Feature Selection: While manual FE can be effective, the process is often subjective and time-consuming. Automated feature selection methods exist, but selecting the right techniques and parameters can be challenging; *h) Curse of Dimensionality*: As the number of features increases, the curse of dimensionality becomes more pronounced. This can lead to increased computational requirements and decreased model performance, making it challenging to strike the right balance.

The results of this study demonstrate that the feature engineering strategy for incorporating environmental covariates effectively enhances genomic prediction accuracy. However, further research is warranted to refine the methodology for integrating environmental covariates into genomic prediction models, particularly in the context of modeling genotype-environment interactions (GE). For instance, employing the factor analytic (FA) multiplicative operator to describe cultivar effects in different environments has shown promise as a robust and efficient machine learning approach for analyzing multi-environment breeding trials (Piepho, 1998; Smith et al., 2005). Factor analysis offers solutions for modeling GE with heterogeneous variances and covariances, either alongside the numerical relationship matrix (based on pedigree information) (Crossa et al., 2006) or utilizing the genomic similarity matrix to assess GE (Burgueño et al., 2012). Further research is needed to comprehensively explore the application of the FA approach for feature engineering of environmental covariates within the framework of genomic prediction.

## Conclusions

This study delved into the impact of feature engineering on environmental covariates to enhance the predictive capabilities of genomic models. Our findings demonstrate a consistent improvement in prediction performance, as measured by MSE, across most datasets when employing feature engineering techniques compared to models without such enhancements. While some datasets showed no significant gains, others exhibited notably substantial improvements. These results underscore the potential of feature engineering to bolster prediction accuracy in genomic studies. However, it’s imperative to acknowledge the inherent complexity and challenges associated with practical implementation, as various factors can influence its efficacy. Therefore, we advocate for further exploration and adoption of feature engineering methodologies within the scientific community to accumulate more empirical evidence and harness its full potential in genomic prediction.

## Data availability statement

The original contributions presented in the study are included in the article/**Supplementary Material**. Further inquiries can be directed to the corresponding authors.

## Author contributions

OM: Writing – review & editing, Writing – original draft, Software, Methodology, Investigation, Conceptualization. LC: Writing – review & editing, Conceptualization. CS: Writing – review & editing, Supervision, Project administration, Investigation. BC: Writing – review & editing, Software, Methodology, Formal Analysis, Data curation, Conceptualization. GH: Writing – review & editing, Software, Conceptualization. BA: Writing – review & editing, Software, Methodology, Investigation, Data curation. SR: Writing – review & editing, Software, Methodology, Investigation. GG: Writing – review & editing, Methodology, Investigation, Data curation. KA: Writing – review & editing, Methodology, Investigation. RF: Writing – review & editing, Methodology, Investigation, Conceptualization. AM: Writing – review & editing, Software, Methodology, Investigation, Conceptualization. JC: Writing – review & editing, Writing – original draft, Investigation, Conceptualization.

## Appendix A

### Bayesian ridge regression

Bayesian Ridge Regression (BRR) is a probabilistic approach to linear regression that incorporates Bayesian principles. It is a regularized regression method that extends traditional linear regression by introducing a prior distribution over the regression coefficients. This approach provides a way to express uncertainty in the model parameters and helps prevent overfitting by introducing regularization.

The model assumptions assumes a traditional linear regression, with a linear relationship between the independent variables and the dependent variable. The BRR assumes that the coefficients of the regression model follow a Gaussian (normal) distribution. This introduces a regularization term that penalizes large coefficients, helping to prevent overfitting.

The model formulation assumes that ***X*** is an independent variables with and a dependent variable y, such that the BRR can be written as

y=Xβ+ϵ

where *y* is the dependent variable. ***X*** is the matrix of independent variables, ***β*** is the vector of regression coefficients and ϵ is the residual (error) term. From a Bayesian perspective, the prior distribution for *β* is assumed to be Gaussian (normal) $\beta ∼N(0,\alpha^{-1}I)$ with α being a hyperparameter controlling the strength of the regularization and I is the identity matrix. The goal is to estimate the posterior distribution of β given the data. The posterior distribution is proportional to the product of the likelihood and the prior P(β∣X,y)∝P(y∣X,β)·P(β) . Once the posterior distribution is obtained, Bayesian inference can be performed with. point estimates (mean or mode) of the posterior distribution can be used as the regression coefficients. additionally, credible intervals can be computed to quantify uncertainty.

## Appendix B

### Japonica dataset

#### Predictor: E+G

**Table B1** shows an adequate performance for the results under NoEC for the GC trait across all environments. The MSE values for 2009, 2010, 2011, 2012, and 2013 were 0.0035, 0.0110, 0.0019, 0.0281, and 0.0017, respectively. Comparing the NoEC results to the EC and FE techniques using Relative Efficiency (RE), all RE values were below 1. On average, NoEC presented 50.050% better performance compared to EC and 42.230% better performance compared to FE. However, when comparing EC and FE techniques based on RE, FE outperformed EC in 2010, 2011, 2012, and 2013, with RE values of 1.287, 2.686, 1.139, and 1.586, respectively. In 2009, EC had a lower RE value of 0.522. On average, the use of FE outperformed EC by 44.410%. Please refer to **Table B1** for more detailed information.

Concerning the GY trait, **Table B1** shows that the use of EC led to a superior performance in most environments based on MSE (796,963 [2009], 2,488,872 [2010] and 1,157,280 [2012]). However, the exceptions occurred in 2011 and 2013, when FE achieved the best MSE values of 2,615,758 and 377,719, respectively. By contrast, when comparing NoEC versus EC and NoEC versus FE using RE, most RE values were greater than 1. On average, the EC technique displayed an improvement of 105.610% (NoEC_vs_EC) regarding the NoEC method, and an improvement of 77.570% (NoEC_vs_FE) was observed with the use of FE compared to the conventional NoEC technique. Nonetheless, when assessing the performance of EC and FE techniques based on RE, FE only outperformed EC in 2011 (RE = 1.091) and 2013 (RE = 1.087). EC, on the other hand, outperformed FE in 2009 (RE = 0.777), 2010 (RE = 0.817), and 2012 (RE = 0.806), resulting in an average RE of 0.916. This indicates an overall performance loss of 8.450% when using FE compared to EC. **Table B1** provides further details.

In terms MSE for the PH trait, **Table B1** shows that the use of FE achieved the best performance in most environments (15.872 [2009], 10.959 [2010], and 164.039 [2012]). However, there were exceptions in 2011 and 2013, where the best MSE values were 28.573 (EC) and 18.363 (NoEC), respectively. On the other hand, when comparing NoEC versus EC and NoEC versus FE techniques using RE, most RE values were greater than 1. On average, the use of EC and FE displayed improvements of 61.570% and 70.210%, respectively, compared to the use of NoEC. Furthermore, when comparing the performance of EC and FE techniques based on RE, FE outperformed EC in all environments, resulting in an average RE of 1.0389. This indicates that using FE surpassed EC by 3.88% (**Table B1**).

In terms of MSE for the PHR trait, **Table B1** indicates that the use of FE yielded the best performance in most environments (0.001 [2009], 0.001 [2010], and 0.001[2013]). However, exceptions were found in 2011 and 2012, when the best MSE values were 0.001 (EC) and 0.006 (NoEC), respectively. On the other hand, when comparing EC versus FE and NoEC versus FE techniques using Relative Efficiency (RE), most RE values were at least 1. On average, the use of FE displayed a general improvement of 22.790%, compared to EC and 7.020% compared to the conventional NoEC technique. However, evaluating the performance of EC versus NoEC techniques based on RE showed that NoEC outperformed EC in most environments, resulting in an average RE of 0.938. This indicates a general accuracy loss of 6.200% when using EC compared to the conventional NoEC technique (**Table B1**).

#### Predictor: E+G+GE

**Table B1** shows that, in most environments, the conventional NoEC technique yielded the best performance for the GC trait, with MSE values of 0.001 (2009), 0.013 (2010), and 0.002 (2011). The exceptions occurred in 2012 and 2013, with the best MSE values of 0.025 (EC) and 0.0023 (FE). The average RE for the comparison of NoEC versus EC and NoEC versus FE techniques across environments was 0.919 and 0.9023, respectively, indicating general losses of 8.080% and 9.740% for EC and FE compared to the conventional NoEC.

Regarding the GY trait, MSE values from **Table B1** reveal that the use of EC achieved the best performance in most environments (1152261.030 [2009], 3653811.510 [2010], and 989127.170 [2012]). However, exceptions were observed in 2011 and 2013, where the best MSE values were 1834248.25 (NoEC) and 30980.32 (FE), respectively. On the other hand, when comparing NoEC versus EC and NoEC versus FE techniques using RE, most RE values were greater than 1. The average RE for NoEC versus EC and NoEC versus FE was 2.219 and 2.075, respectively, indicating general improvements of 121.860% and 107.520% compared to the use of NoEC. However, an evaluation of the performance of EC and FE techniques based on RE showed that FE outperformed EC only in 2011 (1.0267) and 2013 (1.122), while EC outperformed FE in 2009 (0.789), 2010 (0.849), and 2012 (0.7278). Consequently, the average RE for EC versus FE was 0.9029, implying a general loss of 9.710% when using FE compared to EC (**Table B1**).

Concerning the PH trait, the analysis of MSE values from **Table B1** reveals that the use of FE yielded the best performance in most environments (17.631 [2009] and 23.544 [2012]). However, exceptions were observed in 2010, 2011, and 2013, where the best MSE values were 12.954 (EC), 44.689 (NoEC), and 164.891 (NoEC), respectively. On the other hand, comparing NoEC versus EC and NoEC versus FE techniques using RE showed that most RE values were greater than 1. The average RE for NoEC versus EC and NoEC versus FE was 1.618 and 1.700, respectively, indicating general improvements of 61.810% and 70.000% compared to the conventional NoEC technique. Furthermore, when evaluating the performance of EC and FE techniques based on RE, FE consistently outperformed EC in most environments. The average RE for EC versus FE was 1.047, indicating a 4.710% advantage in favor of FE (**Table B1**).

Moreover, in the case of the PHR trait, the analysis of MSE values from **Table B1** shows that the use of FE yielded the best performance in most environments (0.001 [2009], 0.002 [2010], and 0.001 [2013]). However, there were exceptions in 2011 and 2012, where the best MSE values were 0.001 (EC) and 0.005 (NoEC), respectively. Furthermore, when comparing the RE values between NoEC versus EC and NoEC versus FE techniques, the average RE values of 0.966 and 1.168 indicate a slight loss of 3.440% and an improvement of 16.800%, respectively, for the use of EC and FE compared to the conventional NoEC technique. Nevertheless, when evaluating the performance of FE versus EC techniques based on RE, FE consistently outperformed EC in most environments. The average RE for FE versus EC was 1.282, indicating a significant improvement of 28.240% in accuracy for using FE compared to (**Table B1**).

#### Predictor: E+G+BRR

According to **Table B2**, the GC trait displayed superior performances with the conventional NoEC technique in most environments, yielding MSE values of 0.004 (2009), 0.002 (2011), and 0.0012 (2013). However, exceptions were found in 2010 and 2012, where FE achieved the best MSE values of 0.0680 and 0.009, respectively. Comparing the RE values between NoEC versus EC and NoEC versus FE techniques showed that most RE values were below 1. Nonetheless, the average RE of 1.104 (NoEC_vs_EC) and 1.189 (NoEC_vs_FE) indicated that EC and FE outperformed the conventional NoEC technique by 10.360% and 18.930%, respectively. Furthermore, when evaluating the performance of EC and FE techniques based on RE, FE presented the best performance in 2009 (1.151), 2010 (1.353), 2011 (2.044), and 2012 (1.0623), while EC outperformed FE in 2013 (0.529). Overall, the average RE 1.228 indicated that FE outperformed EC by 22.800% (**Table B2**).

Regarding the GY trait, **Table B2** indicates that the conventional NoEC technique displayed superior performances in most environments, with MSE values of 5,683,515.750 (2010), 2,749,626.080 (2012), and 405,886.860 (2013). However, exceptions were observed in 2009 and 2011, where FE achieved the best MSE values of 3,049,246.320 and 4,024,422.450, respectively. When comparing the RE values between NoEC_vs_EC and NoEC_vs_FE techniques, most values were below 1. Nevertheless, the average RE of 1.124 (NoEC_vs_EC) and 0.896 (NoEC_vs_FE) indicated an overall improvement of 12.430% for EC and a general loss of 10.450% for FE compared to the conventional NoEC technique. However, when comparing the performance of EC and FE techniques based on RE, only FE presented a superior performance in 2010 (1.029), resulting in an average RE of 0.797, which indicates a general loss of 20.350% for FE compared to EC (**Table B2**).

For the PH trait, **Table B2** shows that FE yielded the best performance in environments 2009 (15.281) and 2012 (159.312), while EC led to superior performances in environments 2010 (22.962) and 2013 (10.981). Most notably, when comparing the RE values for NoEC_vs_EC and NoEC_vs_FE, values exceeding 1 were observed. The average RE values of 1.634 (NoEC_vs_EC) and 1.5434 (NoEC_vs_FE) indicated substantial improvements of 63.350% and 54.350% respectively for using EC and FE, compared to the conventional NoEC technique. However, in evaluating the performance of EC and FE based on RE, FE exhibited a superior performance in most environments, but still resulting in an average RE of 0.954. This suggests that EC marginally outperformed FE by 4.650%. For further details, see **Table B2**.

Additionally, for the PHR trait, using FE displayed a superior performance in most environments, as indicated in **Table B2**. The best MSE values were observed in 2009 (0.001), 2010 (0.001), and 2013 (0.001). However, exceptions were noted in 2011 and 2012, where the use of EC and NoEC resulted in the best MSE values of 8e-04 and 0.0055, respectively. Furthermore, most RE values comparing NoEC_vs_EC and NoEC_vs_FE techniques were greater than 1. The average RE values of 1.535 (NoEC_vs_EC) and 1.449 (NoEC_vs_FE) indicate significant improvements of 53.530% and 44.930% respectively, compared to the conventional NoEC technique. However, when comparing the performance of the EC versus the FE techniques, the RE values were lower than 1 in most environments, resulting in an average RE of 0.9212. This suggests a general accuracy loss of 7.820% in for using FE compared to using the EC technique (**Table B2**).

#### Predictor: E+G+GE+BRR

According to **Table B2**, the GC trait displayed superior performances with the conventional NoEC technique in most environments, yielding MSE values of 0.004 (2009), 0.002 (2011), and 0.0012 (2013). However, exceptions were found in 2010 and 2012, where FE achieved the best MSE values of 0.0680 and 0.009, respectively. Comparing the RE values between NoEC versus EC and NoEC versus FE techniques showed that most RE values were below 1. Nonetheless, the average RE of 1.104 (NoEC_vs_EC) and 1.189 (NoEC_vs_FE) indicated that EC and FE outperformed the conventional NoEC technique by 10.360% and 18.930%, respectively. Furthermore, when evaluating the performance of EC and FE techniques based on RE, FE presented the best performance in 2009 (1.151), 2010 (1.353), 2011 (2.044), and 2012 (1.0623), while EC outperformed FE in 2013 (0.529). Overall, the average RE 1.228 indicated that FE outperformed EC by 22.800% (**Table B2**).

Regarding the GY trait, the analysis in **Table B2** reveals that the use of EC yielded superior results in most environments (2009 [1333530.864], 2012 [1690390.524], and 2013 [584945.854]). However, exceptions were observed in 2010 and 2011, where the NoEC approach resulted in the best MSE values of 4339466.437 and 1834248.259, respectively. Moreover, most RE values for the comparison of NoEC_vs_EC and NoEC_vs_FE techniques were greater than 1. The average RE values of 1.570 (NoEC_vs_EC) and 1.198 (NoEC_vs_FE) indicate general improvements of 57.030% and 19.790% for the use of EC and FE, respectively, compared to the use of NoEC. However, when comparing the performance of EC and FE techniques based on RE, the FE technique did not outperform EC only in 2010, resulting in an average RE of 0.773. This suggests a general loss of 22.670% accuracy for using FE compared to EC.

Regarding the PH trait, **Table B2** shows that the use of FE achieved the best performance in environments 2009 (17.332) and 2011 (22.026), while the use of EC achieved the best performance in environments 2010 (14.9561) and 2013 (11.071). Similarly, most of the RE values for the comparison of NoEC_vs_EC and NoEC_vs_FE techniques were greater than 1. The average RE values of 2.5259 (NoEC_vs_EC) and 2.362 (NoEC_vs_FE) indicate general improvements of 152.590% and 136.210% for using EC and FE, respectively, compared to the conventional NoEC technique. However, when comparing the performance of EC and FE techniques based on RE, EC outperformed FE in most environments, resulting in an average RE of 0.909. This indicates that using EC achieved a 9.100% improvement compared to using FE. For more detailed information, refer to Table 2.

**Table B2** displays that using EC yielded the best performance for the PHR trait in most environments, as indicated by the MSE. Specifically, the MSE values were as follows: 2009 (0.001), 2010 (0.001), 2011 (0.001), and 2013 (0.001). However, in 2012, the best MSE values were 0.005, achieved using both EC and NoEC. Comparing NoEC_vs_EC and NoEC_vs_FE techniques, most RE values were at least 1, with average improvements of 60.350% and 48.570% when using EC and FE, respectively, compared to NoEC. Conversely, when comparing EC versus FE techniques, most environments resulted in an average RE of 0.877, indicating a 12.260% decrease in accuracy when using FE compared to EC (**Table B2**).

### USP dataset

#### Predictor: E+G

Upon examining **Table B3**, it becomes apparent that the conventional NoEC technique achieved the best performance in terms of MSE in environments Env2 (4.073) and Env3 (5.246). However, exceptions were found in Env1 and Env4, where the optimal MSE values were 3.141 (FE) and 7.814 (EC), respectively. For further detail, refer to **Table B3**.

**Table B3** present our comparison results between the NoEC and EC techniques, assessed through the RE metric. The EC technique displayed its best performance in environments Env1 (1.059) and Env4 (1.046), showcasing improvements of 5.920% and 4.610% over the NoEC technique, respectively. However, NoEC outperformed EC in environments Env2 (0.869) and Env3 (0.831), resulting in an average RE of 0.951. This average RE indicates a general loss of 4.890% in accuracy when using EC compared to NoEC (see **Table B3**).

In terms MSE for the PH trait, **Table B1** shows that the use of FE achieved the best performance in most environments (15.872 [2009], 10.959 [2010], and 164.039 [2012]). However, there were exceptions in 2011 and 2013, where the best MSE values were 28.573 (EC) and 18.363 (NoEC), respectively. On the other hand, when comparing NoEC versus EC and NoEC versus FE techniques using RE, most RE values were greater than 1. On average, the use of EC and FE displayed improvements of 61.570% and 70.210%, respectively, compared to the use of NoEC. Furthermore, when comparing the performance of EC and FE techniques based on RE, FE outperformed EC in all environments, resulting in an average RE of 1.0389. This indicates that using FE surpassed EC by 3.88% (**Table B1**).

The EC and FE techniques were compared, using the RE metric to assess their performance. The findings indicate that the FE technique achieved its best performance in environments Env1 (1.045) and Env2 (1.048), displaying improvements of 4.480% and 4.790% over EC. However, EC exhibited a slightly better performance in environments Env3 (0.979) and Env4 (0.946), resulting in an average RE of 1.004. This average RE suggests a modest improvement of 0.430% when using FE compared to EC (see **Table B3**).

#### Predictor: E+G+GE

**Table B3** reveals the performance of the FE technique in terms of MSE across different environments. The FE technique achieved its best performance in environments Env1 (2.789) and Env2 (4.636), although exceptions were found in Env3 and Env4, where the optimal MSE values were 5.833 (NoEC) and 7.792 (EC), respectively (see **Table 3**).

**Table B3** present our comparison results between the NoEC and EC techniques, based on the RE metric. The EC technique displayed its best performance in environments Env1 (1.107) and Env4 (1.120), showing improvements of 10.72% and 12.040% over the NoEC technique. However, the NoEC technique outperformed EC in environments Env2 (0.961) and Env3 (0.925), resulting in an average RE of 1.028. This average RE indicates a general improvement of 2.840% of the EC method regarding the NoEC technique (see **Table B3**).

The EC and FE techniques were compared, using the RE metric to assess their performance. The findings indicate that the FE technique achieved its best performance in environments Env1 (1.054) and Env2 (1.057), displaying improvements of 5.380% and 5.650% over EC. However, using EC exhibited a better performance in environments Env3 (0.986) and Env4 (0.949), resulting in an average RE of 1.012. This average RE indicates a 1.150% improvement of the FE technique over EC (see **Table B3**).

#### Predictor: E+G+BRR

**Table B4** presents the results of our analysis regarding the MSE about the FE technique. The FE technique performed best in Env1 (2.859) and Env3 (4.413) environments. However, exceptions were observed in Env2 and Env4, where the optimal MSE values were 4.073 (NoEC) and 5.638 (EC), respectively. For further details, see **Table B4**.

The results of our comparison between the NoEC and EC techniques, based on the RE metric, are presented in **Table B4**. The EC technique exhibited its best performance in environments Env1 (1.171) and Env4 (1.450), suggesting improvements of 17.1000% and 45.000%, respectively, compared to the NoEC technique. However, the NoEC technique outperformed EC in environments Env2 (0.823) and Env3 (0.836), resulting in an average RE of 1.070. This average RE indicates a general improvement of 7.000% of the EC regarding the NoEC technique (see **Table B4**).

We compared the EC and FE techniques, evaluating their performance with the RE metric. The findings indicate that the FE technique achieved its best performance in environments Env1 (1.038) and Env3 (1.423), displaying respective improvements of 3.840% and 42.290% over EC. However, EC performed better in environments Env2 (0.934) and Env4 (0.990), resulting in an average RE of 1.096. This average RE indicates a 9.600% better performance of the FE technique over EC (see **Table B4**).

#### Predictor: E+G+GE+BRR

**Table B4** presents the performance results of the FE technique in terms of MSE. The best performance was observed in environments Env1 (2.644), Env3 (4.265), and Env4 (5.856). The only exception was Env2, where the optimal MSE value was 4.708, achieved using NoEC. For further information, see **Table B4**.

Based on the RE metric, the results of our comparison between the NoEC and EC techniques are presented in **Table B4**. EC performed best in environments Env1 (1.175) and Env4 (1.465), with improvements of 17.510% and 46.530%, respectively, compared to the NoEC technique. However, the NoEC technique outperformed EC in environments Env2 (0.958) and Env3 (0.915), resulting in an average RE of 1.128. This average RE indicates a general improvement of 12.830% of EC regarding NoEC. For more specific information, see **Table B4**.

We compared the EC and FE techniques based on the RE metric. The analysis revealed that the FE technique displayed its best performance in Env1 (1.047), Env3 (1.494), and Env4 (1.017). These results indicate improvements of 4.740%, 49.430%, and 1.740%, respectively, when compared to using EC. However, EC displayed a better performance in Env2 (0.941), but in general, the FE technique outperformed EC by 12.500%, since an average RE of 1.125 was observed (see **Table B4**).

### G2F_2016 dataset

#### Predictor: E+G

**Table B5** illustrates that FE yielded the best performance for the Grain_Moisture_BLUE trait in most environments. MSE values were 4.645 (DEH1_2016), 2.154 (GAH1_2016), 2.703 (IAH1_2016), 0.467 (IAH4_2016), 0.668 (MOH1_2016), 3.598 (NCH1_2016), 2.092 (NYH2_2016), and 1.601 (WIH2_2016). The average RE values showed that FE outperformed EC and NoEC by 87.970% and 119.370%, respectively. Additionally, EC displayed an average RE improvement of 63.960% over NoEC. For further detail, see **Table B5**.

For the Grain_Moisture_weight trait, EC presented the best performance based on MSE values in several environments listed in **Table 5** (ARH1_2016 [24.235], DEH1_2016 [0.207], IAH1_2016 [2.568], ILH1_2016 [2.172], INH1_2016 [0.210], MOH1_2016 [7.450], OHH1_2016 [0.454] and WIH2_2016 [0.194]). The average RE values revealed that EC and FE outperformed the conventional NoEC technique by 1682.340% and 245.980%, respectively. Furthermore, FE displayed a 58.100% improvement over EC (See **Table B5**).

Regarding the Yield_Mg_ha_BLUE trait, NoEC displayed a superior performance in most environments based on MSE values listed in **Table B5** (GAH1_2016 [3.579], IAH4_2016 [2.576], MIH1_2016 [4.045], MNH1_2016 [1.268], NYH2_2016 [16.252], OHH1_2016 [1.830] and WIH1_2016 (3.665]). The average RE values indicated that FE resulted in general improvements of 21.030% and 1.400% over EC and NoEC, respectively. However, a comparison between NoEC and EC showed a slight decrease of 0.190% in average RE for EC (see **Table B5**).

For the Yield_Mg_ha_weight trait, NoEC showed the best performance based on MSE values in most environments (DEH1_2016 [0.078], IAH4_2016 [0.091], ILH1_2016 [0.351], MIH1_2016 [0.1156], MNH1_2016 [0.391], NYH2_2016 [0.087], WIH1_2016 [0.063] and WIH2_2016 [0.019]). The average RE values indicated general improvements of 52.860% and 22.630% for EC and FE, respectively, compared to NoEC. Moreover, on average, FE outperformed EC by 89.600% (see **Table B5**).

#### Predictor: E+G+GE

**Table B5** shows that FE yielded the best performance for the Grain_Moisture_BLUE trait in the majority of environments, with MSE values ranging from 0.519 to 5.813 (IAH4_2016, ILH1_2016, MNH1_2016, NEH1_2016, NYH2_2016, OHH1_2016 and WIH1_2016). Comparing RE values, using FE outperformed EC and NoEC techniques by 42.480% and 114.740%, respectively. Additionally, EC outperformed NoEC with an average RE of 1.552, indicating a superiority of 55.210% for EC. For further details, see **Table B5**.

For the Grain_Moisture_weight trait, **Table B5** reveals that FE displayed a better performance in most environments, as indicated by the MSE values (DEH1_2016 [0.132], IAH3_2016 [0.418], IAH4_2016 [139.446], MIH1_2016 [1.668], MNH1_2016 [1.316], NCH1_2016 [6.953], NYH2_2016 [5.565], OHH1_2016 [0.195] and WIH1_2016 [1.508]). Moreover, the average RE values showed that FE outperformed EC and NoEC by 831.910% and 825.260%, respectively. Comparing NoEC and EC techniques, there was a general improvement of 357.000% for EC over NoEC, with an average RE of 4.570 (see **Table B5**).

Regarding the Yield_Mg_ha_BLUE trait, **Table B5** shows that the use of NoEC achieved the best performance in most environments, as indicated by the MSE values (GAH1_2016 [3.379], IAH1_2016 [2.287], IAH2_2016 [7.505], IAH4_2016 [3.565], MIH1_2016 [4.748], NYH2_2016 [17.271], WIH1_2016 [2.210] and WIH2_2016 [4.667]). However, most RE values comparing NoEC_vs_EC and NoEC_vs_FENoEC_vs_FEtechniques were greater than 1. On average, EC displayed a 7.450% improvement and FE showed an 11.690% improvement compared to the conventional NoEC technique. Furthermore, comparing EC and FE techniques, an average RE of 1.227 was observed, indicating that FE outperformed NoEC by 22.700% (see **Table B5**).

In terms of the Yield_Mg_ha_weight trait, **Table B5** shows that the use of NoEC achieved the best performance in most environments, as evident from the MSE values (DEH1_2016 [0.051], GAH1_2016 [0.026], IAH1_2016 [2.914], IAH2_2016 [0.0689], MIH1_2016 [0.055], MNH1_2016 [0.146], NEH1_2016 [0.033], NYH2_2016 [0.449] and OHH1_2016 [1.202]). The average RE values indicated slight losses of 2.210% and 2.570% when comparing EC versus NoEC and FE versus NoEC, respectively. This implies that EC and FE techniques did not perform as adequately as the conventional NoEC technique. However, comparing EC and FE techniques based on RE showed that FE outperformed EC in most environments, resulting in an average RE of 1.339, indicating a 33.930% superiority of FE over EC. For more detailed information, see **Table B5**.

#### Predictor: E+G+BRR

In **Table B6**, it is evident that for the Grain_Moisture_BLUE trait, the use of FE provided the best performance in most environments, as indicated by the MSE values (DEH1_2016 [4.376], GAH1_2016 [2.002], IAH1_2016 [2.036], IAH3_2016 [1.237], IAH4_2016 [0.496], MNH1_2016 [3.685], MOH1_2016 [0.678], NCH1_2016 [3.499], NYH2_2016 [2.213] and WIH2_2016 [1.648]). On average, the RE values indicate that FE outperformed EC and NoEC by 67.090% and 129.850%, respectively. Additionally, comparing NoEC and EC techniques showed that EC outperformed NoEC by an average of 84.880%. For further information, see **Table B6**.

For the Grain_Moisture_weight trait, **Table B6** shows that the use of NoEC provided the best performance in most environments, as indicated by the MSE values (GAH1_2016 [1.272], IAH1_2016 [401.574], IAH3_2016 [0.199], ILH1_2016 [5.447], MIH1_2016 [0.715[, NCH1_2016 [1.174] and NEH1_2016 [42.758]). On average, the RE values indicate that FE outperformed EC and NoEC by 167.270% and 25.410%, respectively. Furthermore, comparing NoEC and EC shows that EC outperformed NoEC with an average RE of 3.495, representing a general improvement of 149.510%. For more detailed information, see **Table B6**.

**Table B6**, for the Yield_Mg_ha_BLUE trait, shows that the use of NoEC led to the best performance in most environments, as indicated by the MSE values (ARH1_2016 [3.713], GAH1_2016 [3.579], IAH4_2016 [2.576], INH1_2016 [2016], MIH1_2016 [4.045], MNH1_2016 [1.268], NYH2_2016 [16.252], OHH1_2016 [1.829] and WIH2_2016 [4.629]). On average, the RE values indicate general improvements of 10.650% for FE compared to EC, and 3.780% for EC compared to NoEC. However, when comparing the performance of NoEC and FE techniques, an average RE of 0.986 indicates a slight loss for FE compared to NoEC. For more detailed information, see **Table B6**.

For the Yield_Mg_ha_weight trait, the use of NoEC achieved the best performance in most environments, as indicated by the MSE values (ARH1_2016 [0.989], GAH1_3016 [0.035], IAH2_2016 [0.175], IAH3_2016 [0.783], MIH1_2016 [0.1201], MHH1_2016 [0.393], MOH1_2016 [0.232], NYH2_2016 [0.402], OHH1_2016 [0.533] and WIH2_2016 [0.055]). On average, the RE values indicate a general improvement of 51.630% for EC compared to NoEC and 3.960% for FE compared to EC. However, when comparing the performance of NoEC and FE based on RE, the best performance was displayed by NoEC in most environments, resulting in an average RE of 0.9012, indicating that NoEC outperformed FE by 9.820%. For more detailed information, see **Table B6**.

#### Predictor: E+G+GE+BRR

**Table B6** shows that EC yielded the most favorable results for the Grain_Moisture_BLUE trait in various environments. The corresponding MSE values for EC were 4.2801 (DEH1_2016), 0.519 (IAH4_2016), 4.964 (ILH1_2016), 4.762 (MNH1_2016), 6.047 (NEH1_2016), 4.030 (NYH2_2016), 3.072 (OHH1_2016), and 3.495 (WIH1_2016). Additionally, the average RE values indicated that using FE outperformed both EC and NoEC by 29.090% and 125.150%, respectively (1.291 for EC_vs_FE, and 2.252 for NoEC_vs_FE). Furthermore, when comparing the NoEC and EC techniques, an average RE of 1.6512 displays the superior performance of EC over NoEC by 65.180%. For more comprehensive information, see **Table B6**.

When considering the Grain_Moisture_weight trait, the use of EC presented amor adequate performance in most environments based on the MSE values provided in Table 6 (DEH1_2016 [0.595], IAH1_2016 [360.363], IAH2_2016 [1.219], IAH4_2016 [120.354], ILH1_2016 [10.357], NCH1_2016 [2.0245], NYH2_2016 [2.534], and OHH1_2016 [4.124]). Moreover, the average RE values reveal that EC and FE outperformed the conventional NoEC by 408.510% and 240.900% respectively (5.085 for NoEC_vs_EC and 3.409 for NoEC_vs_FE). Furthermore, a comparison between EC and FE techniques indicates that an average RE of 7.899 suggests that FE outperformed EC by 689.960%. For more detailed information, see **Table B6**.

When examining the Yield_Mg_ha_BLUE trait, the use of NoEC displayed the best performance in most environments based on the MSE values presented in **Table B6** (ARH1_2016 [3.928], GAH1_2016 [3.379], IAH1_2016 [2.287], IAH2_2016 [7.505], IAH4_2016 [2.565], MIH1_2016 [4.748], NYH2_2016 [17.271], WIH1_2016 [2.210] and WIH2_2016 [4.667]). However, it is worth noting that EC and FE outperformed the conventional NoEC by 11.690% and 9.490% in terms of average RE values (1.117 for NoEC_vs_EC and 1.095 for NoEC_vs_FE). Nevertheless, when comparing FE versus EC techniques, a slight loss of 0.400% was observed for using FE compared to EC, as indicated by an average RE of 0.996. For more detailed information, see **Table B6**.

Regarding the Yield_Mg_ha_weight trait, **Table B6** shows that the use of EC yielded the best performance in most environments, as evidenced by the following MSE values: ARH1_2016 (0.719), DEH1_2016 (0.020), IAH1_2016 (2.808), IAH4_2016 (0.058), ILH1_2016 (0.594), NYH2_2016 (0.418), and WIH2_2016 (0.073). The average RE values indicated improvements of 22.200% (NoEC_vs_EC) and 11.380% (NoEC_vs_FE), highlighting the superior performance of EC and FE over the conventional NoEC technique. Conversely, when comparing EC and FE techniques, most environments performed better with an average RE of 1.384, indicating that FE outperformed EC by 38.420%. For additional information, see **Table B6**.
