# Supplementary material for: Feature engineering of environmental covariates improves plant genomic-enabled prediction
Source: Front Plant Sci. 2024 May 15;15:1349569. doi: 10.3389/fpls.2024.1349569 (PMC11135473; doi:10.3389/fpls.2024.1349569)
Supplement: Supplementary file 1 [file DataSheet_1.docx]

**SUPPLEMENTARY MATERIALS**

### G2F_2014 dataset

### *Predictor: E+G*

Based on the information presented in **Table S1**, the use of EC yielded the best performance for the Grain_Moisture_BLUE trait in most environments. Specifically, the MSE values were as follows: 4.565 (IAH1ab_2014), 20.117 (MNH1_2014), 9.639 (MOH2_2014), 1.873 (NCH1_2014), 6.253 (NEH1_2014), 4.248 (NEH3_2014), 2.657 (NYH1_2014), and 5.314 (WIH1_2014). The average RE values were calculated to be 2.939 (NoEC vs EC) and 2.537 (NoEC vs FE), indicating that the use of EC and FE outperformed NoEC by 193.930% and 153.690%, respectively. Furthermore, when comparing the performance of the EC and FE techniques based on RE, an average RE of 1.872 was observed. This indicates that the use of FE surpassed the use of EC by 87.240%. For more comprehensive details, refer to **Table S1.**

**Table S1** shows that the use of FE led to the most favorable results for the Grain_Moisture_weight trait across various environments, as indicated by the MSE. Specifically, environments IAH2_2014 (7.869), ILH1_2014 (3.328), INH1_2014 (6.828), MNH1_2014 (1.566), MOH2_2014 (2618.855), NEH1_2014 (8.960), NEH2_2014 (7.572), NEH3_2014 (34.173), NYH1_2014 (3.332), TXH2_2014 (0.927), and WIH1_2014 (5.858) displayed a superior performance with FE. Additionally, FE outperformed EC and NoEC by 114.639% and 396.180%, respectively, as indicated by the average RE values of 115.639 (EC vs FE) and 4.962 (NoEC vs FE). However, a comparison between NoEC and EC revealed an average RE of 0.700, indicating a general loss of 30.000% with EC compared to NoEC. For further information, see **Table S1**.

In addition, **Table S1** displays the results for the Yield_Mg_ha_BLUE trait, highlighting the MSE values. The use of EC demonstrated superior performance in most environments IAH2_2014 ([1.899], ILH1_2014 [4.42], INH1_2014 [1.722], MNH1_2014 [3.603], NEH1_2014 [3.920], NEH2_2014 [2.758], NEH3_2014 [1.867] and WIH1_2014 [3.491]). Furthermore, when comparing the RE values for the NoEC versus EC and NoEC versus FE techniques, most values were greater than 1. On average, the RE values were 1.913 and 1.912, indicating significant improvements of 91.260% and 91.150% with the use of EC and FE, respectively, compared to the conventional NoEC technique. Likewise, comparing the performance of EC and FE techniques revealed an average RE of 2.187, suggesting that FE outperformed EC by 118.710%. Detailed information can be found in **Table S1**.

**Table S1** reveals the results for the Yield_Mg_ha_weight trait, focusing on the MSE. In terms of performance, the use of FE presented the best results in the majority of environments (IAH1ab_2014 [0.598], IAH2_2014 [0.142], ILH1_2014 [0.374], INH1_2014 [0.659], MOH1_2014 [0.199], MOH2_2014 [0.505], NEH2_2014 [0.185] and WIH1_2014 [0.576]). Furthermore, when comparing the RE values for NoEC versus EC and NoEC versus FE techniques, the majority of values were at least 1. On average, the RE values were 1.974 and 2.057, indicating substantial improvements of 97.350% and 105.690% with the use of EC and FE, respectively, compared to the conventional NoEC technique. Moreover, when comparing the performance of EC versus FE techniques based on RE, it was observed that FE outperformed EC in most environments, resulting in an average RE of 2.475. This indicates that FE displayed a performance improvement of 147.490% compared to the use of EC. Detailed information can be found in **Table S1**.

**Figure S1A** presents a comprehensive overview of **Table A1**, comparing various traits across different environments. The results indicate that FE consistently outperformed EC in all traits (Grain_Moisture_BLUE [87.240%], Grain_Moisture_weight [1,1463.900%], Yield_Mg_ha_BLUE [118.710%] and Yield_Mg_ha_weight [147.490%]). These improvements resulted in an average RE of 30.543. On the other hand, the use of EC surpassed NoEC in most traits (Grain_Moisture_BLUE [193.930%], Yield_Mg_ha_BLUE [91.260%] and Yield_Mg_ha_weight [97.350%]), with an average RE of 1.881. Moreover, FE outperformed the conventional NoEC technique in all traits (Grain_Moisture_BLUE [153.690%], Grain_Moisture_weight [396.180%], Yield_Mg_ha_BLUE [91.150%] and Yield_Mg_ha_weight [105.690%]), resulting in an average RE of 2.867. Consequently, the use of EC and FE presented enhancements of 88.13% and 186.680% over NoEC, respectively. These calculations are based on the findings of **Table S1**.

### *Predictor: E+G+GE*

**Table S1** illustrates that the Grain_Moisture_BLUE trait achieved the highest performance with the use of FE across multiple environments, as evidenced by the MSE values (3.565 [IAH1ab_2014], 0.858 [IAH2_2014], 1.289 [IAH3_2014], 1.347 [ILH1_2014], 2.596 [INH1_2014], 1.289 [MOH1_2014], 1.592 [NYH1_2014] and 2.202 [TXH2_2014]). The average RE values indicate that EC and FE surpassed NoEC by 260.900% and 231.450%, respectively. Additionally, when comparing EC and FE based on RE, an average RE of 2.946 was observed, highlighting a superior performance of FE by 194.600%. For further information, refer to **Table S1**.

Considering the Grain_Moisture_weight trait, **Table S1** shows that FE yielded the highest performance in most environments based on the MSE values (DEH1_2014 [5.061], IAHab_2014 [3.968], IAHc_2014 [10.809], IAH4_2014 [1.164], INH1_2014 [17.761], MNH1_2014 [3.768], MOH2_2014 [2639.305], NCH1_2014 [1.785], NEH1_2014 [5.299], NEH2_2014 [3.694], NEH3_2014 [0.908], NYH1_2014 [2.491], TXH2_2014 [34.702], and WIH1_2014 [2.1364]). Likewise, an average RE of 41.160 (EC vs FE) and 8.381 (NoEC vs FE) indicates that FE outperformed EC and NoEC by 40.160% and 7.3810%, respectively. However, when comparing NoEC vs EC techniques, an average RE of 0.7930 implies a decrease in performance of 20.70% for EC compared to NoEC. For detailed information, see **Table S1.**

Focusing on the Yield_Mg_ha_BLUE trait, Table S1 reveals that EC displayed superior performance in most environments based on the MSE values (IAH2_2014 [1.837], ILH1_2014 [5.331], MNH1_2014 [3.889], NEH1_2014 [2.355], NEH2_2014 [2.358], NEH3_2014 [2.203] and WIH1_2014 [2.946]). In addition, the majority of RE values to compare NoEC vs. EC and NoEC vs. FE techniques were above 1, with an average RE of 1.611 and 1.413, respectively. This suggests a general improvement of 61.060% and 41.250% when using EC and FE, respectively, compared to the conventional NoEC technique. Additionally, when comparing the performance of EC and FE techniques, an average RE of 2.322 indicates that FE outperformed EC by 132.170%. For detailed information, see **Table S1**.

Focusing on the Yield_Mg_ha_weight trait, **Table S1** shows that FE exhibited the best performance in most environments based on the MSE values (IAH2_2014 [0.175], IAH3_2014 [0.393], ILH1_2014 [0.373], INH1_2014 [0.711], MNH1_2014 [0.413], MOH1_2014 [0.125], NEH2_2014 [0.172], NEH3_2014 [0.327] and WIH1_2014 [0.571]). The average RE values indicate improvements of 379.410% (NoEC vs EC) and 133.320% (NoEC vs FE), highlighting the advantages of using EC and FE compared to the conventional NoEC technique. Likewise, when comparing EC versus FE techniques based on RE, FE outperformed EC in most environments, resulting in an average RE of 2.285, indicating a superiority of 128.450%. For detailed information, refer seeTable S1.

In **Figure S1B,** a summary of **Table S1** across environments for each trait reveals that FE consistently outperformed EC in all traits (Grain_Moisture_BLUE [194.560%], Grain_Moisture_weight [4016.000%], Yield_Mg_ha_BLUE [132.170%] and Yield_Mg_ha_weight [128.450%]). These improvements resulted in an average RE of 12.178. On the other hand, EC surpassed NoEC in most traits (Grain_Moisture_BLUE [260.900%], Yield_Mg_ha_BLUE [61.060%], and Grain_Moisture_weight [379.410%]), with an average RE of 2.702. Additionally, FE outperformed the conventional NoEC technique in all traits (Grain_Moisture_BLUE [231.450%], Grain_Moisture_weight [738.080%], Yield_Mg_ha_BLUE [41.250%] and Yield_Mg_ha_weight [133.320%]), resulting in an average RE of 3.860. Consequently, the use of EC and FE led to enhancements of 170.170% and 286.030% over NoEC, respectively. These calculations are based on the findings of **Table S1**.

### *Predictor: E+G+BRR*

**Table S2**, presents evidence that the NoEC technique yielded the best performance for the Grain_Moisture_BLUE trait in the majority of environments, as indicated by the MSE values DEH1_2014 (1.189), IAH3_2014 (1.086), IAH4_2014 (1.296), ILH1_2014 (1.409), INH1_2014 (2.748), NEH2_2014 (4.292), and NEH3_2014 (6.397). However, the average RE values suggests that both EC and FE outperformed NoEC, with improvements of 160.240% (NoEC vs EC) and 196.360% (NoEC vs FE). Additionally, when comparing EC and FE techniques based on RE, an average RE of 5.625 reveals that FE outperformed EC by 462.510%. For detailed information, see **Table S2**.

Regarding Grain_Moisture_weight trait, **Table S2** reveals that FE displayed the best performance in most environments based on MSE values (IAH1c_2014 [15.111], IAH2_2014 [0.783], IAH4_2014 [1.982], ILH1_2014 [3.253], INH1_2014 [18.067], MNH1_2014 [6.290], MOH2_2014 [2520.870], NEH1_2014 [6.893], NEH2_2014 [0.521], NEH3_2014 [2.024], NYH1_2014 [3.475] and WIH1_2014 [2.696]). The average RE values indicates significant improvements (7416.400% [EC vs FE] and 693.910% [NoEC vs FE]), highlighting the superiority of FE over EC and NoEC. However, when comparing the NoEC versus EC techniques based on RE, an average RE of 0.712 suggests a general loss of 28.780% for EC compared to NoEC. For detailed information, see **Table S2.**

Focusing on the Yield_Mg_ha_BLUE trait, **Table S2** shows that EC presented a superior performance in most environments, as indicated by the MSE values (IAH2_2014 [1.857], IAH3_2014 [3.238], ILH1_2014 [5.979], INH1_2014 [1.748], MNH1_2014 [3.526], NEH1_2014 [3.254], NEH2_2014 [2.718], NEH3_2014 [2.258] and WIH1_2014 [2.706]). Conversely, when comparing NoEC versus EC and NoEC versus FE techniques based on RE values, most RE values exceeded 1, with average RE values of 2.028 (NoEC vs EC) and 1.829 (NoEC vs FE). This signifies overall improvements of 102.750% and 82.960% for EC and FE, respectively, compared to the conventional NoEC technique. Moreover, when comparing the performance of EC and FE techniques, an average RE of 2.492 highlights that FE outperformed EC by 149.210%. For detailed information, see **Table S2**.

Additionally, focusing on the Yield_Mg_ha_weight trait, **Table S2** illustrates that FE showcased a superior performance in most environments, as indicated by the MSE values (IAH1ab_2014 [0.624], IAH2_2014 [0.145], ILH1_2014 [0.401], INH1_2014 [0.659], MOH1_2014 [0.213] and NEH2_2014 [0.175]). Furthermore, a comparison ofNoEC versus EC and NoEC versus FE techniques based on RE values, showed that most RE values were greater than 1, with average RE values of 1.989 (NoEC vs EC) and 2.085 (NoEC vs FE). This indicates overall improvements of 98.850% and 108.520% for EC and FE, respectively, compared to the conventional NoEC technique. Likewise, when comparing the performance of the EC and FE techniques, an average RE of 2.188 highlights that EC exhibited better performance in most environments, with FE outperforming EC by 118.780%. For detailed information, see **Table S2**.

**Figure S1C** provides a summary of **Table S2** across environments for each trait. It reveals that FE outperformed EC in all traits, with improvements of 162.510% (Grain_Moisture_BLUE), 7416.400% (Grain_Moisture_weight), 149.210% (Yield_Mg_ha_BLUE) and 118.780% (Yield_Mg_ha_weight), resulting in an average RE of 20.617. The use of EC surpassed NoEC in most traits, with improvements of 160.240% (Grain_Moisture_BLUE), 102.750% (Yield_Mg_ha_BLUE), and 98.850% (Grain_Moisture_weight), yielding an average RE of 1.833. Finally, FE outperformed the conventional NoEC technique in all traits, with improvements of 196.360% (Grain_Moisture_BLUE), 693.910% (Grain_Moisture_weight), 82.960% (Yield_Mg_ha_BLUE), and 108.520% (Yield_Mg_ha_weight), resulting in an average RE of 3.704. These findings show that EC and FE outperformed NoEC by 83.270% and 270.440%, respectively. The computations are based on the results of **Table S2**.

### *Predictor: E+G+GE+BRR*

**Table S2** displays that the use of EC led to the best performance for the Grain_Moisture_BLUE trait in most environments, as indicated by the MSE values (3.704 [IAH1ab_2014], 1.702 [ILH1_2014], 2.763 [INH1_2014], 25.267 [MNH1_2014], 1.976 [MOH1_2014], 6.937 [MOH2_2014], 1.861 [NCH1_2014], 3.800 [NEH1_2014] and 4.943 [WIH1_2014]). The average RE values were 3.948 (NoEC vs EC) and 3.131 (NoEC vs FE), indicating that EC and FE outperformed NoEC by 294.760% and 213.060%, respectively. Furthermore, when comparing the performance of EC and FE based on RE, an average RE of 2.716 was observed, indicating that FE outperformed EC by 171.600%. For more detailed information, refer to **Table S2**.

**Table S2** displays that for the Grain_Moisture_weight trait, the use of FE yielded the best performance in most environments. The MSE values in Table A2 support this (0.817 [IAH2_2014], 2.016 [IAH4_2014], 3.233 [MNH1_2014], 2605.419[MOH2_2014], 0.049 [NEH1_2014], 0.065 [NEH2_2014], 0.895 [NEH3_2014], 10.356 [NYH1_2014], 11.546 [TXH2_2014] and 2.108 [WIH1_2014]). Additionally, the average RE values were 1.5306 (NoEC vs EC) and 76.086 (NoEC vs FE), indicating that EC and FE outperformed NoEC by 53.060% and 7508.570%, respectively. Furthermore, when comparing the performance of EC and FE techniques based on RE, an average RE of 164.661 was observed, indicating that FE outperformed EC by 16366.060%. See **Table S2** for more detailed information.

According to **Table S2**, for the Yield_Mg_ha_BLUE trait, the use of EC resulted in the best performance in most environments (IAH1c_2014 [1.433], IAH2_2014 [1.764], ILH1_2014 [4.563], MNH1_2014 [3.874], NEH1_2014 [2.313], NEH2_2014 [2.477] and WIH1_2014 [2.623] based on the MSE values. Additionally, the majority of RE values comparing NoEC versus EC and NoEC versus FE techniques were greater than 1, with an average RE of 1.706 and 1.646, indicating improvements of 70.550% and 64.620% for EC and FE, respectively, compared to the conventional NoEC technique. Moreover, when comparing the performance of EC and FE techniques based on RE, an average RE of 2.666 was observed, indicating that FE outperformed EC by 166.580%. See **Table S2** for more detailed information.

In **Table S2**, for the Yield_Mg_ha_weight trait, the use of FE resulted in the best performance in most environments (IAH2_2014 [0.178], IAH3_2014 [0.356], ILH1_2014 [0.420], INH1_2014 [0.717], MNH1_2014 [0.416], MOH1_2014 [0.172], NCH1_2014 [5.633], NEH2_2014 [0.143], NEH3_2014 [0.326] and NYH1_2014 [5.016]), based on the MSE values. The average RE values were 3.092 (NoEC vs EC) and 2.162 (NoEC vs FE), indicating improvements of 209.240% and 116.170% for EC and FE, respectively, compared to the conventional NoEC technique. Furthermore, when comparing the performance of EC and FE techniques based on RE, the best performance was observed under FE in most environments, resulting in an average RE of 2.246, indicating that FE outperformed EC by 124.640%. Please refer to **Table S2** for more detailed information.

**Figure S1D** provides a summary of **Table S2** across environments for each trait. It shows that FE outperformed EC in all traits, with improvements of 171.600% (Grain_Moisture_BLUE), 16366.060% (Grain_Moisture_weight), 166.580% (Yield_Mg_ha_BLUE and 124.640% (Yield_Mg_ha_weight), resulting in an average RE of 43.072. EC outperformed NoEC in all traits, with improvements of 294.760% (Grain_Moisture_BLUE), 53.060% (Grain_Moisture_weight), 70.550% (Yield_Mg_ha_BLUE) and 209.240% (Grain_Moisture_weight), resulting in an average RE of 2.569. FE outperformed NoEC in all traits, with improvements of 213.060% (Grain_Moisture_BLUE), 7508.570% (Grain_Moisture_weight), 64.620% (Yield_Mg_ha_BLUE) and 116.170% (Yield_Mg_ha_weight), resulting in an average RE of 20.756. Overall, EC and FE outperformed NoEC by 156.910% and 1975.610%, respectively. These results were computed based on the data presented in **Table S2**.

### G2F_2015 dataset

### *Predictor: E+G*

**Table S3** shows that EC yielded the most favorable results for the Grain_Moisture_BLUE trait across multiple environments. Specifically, the MSE values for EC were 10.881(DEH1_2015), 17.035 (GAH1_2015), 6.668 (INH1_2015), 1.660 (NEH1_NEH4_2015), 9.776 (NEH2_2015), 1.144 (NYH2_2015), 9.283 (NYH3_2015), 2.832 (OHH1_2015), and 15.667 (SDH1_2015). On average, the RE values were 2.380 (EC vs. FE) and 1.2665 (NoEC vs. FE), meaning that FE outperformed EC and NoEC by 138.020% and 26.650%, respectively. However, a comparison between EC and NoEC techniques, reveals an average RE of 0.627, suggesting a general decline of 37.340% when EC was used instead of NoEC. For further information, refer to **Table S3**.

Concerning the Grain_Moisture_weight trait, **Table S3** reveals that FE presented a superior performance in terms of MSE across several environments. Specifically, FE yielded the best results in DEH1_2015 (MSE: 14.544), INH1_2015 (MSE: 5.914), MNH1_2015 (MSE: 7.038), NEH1_NEH4_2015 (MSE: 3.542), NYH2_2015 (MSE: 0.724) and NYH3_2015 (MSE: 5.858). Moreover, comparing the conventional NoEC technique, both EC and FE displayed substantial improvements, with average RE values of 5.369 (NoEC vs. EC) and 7.599 (NoEC vs. FE). These values indicate performance enhancements of 436.900% and 659.900%, respectively. Furthermore, when comparing EC and FE techniques, an average RE of 3.376 reveals an overall improvement of 237.560% when FE was used instead of EC. For more comprehensive information, see **Table S3**.

Additionally, regarding the Yield_Mg_ha_BLUE trait, **Table S3** shows that EC delivered the best performance in terms of MSE across several environments. Specifically, EC outperformed other techniques in DEH1_2015 (MSE: 7.328), MNH1_2015 (MSE: 2.551), NCH1_2015 (MSE: 3.247), NYH3_2015 (MSE: 3.604), OHH1_2015 (MSE: 2.258) and SDH1_2015 (MSE: 10.279). On the other hand, most of the RE values, when comparing NoEC versus EC and NoEC versus FE techniques, were greater than 1, with an average RE of 1.149 and 1.025, respectively. These values indicate overall improvements of 14.870% and 2.500% when EC and FE were used, respectively, compared to the conventional NoEC technique. However, when comparing the performance of EC and FE techniques, an average RE of 0.947 suggests a slight decline of 5.280% when FE was used instead of EC. For further details, see **Table S3**.

Meanwhile, regarding the Yield_Mg_ha_weight trait, **Table S3** reveals that the NoEC technique yielded the best performance in terms of MSE across several environments. Notably, NoEC outperformed other techniques in GAH1_2015 (MSE: 0.328), INH1_2015 (MSE: 0.221), MNH1_2015 (MSE: 0.251), NEH3_2015 (MSE: 0.185), and SDH1_2015 (MSE: 0.090). Meanwhile, comparing NoEC versus EC and EC versus FE techniques, reveals that most of the RE values were at least 1, with average RE values of 1.019 (EC vs. NoEC) and 2.056 (FE vs. EC). These values indicate overall improvements of 1.910% and 105.630% when EC and FE were used, respectively, in comparison to the use of NoEC and EC. However, when comparing the performance of NoEC versus FE techniques based on RE, the NoEC technique outperformed FE in most environments, resulting in an average RE of 0.898 and indicating a 10.220% superiority of NoEC over FE. For more detailed information, consult **Table S3**.

**Figure S2A** summarizes the findings from **Table S3** across different traits. It reveals that FE consistently outperformed EC in most traits, displaying improvements of 138.020% (Grain_Moisture_BLUE), 237.560% (Grain_Moisture_weight), and 105.630% (Yield_Mg_ha_weight), resulting in an average RE of 2.189. On the other hand, EC displayed a superior performance over NoEC in several traits, with improvements of 436.900% (Grain_Moisture_weight), 14.870% (Yield_Mg_ha_BLUE), and 1.910% (Yield_Mg_ha_weight), resulting in an average RE of 2.041. Additionally, FE outperformed NoEC in multiple traits, with improvements of 26.650% (Grain_Moisture_BLUE), 659.90% (Grain_Moisture_weight) and 2.500% (Yield_Mg_ha_BLUE), resulting in an average RE of 3.014. These findings indicate that both EC and FE techniques surpassed the performance of NoEC by 104.090% and 201.370%, respectively. These calculations are based on the results obtained from **Table S3**.

### *Predictor: E+G+GE*

**Table S3** shows that the NoEC technique delivered the best performance for the Grain_Moisture_BLUE trait in most environments, as evidenced by the following MSE values: 8.293 (DEH1_2015), 2.106 (INH1_2015), 9.531 (NEH2_2015), 4.615 (NEH3_2015), 9.145 (NYH3_2015), and 14.536 (SDH1_2015). On average, the RE values were 2.790 (EC vs. FE) and 1.211 (NoEC vs. FE), indicating that FE outperformed EC and NoEC by 179.000% and 21.120%, respectively. Furthermore, when comparing the performance of NoEC and EC techniques, an average RE of 0.753 reveals that NoEC outperformed EC by 24.700%. For more specific information, see **Table S3**.

For the Grain_Moisture_weight trait, **Table S3** reveals that EC exhibited a superior performance in terms of MSE across several environments. Specifically, EC outperformed other techniques in GAH1_2015 (MSE: 5.0613), MNH1_2015 (MSE: 2.744), NCH1_2015 (MSE: 37.988), OHH1_2015 (MSE: 0.438) and ADH1_2015 (MSE: 1.939). Additionally, the average RE values of 2.578 (EC vs. FE) and 14.079 (NoEC vs. FE) indicate that FE outperformed EC and NoEC by 157.830% and 1307.900%, respectively. Moreover, when comparing the performance of NoEC versus EC techniques, an average RE of 13.376 suggests a substantial improvement of 1237.630% when EC was used instead of NoEC. For more detailed information, see **Table S3**.

In addition, regarding the Yield_Mg_ha_BLUE trait, **Table S3** illustrates that EC yielded the best performance in terms of MSE across most environments (DEH1_2015 [MSE: 6.861], MNH1_2015 [MSE: 2.487], NCH1_2015 [MSE: 3.163], NYH3_2015 [MSE: 3.520] and SDH1_2015 [MSE: 10.664]). On the other hand, when comparing NoEC versus EC and EC versus FE, most of the RE values were greater than 1, with an average RE of 1.136 (NoEC vs. EC) and 1.014 (EC vs. FE). These values indicate overall improvements of 13.590% and 1.410% when EC and FE were used, respectively, compared to the conventional NoEC and EC techniques. However, when comparing the performance of NoEC and FE techniques based on RE, an average RE of 0.948 indicates that NoEC outperformed FE by 5.190%. For more detailed information, see **Table S3**.

Meanwhile, for the Yield_Mg_ha_weight trait, **Table S3** shows that NoEC achieved the best performance in terms of MSE in most environments, specifically MNH1_2015 (MSE: 0.168), NEH1_NEH4_2015 (MSE: 0.347), NEH3_2015 (MSE: 0.109), NYH3_2015 (MSE: 0.276) and SDH1_2015 (MSE: 0.095). The average RE values were 1.312 (NoEC vs. EC) and 1.336 (NoEC vs. FE), indicating general improvements of 31.150% and 33.560% when using EC and FE, respectively, compared to the conventional NoEC technique. Furthermore, when comparing EC versus FE techniques based on RE, NoEC displayed a superior performance in most environments, resulting in an average RE of 2.221. This means that FE outperformed EC by 122.100%. For more detailed information, see **Table S3**.

Finally, **Figure S2B** provides a comprehensive summary of **Table S3** across different environments for each trait. It clearly illustrates that FE yielded superior results compared to EC in all traits, with performance improvements of 179.000% (Grain_Moisture_BLUE), 157.830% (Grain_Moisture_weight), 1.410% (Yield_Mg_ha_BLUE) and 122.080% (Yield_Mg_ha_weight), resulting in an average RE of 2.150. Additionally, EC outperformed NoEC in the majority of traits, with substantial improvements of 1237.630% (Grain_Moisture_weight), 13.590% (Yield_Mg_ha_BLUE) and 31.150% (Grain_Moisture_weight), leading to an average RE of 4.144. Moreover, FE displayed superiority over NoEC in most traits, with performance gains of 21.120% (Grain_Moisture_BLUE), 1307.900% (Grain_Moisture_weight), and 33.560% (Yield_Mg_ha_weight), resulting in an average RE of 4.394. These findings unequivocally show that the use of EC and FE outperformed the use of NoEC by 314.420% and 339.350%, respectively. The computations were based on the results obtained from **Table S3**.

### *Predictor: E+G+BRR*

**Table S4** indicates that the use of FE yielded superior results for the Grain_Moisture_BLUE trait across various environments. Notably, it achieved lower MSE values, such as 5.231 (DEH1_2015), 16.333 (GAH1_2015), 1.634 (NEH1_NEH4_2015), 8.281 (NEH2_2015), 5.459 (NEH3_2015), 5.884 (NYH3_2015), 2.360 (OHH1_2015) and 13.217 (SDH1_2015). On average, FE outperformed NoEC and EC with RE values of 2.741 and 1.426, respectively, indicating improvements of 174.060% and 42.580%. Furthermore, the comparison between NoEC and EC techniques revealed an average RE of 0.702, indicating that NoEC outperformed EC by 29.770%. Additional information can be found in **Table S4**.

**Table S4** highlights the superiority of FE in terms of the Grain_Moisture_weight trait across multiple environments. Notably, FE achieved the best performance with lower MSE values in the following environments: DEH1_2015 (0.445), INH1_2015 (1.782), MNH1_2015 (5.716), NEH1_NEH4_2015 (3.552), NEH2_2015 (0.927), NYH2_2015 (0.714), NYH3_2015 (1.966) and OHH1_2015 (0.351). On average, FE outperformed EC and NoEC with RE values of 9.225 and 15.107, indicating significant improvements of 822.500% and 1410.700%, respectively. In addition, when comparing the performance of NoEC and EC techniques, an average RE of 2.769 was observed, indicating a general improvement of 176.900% for the use of EC compared to NoEC. For more detailed information, see **Table S4**.

In **Table S4** it is clear that EC yielded the best results for the Yield_Mg_ha_BLUE trait across various environments, as indicated by the lower MSE values (DEH1_2015 [7.063], INH1_2015 [2.704], MNH1_2015 [2.376], NCH1_2015 [3.287], NEH3_2015 [3.749], NYH3_2015 [3.589] and SDH1_2015 [10.116]). On the other hand, when considering the average RE values, a different pattern emerges. The use of FE presented less efficiency compared to EC and NoEC, with average RE values of 0.719 and 0.753, respectively, resulting in losses of 28.140% and 24.680%. Conversely, the comparison between NoEC and EC techniques revealed an average RE of 1.150, indicating a 15.000% improvement in favor of EC. For more comprehensive information, see **Table S4**.

Regarding the Yield_Mg_ha_weight trait, **Table S4** reveals that the use of NoEC achieved the best performance in most environments, as evidenced by the lower MSE values (GAH1_2015 [0.328], INH1_2015 [0.221], MNH1_2015 [0.251], NEH1_NEH4_2015 [0.238], NEH3_2015 [0.141] and SDH1_2015 [0.090]). When considering the average RE values, both EC and FE techniques presented improvements compared to the conventional NoEC technique. The use of EC and FE resulted in average RE values of 1.114 and 1.132, respectively, indicating general improvements of 11.400% and 13.200%. However, when comparing EC and FE techniques, it is worth noting that FE performed better in most environments, yielding an average RE of 2.386, and indicating that FE outperformed EC by 138.600%. For a more detailed analysis, refer to **Table S4**.

**Figure S2C** provides a comprehensive summary of the results presented in **Table S4** for each trait. It shows that FE presented a superior performance compared to EC in the majority of traits, with improvements of 147.060% (Grain_Moisture_BLUE), 822.480% (Grain_Moisture_weight) and 138.560% (Yield_Mg_ha_weight), resulting in an average RE of 3.699. Additionally, EC outperformed NoEC in most traits, with improvements of 176.900% (Grain_Moisture_weight), 15.000% (Yield_Mg_ha_BLUE) and 11.410% (Grain_Moisture_weight), yielding an average RE of 1.434. Furthermore, FE outperformed NoEC in most traits, with improvements of 42.580% (Grain_Moisture_BLUE), 1410.660% (Grain_Moisture_weight), and 13.180% (Yield_Mg_ha_weight), resulting in an average RE of 4.604. These findings indicate that the use of EC and FE surpassed the performance of NoEC by 43.390% and 42.580%, respectively. The calculations are derived from the results presented in **Table S4**.

### *Predictor: E+G+GE+BRR*

Table S4 highlights that the use of FE yielded the best performance for the Grain_Moisture_BLUE trait across most environments, as evidenced by the MSE values (5.938 [GAH1_2015], 1.189 [MNH1_2015], 1.795 [NEH1_NEH4_2015], 6.012 [NYH3_2015] and 13.882 [SDH1_2015]). In addition, the average RE values further reveal the superiority of FE, with improvements of 157.900% (EC vs FE) and 20.610% (NoEC vs FE) observed. In contrast, when comparing NoEC and EC techniques, an average RE of 0.748 indicates that NoEC outperformed EC by 25.210%. For additional information, refer to **Table S4**.

**Table S4** presents the MSE results for the Grain_Moisture_weight trait, revealing that the use of FE demonstrated superior performance in most environments: DEH1_2015 (0.485), INH1_2015 (1.469), NEH1_NEH4_2015 (3.306), NEH2_2015 (0.140), NEH3_2015 (1.711), NYH2_2015 (1.960), and NYH3_2015 (2.199). Furthermore, the average RE values indicate substantial improvements compared to the conventional NoEC approach, with EC and FE outperforming NoEC by 5202.050% and 2782.700%, respectively. Additionally, when comparing EC and FE techniques, an average RE of 10.797 demonstrates that FE surpassed EC by 979.710%. Further details can be found in **Table S4**.

**Table S4** presents the MSE results for the Yield_Mg_ha_BLUE trait, indicating that the use of NoEC yielded the best performance in most environments (GAH1_2015 [2.833], INH1_2015 [2.710], NEH1_NEH4_2015 [5.144], NEH2_2015 [1.739], NEH3_2015 [1.759] and OHH1_2015 [2.447]). On the other hand, the majority of RE values comparing NoEC and EC were greater than 1, with an average RE of 1.147, implying a general improvement of 14.740% when using EC instead of the conventional NoEC technique. However, when comparing FE with EC and NoEC, average RE values of 0.782 and 0.772 were observed, indicating losses of 21.800% and 22.770% when using FE compared to EC and NoEC, respectively. See **Table S4** for further details.

**Table S4** provides the MSE results for the Yield_Mg_ha_weight trait, revealing that the use of EC yielded the best performance in most environments (DEH1_2015 [0.040], NEH1_NEH4_2015 [0.173], NEH2_2015 [0.076], NYH3_2015 [0.211] and OHH1_2015 [2.367]). The average RE values were 1.139 (NoEC vs EC) and 1.403 (NoEC vs FE), indicating general improvements of 13.950% and 40.280% when using EC and FE, respectively, in comparison with the conventional NoEC technique. Moreover, when comparing the performance of EC versus FE, the use of EC displayed a lower performance in most environments, resulting in an average RE of 2.953. This implies that FE outperformed the use of EC by 195.270%. See **Table S4** for more detailed information.

**Figure S2D** presents a summary of the results from **Table S4** across different environments for each trait. It reveals that FE outperformed EC in most traits, with improvements of 157.900% (Grain_Moisture_BLUE), 979.710% (Grain_Moisture_weight) and 195.270% (Yield_Mg_ha_weight), resulting in an average RE of 4.278. Likewise, EC outperformed NoEC in most traits, with improvements of 5,202.050% (Grain_Moisture_weight), 14.740% (Yield_Mg_ha_BLUE), and 13.950% (Grain_Moisture_weight), resulting in an average RE of 14.014. Furthermore, FE outperformed NoEC in most traits, with improvements of 20.610% (Grain_Moisture_BLUE), 2,782.700% (Grain_Moisture_weight), and 40.280% (Yield_Mg_ha_weight), resulting in an average RE of 8.052. Overall, EC and FE outperformed NoEC by 1,301.380% and 705.210%, respectively. These calculations are based on the results presented in **Table S4**.

**Table S1**. The prediction performance and the relative efficiency (RE) for **G2F_2014** **dataset** in terms of mean squared error (MSE) for each Environment and for each trait, for the predictors E+G and E+G+GE under three different techniques to compute the Kernel for the effect of the Environment: without Environmental Covariates (No.EC), using Environmental covariates (EC) and using Environmental Covariates with Feature Engineering (FE).

| Predictor | Trait | Env | No.EC | EC | FE | NoEC_vs_EC | EC_vs_FE | NoEC_vs_FE |
| --- | --- | --- | --- | --- | --- | --- | --- | --- |
| E+G | Grain_Moisture_BLUE | DEH1_2014 | 1.190 | 17.778 | 4.190 | 0.067 | 4.243 | 0.284 |
| E+G | Grain_Moisture_BLUE | IAH1ab_2014 | 19.039 | 4.565 | 5.362 | 4.171 | 0.851 | 3.551 |
| E+G | Grain_Moisture_BLUE | IAH1c_2014 | 1.258 | 1.314 | 2.312 | 0.958 | 0.568 | 0.544 |
| E+G | Grain_Moisture_BLUE | IAH2_2014 | 20.289 | 1.575 | 0.988 | 12.885 | 1.594 | 20.544 |
| E+G | Grain_Moisture_BLUE | IAH3_2014 | 1.086 | 3.242 | 1.365 | 0.335 | 2.375 | 0.796 |
| E+G | Grain_Moisture_BLUE | IAH4_2014 | 1.296 | 9.373 | 5.117 | 0.138 | 1.832 | 0.253 |
| E+G | Grain_Moisture_BLUE | ILH1_2014 | 1.409 | 2.110 | 2.473 | 0.668 | 0.853 | 0.570 |
| E+G | Grain_Moisture_BLUE | INH1_2014 | 2.748 | 4.697 | 2.863 | 0.585 | 1.641 | 0.960 |
| E+G | Grain_Moisture_BLUE | MNH1_2014 | 114.022 | 20.117 | 46.172 | 5.668 | 0.436 | 2.470 |
| E+G | Grain_Moisture_BLUE | MOH1_2014 | 3.164 | 3.849 | 4.512 | 0.822 | 0.853 | 0.701 |
| E+G | Grain_Moisture_BLUE | MOH2_2014 | 59.809 | 9.640 | 21.497 | 6.204 | 0.448 | 2.782 |
| E+G | Grain_Moisture_BLUE | NCH1_2014 | 21.863 | 1.873 | 13.242 | 11.676 | 0.141 | 1.651 |
| E+G | Grain_Moisture_BLUE | NEH1_2014 | 9.572 | 6.253 | 11.010 | 1.531 | 0.568 | 0.869 |
| E+G | Grain_Moisture_BLUE | NEH2_2014 | 4.292 | 27.747 | 3.889 | 0.155 | 7.134 | 1.104 |
| E+G | Grain_Moisture_BLUE | NEH3_2014 | 6.397 | 4.248 | 8.569 | 1.506 | 0.496 | 0.747 |
| E+G | Grain_Moisture_BLUE | NYH1_2014 | 5.044 | 2.657 | 2.910 | 1.898 | 0.913 | 1.733 |
| E+G | Grain_Moisture_BLUE | TXH2_2014 | 30.572 | 53.272 | 6.401 | 0.574 | 8.323 | 4.776 |
| E+G | Grain_Moisture_BLUE | WIH1_2014 | 16.297 | 5.314 | 12.253 | 3.067 | 0.434 | 1.330 |
| E+G | Grain_Moisture_BLUE | Across | - | - | - | 2.939 | 1.872 | 2.537 |
| E+G | Grain_Moisture_weight | DEH1_2014 | 22.520 | 298.800 | 68.872 | 0.075 | 4.339 | 0.327 |
| E+G | Grain_Moisture_weight | IAH1ab_2014 | 18.024 | 4.293 | 25.996 | 4.199 | 0.165 | 0.693 |
| E+G | Grain_Moisture_weight | IAH1c_2014 | 17.107 | 630.026 | 23.415 | 0.027 | 26.907 | 0.731 |
| E+G | Grain_Moisture_weight | IAH2_2014 | 23.359 | 11.064 | 7.870 | 2.111 | 1.406 | 2.968 |
| E+G | Grain_Moisture_weight | IAH3_2014 | 18.307 | 5.172 | 6.633 | 3.540 | 0.780 | 2.760 |
| E+G | Grain_Moisture_weight | IAH4_2014 | 3.554 | 266.969 | 10.503 | 0.013 | 25.418 | 0.338 |
| E+G | Grain_Moisture_weight | ILH1_2014 | 27.737 | 49.960 | 3.328 | 0.555 | 15.012 | 8.334 |
| E+G | Grain_Moisture_weight | INH1_2014 | 26.397 | 626.393 | 6.828 | 0.042 | 91.735 | 3.866 |
| E+G | Grain_Moisture_weight | MNH1_2014 | 49.919 | 333.688 | 1.566 | 0.150 | 213.137 | 31.885 |
| E+G | Grain_Moisture_weight | MOH1_2014 | 13.044 | 739.097 | 63.617 | 0.018 | 11.618 | 0.205 |
| E+G | Grain_Moisture_weight | MOH2_2014 | 3085.779 | 2655.364 | 2618.855 | 1.162 | 1.014 | 1.178 |
| E+G | Grain_Moisture_weight | NCH1_2014 | 1.903 | 194.672 | 6.746 | 0.010 | 28.858 | 0.282 |
| E+G | Grain_Moisture_weight | NEH1_2014 | 18.198 | 889.235 | 8.960 | 0.021 | 99.241 | 2.031 |
| E+G | Grain_Moisture_weight | NEH2_2014 | 17.497 | 158.561 | 7.572 | 0.110 | 20.942 | 2.311 |
| E+G | Grain_Moisture_weight | NEH3_2014 | 61.654 | 316.846 | 34.173 | 0.195 | 9.272 | 1.804 |
| E+G | Grain_Moisture_weight | NYH1_2014 | 21.524 | 82.824 | 3.332 | 0.260 | 24.859 | 6.460 |
| E+G | Grain_Moisture_weight | TXH2_2014 | 17.256 | 1355.425 | 0.927 | 0.013 | 1461.847 | 18.610 |
| E+G | Grain_Moisture_weight | WIH1_2014 | 26.520 | 263.322 | 5.858 | 0.101 | 44.952 | 4.527 |
| E+G | Grain_Moisture_weight | Across | - | - | - | 0.700 | 115.639 | 4.962 |
| E+G | Yield_Mg_ha_BLUE | DEH1_2014 | 2.361 | 16.577 | 6.113 | 0.142 | 2.712 | 0.386 |
| E+G | Yield_Mg_ha_BLUE | IAH1ab_2014 | 5.568 | 2.482 | 2.145 | 2.243 | 1.157 | 2.596 |
| E+G | Yield_Mg_ha_BLUE | IAH1c_2014 | 1.677 | 2.707 | 4.333 | 0.620 | 0.625 | 0.387 |
| E+G | Yield_Mg_ha_BLUE | IAH2_2014 | 5.476 | 1.899 | 2.314 | 2.883 | 0.821 | 2.367 |
| E+G | Yield_Mg_ha_BLUE | IAH3_2014 | 20.378 | 5.601 | 4.147 | 3.638 | 1.351 | 4.914 |
| E+G | Yield_Mg_ha_BLUE | IAH4_2014 | 3.651 | 6.954 | 2.553 | 0.525 | 2.725 | 1.431 |
| E+G | Yield_Mg_ha_BLUE | ILH1_2014 | 8.269 | 4.429 | 8.171 | 1.867 | 0.542 | 1.012 |
| E+G | Yield_Mg_ha_BLUE | INH1_2014 | 3.560 | 1.722 | 3.901 | 2.067 | 0.442 | 0.913 |
| E+G | Yield_Mg_ha_BLUE | MNH1_2014 | 5.844 | 3.603 | 10.430 | 1.622 | 0.346 | 0.560 |
| E+G | Yield_Mg_ha_BLUE | MOH1_2014 | 4.122 | 8.275 | 1.916 | 0.498 | 4.319 | 2.152 |
| E+G | Yield_Mg_ha_BLUE | MOH2_2014 | 2.528 | 2.807 | 4.272 | 0.901 | 0.657 | 0.592 |
| E+G | Yield_Mg_ha_BLUE | NCH1_2014 | 8.638 | 7.888 | 3.166 | 1.095 | 2.492 | 2.729 |
| E+G | Yield_Mg_ha_BLUE | NEH1_2014 | 12.015 | 3.920 | 9.414 | 3.065 | 0.416 | 1.276 |
| E+G | Yield_Mg_ha_BLUE | NEH2_2014 | 11.924 | 2.758 | 3.024 | 4.323 | 0.912 | 3.944 |
| E+G | Yield_Mg_ha_BLUE | NEH3_2014 | 9.545 | 1.867 | 1.972 | 5.112 | 0.947 | 4.839 |
| E+G | Yield_Mg_ha_BLUE | NYH1_2014 | 1.488 | 178.355 | 10.983 | 0.008 | 16.239 | 0.136 |
| E+G | Yield_Mg_ha_BLUE | TXH2_2014 | 10.679 | 7.374 | 3.174 | 1.448 | 2.323 | 3.365 |
| E+G | Yield_Mg_ha_BLUE | WIH1_2014 | 8.269 | 3.491 | 10.196 | 2.369 | 0.342 | 0.811 |
| E+G | Yield_Mg_ha_BLUE | Across | - | - | - | 1.913 | 2.187 | 1.912 |
| E+G | Yield_Mg_ha_weight | DEH1_2014 | 0.388 | 10.415 | 0.850 | 0.037 | 12.252 | 0.456 |
| E+G | Yield_Mg_ha_weight | IAH1ab_2014 | 0.706 | 0.988 | 0.598 | 0.715 | 1.652 | 1.180 |
| E+G | Yield_Mg_ha_weight | IAH1c_2014 | 1.179 | 1.011 | 1.061 | 1.166 | 0.954 | 1.112 |
| E+G | Yield_Mg_ha_weight | IAH2_2014 | 0.145 | 0.712 | 0.142 | 0.203 | 5.002 | 1.016 |
| E+G | Yield_Mg_ha_weight | IAH3_2014 | 0.187 | 1.668 | 0.460 | 0.112 | 3.624 | 0.407 |
| E+G | Yield_Mg_ha_weight | IAH4_2014 | 0.211 | 0.541 | 0.353 | 0.391 | 1.532 | 0.598 |
| E+G | Yield_Mg_ha_weight | ILH1_2014 | 1.621 | 1.330 | 0.374 | 1.219 | 3.560 | 4.338 |
| E+G | Yield_Mg_ha_weight | INH1_2014 | 2.246 | 1.003 | 0.660 | 2.241 | 1.520 | 3.405 |
| E+G | Yield_Mg_ha_weight | MNH1_2014 | 0.399 | 0.385 | 0.390 | 1.036 | 0.986 | 1.022 |
| E+G | Yield_Mg_ha_weight | MOH1_2014 | 0.989 | 0.381 | 0.200 | 2.599 | 1.907 | 4.955 |
| E+G | Yield_Mg_ha_weight | MOH2_2014 | 0.958 | 0.575 | 0.505 | 1.667 | 1.138 | 1.897 |
| E+G | Yield_Mg_ha_weight | NCH1_2014 | 3.087 | 5.732 | 3.638 | 0.539 | 1.576 | 0.849 |
| E+G | Yield_Mg_ha_weight | NEH1_2014 | 0.848 | 0.068 | 0.544 | 12.507 | 0.125 | 1.558 |
| E+G | Yield_Mg_ha_weight | NEH2_2014 | 1.274 | 0.574 | 0.185 | 2.219 | 3.097 | 6.871 |
| E+G | Yield_Mg_ha_weight | NEH3_2014 | 0.361 | 0.913 | 0.384 | 0.395 | 2.375 | 0.939 |
| E+G | Yield_Mg_ha_weight | NYH1_2014 | 5.519 | 5.077 | 5.337 | 1.087 | 0.951 | 1.034 |
| E+G | Yield_Mg_ha_weight | TXH2_2014 | 0.997 | 0.154 | 0.261 | 6.476 | 0.590 | 3.820 |
| E+G | Yield_Mg_ha_weight | WIH1_2014 | 0.902 | 0.985 | 0.576 | 0.916 | 1.710 | 1.567 |
| E+G | Yield_Mg_ha_weight | Across | - | - | - | 1.974 | 2.475 | 2.057 |
| E+G+GE | Grain_Moisture_BLUE | DEH1_2014 | 1.176 | 18.563 | 2.061 | 0.063 | 9.005 | 0.571 |
| E+G+GE | Grain_Moisture_BLUE | IAH1ab_2014 | 21.293 | 3.862 | 3.565 | 5.513 | 1.083 | 5.973 |
| E+G+GE | Grain_Moisture_BLUE | IAH1c_2014 | 13.583 | 1.048 | 1.204 | 12.967 | 0.870 | 11.284 |
| E+G+GE | Grain_Moisture_BLUE | IAH2_2014 | 7.255 | 1.341 | 0.858 | 5.411 | 1.562 | 8.453 |
| E+G+GE | Grain_Moisture_BLUE | IAH3_2014 | 3.085 | 3.197 | 1.289 | 0.965 | 2.481 | 2.394 |
| E+G+GE | Grain_Moisture_BLUE | IAH4_2014 | 1.197 | 9.928 | 3.817 | 0.121 | 2.601 | 0.314 |
| E+G+GE | Grain_Moisture_BLUE | ILH1_2014 | 3.839 | 1.411 | 1.347 | 2.721 | 1.047 | 2.850 |
| E+G+GE | Grain_Moisture_BLUE | INH1_2014 | 3.230 | 8.896 | 2.596 | 0.363 | 3.427 | 1.244 |
| E+G+GE | Grain_Moisture_BLUE | MNH1_2014 | 73.658 | 28.308 | 46.142 | 2.602 | 0.614 | 1.596 |
| E+G+GE | Grain_Moisture_BLUE | MOH1_2014 | 10.349 | 1.472 | 1.290 | 7.032 | 1.141 | 8.024 |
| E+G+GE | Grain_Moisture_BLUE | MOH2_2014 | 55.131 | 8.678 | 21.622 | 6.353 | 0.401 | 2.550 |
| E+G+GE | Grain_Moisture_BLUE | NCH1_2014 | 29.901 | 2.318 | 30.920 | 12.900 | 0.075 | 0.967 |
| E+G+GE | Grain_Moisture_BLUE | NEH1_2014 | 11.048 | 6.994 | 12.666 | 1.580 | 0.552 | 0.872 |
| E+G+GE | Grain_Moisture_BLUE | NEH2_2014 | 4.302 | 24.526 | 4.189 | 0.175 | 5.855 | 1.027 |
| E+G+GE | Grain_Moisture_BLUE | NEH3_2014 | 3.418 | 3.701 | 7.427 | 0.923 | 0.498 | 0.460 |
| E+G+GE | Grain_Moisture_BLUE | NYH1_2014 | 3.699 | 3.261 | 1.952 | 1.134 | 1.671 | 1.895 |
| E+G+GE | Grain_Moisture_BLUE | TXH2_2014 | 16.714 | 43.404 | 2.202 | 0.385 | 19.711 | 7.590 |
| E+G+GE | Grain_Moisture_BLUE | WIH1_2014 | 18.051 | 4.810 | 11.302 | 3.753 | 0.426 | 1.597 |
| E+G+GE | Grain_Moisture_BLUE | Across | - | - | - | 3.609 | 2.946 | 3.315 |
| E+G+GE | Grain_Moisture_weight | DEH1_2014 | 10.659 | 167.696 | 5.061 | 0.064 | 33.133 | 2.106 |
| E+G+GE | Grain_Moisture_weight | IAH1ab_2014 | 22.067 | 11.409 | 3.968 | 1.934 | 2.875 | 5.561 |
| E+G+GE | Grain_Moisture_weight | IAH1c_2014 | 11.930 | 1751.896 | 10.809 | 0.007 | 162.072 | 1.104 |
| E+G+GE | Grain_Moisture_weight | IAH2_2014 | 8.238 | 188.563 | 14.452 | 0.044 | 13.047 | 0.570 |
| E+G+GE | Grain_Moisture_weight | IAH3_2014 | 9.865 | 26.342 | 38.663 | 0.375 | 0.681 | 0.255 |
| E+G+GE | Grain_Moisture_weight | IAH4_2014 | 13.934 | 89.452 | 1.164 | 0.156 | 76.849 | 11.970 |
| E+G+GE | Grain_Moisture_weight | ILH1_2014 | 15.045 | 294.236 | 112.627 | 0.051 | 2.613 | 0.134 |
| E+G+GE | Grain_Moisture_weight | INH1_2014 | 25.821 | 68.221 | 17.761 | 0.379 | 3.841 | 1.454 |
| E+G+GE | Grain_Moisture_weight | MNH1_2014 | 35.611 | 240.845 | 3.768 | 0.148 | 63.912 | 9.450 |
| E+G+GE | Grain_Moisture_weight | MOH1_2014 | 22.565 | 921.530 | 56.544 | 0.025 | 16.298 | 0.399 |
| E+G+GE | Grain_Moisture_weight | MOH2_2014 | 2947.225 | 2828.066 | 2639.305 | 1.042 | 1.072 | 1.117 |
| E+G+GE | Grain_Moisture_weight | NCH1_2014 | 2.367 | 141.838 | 1.785 | 0.017 | 79.483 | 1.326 |
| E+G+GE | Grain_Moisture_weight | NEH1_2014 | 39.278 | 498.704 | 5.299 | 0.079 | 94.122 | 7.413 |
| E+G+GE | Grain_Moisture_weight | NEH2_2014 | 27.701 | 201.417 | 3.694 | 0.138 | 54.533 | 7.500 |
| E+G+GE | Grain_Moisture_weight | NEH3_2014 | 70.926 | 7.821 | 0.908 | 9.068 | 8.612 | 78.095 |
| E+G+GE | Grain_Moisture_weight | NYH1_2014 | 16.284 | 132.631 | 2.491 | 0.123 | 53.248 | 6.538 |
| E+G+GE | Grain_Moisture_weight | TXH2_2014 | 48.764 | 1747.630 | 34.702 | 0.028 | 50.362 | 1.405 |
| E+G+GE | Grain_Moisture_weight | WIH1_2014 | 30.889 | 51.550 | 2.136 | 0.599 | 24.129 | 14.458 |
| E+G+GE | Grain_Moisture_weight | Across | - | - | - | 0.793 | 41.160 | 8.381 |
| E+G+GE | Yield_Mg_ha_BLUE | DEH1_2014 | 5.741 | 17.650 | 8.009 | 0.325 | 2.204 | 0.717 |
| E+G+GE | Yield_Mg_ha_BLUE | IAH1ab_2014 | 3.587 | 2.266 | 1.817 | 1.583 | 1.247 | 1.974 |
| E+G+GE | Yield_Mg_ha_BLUE | IAH1c_2014 | 1.578 | 2.135 | 2.758 | 0.739 | 0.774 | 0.572 |
| E+G+GE | Yield_Mg_ha_BLUE | IAH2_2014 | 3.520 | 1.837 | 2.358 | 1.917 | 0.779 | 1.493 |
| E+G+GE | Yield_Mg_ha_BLUE | IAH3_2014 | 12.825 | 6.994 | 5.185 | 1.834 | 1.349 | 2.473 |
| E+G+GE | Yield_Mg_ha_BLUE | IAH4_2014 | 9.901 | 7.779 | 2.436 | 1.273 | 3.194 | 4.065 |
| E+G+GE | Yield_Mg_ha_BLUE | ILH1_2014 | 6.462 | 5.331 | 7.061 | 1.212 | 0.755 | 0.915 |
| E+G+GE | Yield_Mg_ha_BLUE | INH1_2014 | 1.324 | 3.355 | 3.430 | 0.395 | 0.978 | 0.386 |
| E+G+GE | Yield_Mg_ha_BLUE | MNH1_2014 | 7.807 | 3.889 | 8.432 | 2.007 | 0.461 | 0.926 |
| E+G+GE | Yield_Mg_ha_BLUE | MOH1_2014 | 1.713 | 8.410 | 2.590 | 0.204 | 3.247 | 0.662 |
| E+G+GE | Yield_Mg_ha_BLUE | MOH2_2014 | 2.618 | 2.964 | 5.111 | 0.883 | 0.580 | 0.512 |
| E+G+GE | Yield_Mg_ha_BLUE | NCH1_2014 | 8.503 | 12.398 | 9.720 | 0.686 | 1.276 | 0.875 |
| E+G+GE | Yield_Mg_ha_BLUE | NEH1_2014 | 9.048 | 2.355 | 7.021 | 3.843 | 0.335 | 1.289 |
| E+G+GE | Yield_Mg_ha_BLUE | NEH2_2014 | 10.312 | 2.358 | 2.638 | 4.374 | 0.894 | 3.909 |
| E+G+GE | Yield_Mg_ha_BLUE | NEH3_2014 | 10.025 | 2.203 | 3.952 | 4.552 | 0.557 | 2.537 |
| E+G+GE | Yield_Mg_ha_BLUE | NYH1_2014 | 1.779 | 173.868 | 9.694 | 0.010 | 17.936 | 0.184 |
| E+G+GE | Yield_Mg_ha_BLUE | TXH2_2014 | 3.449 | 15.861 | 3.217 | 0.218 | 4.930 | 1.072 |
| E+G+GE | Yield_Mg_ha_BLUE | WIH1_2014 | 8.653 | 2.946 | 10.005 | 2.937 | 0.295 | 0.865 |
| E+G+GE | Yield_Mg_ha_BLUE | Across | - | - | - | 1.611 | 2.322 | 1.413 |
| E+G+GE | Yield_Mg_ha_weight | DEH1_2014 | 0.601 | 10.872 | 1.218 | 0.055 | 8.926 | 0.494 |
| E+G+GE | Yield_Mg_ha_weight | IAH1ab_2014 | 0.489 | 0.941 | 0.633 | 0.519 | 1.486 | 0.771 |
| E+G+GE | Yield_Mg_ha_weight | IAH1c_2014 | 1.375 | 0.934 | 0.970 | 1.472 | 0.963 | 1.417 |
| E+G+GE | Yield_Mg_ha_weight | IAH2_2014 | 0.319 | 0.757 | 0.175 | 0.421 | 4.324 | 1.822 |
| E+G+GE | Yield_Mg_ha_weight | IAH3_2014 | 0.413 | 1.683 | 0.393 | 0.245 | 4.280 | 1.049 |
| E+G+GE | Yield_Mg_ha_weight | IAH4_2014 | 0.168 | 0.477 | 0.318 | 0.353 | 1.500 | 0.529 |
| E+G+GE | Yield_Mg_ha_weight | ILH1_2014 | 0.806 | 1.430 | 0.373 | 0.563 | 3.834 | 2.160 |
| E+G+GE | Yield_Mg_ha_weight | INH1_2014 | 0.753 | 0.998 | 0.711 | 0.755 | 1.404 | 1.060 |
| E+G+GE | Yield_Mg_ha_weight | MNH1_2014 | 1.927 | 0.418 | 0.413 | 4.613 | 1.011 | 4.665 |
| E+G+GE | Yield_Mg_ha_weight | MOH1_2014 | 0.202 | 0.371 | 0.125 | 0.545 | 2.977 | 1.622 |
| E+G+GE | Yield_Mg_ha_weight | MOH2_2014 | 0.112 | 0.552 | 0.351 | 0.203 | 1.574 | 0.320 |
| E+G+GE | Yield_Mg_ha_weight | NCH1_2014 | 6.787 | 9.096 | 7.570 | 0.746 | 1.202 | 0.897 |
| E+G+GE | Yield_Mg_ha_weight | NEH1_2014 | 1.080 | 0.032 | 0.301 | 33.753 | 0.106 | 3.590 |
| E+G+GE | Yield_Mg_ha_weight | NEH2_2014 | 1.191 | 0.513 | 0.172 | 2.323 | 2.990 | 6.945 |
| E+G+GE | Yield_Mg_ha_weight | NEH3_2014 | 0.385 | 0.488 | 0.327 | 0.789 | 1.493 | 1.177 |
| E+G+GE | Yield_Mg_ha_weight | NYH1_2014 | 6.280 | 5.034 | 5.178 | 1.248 | 0.972 | 1.213 |
| E+G+GE | Yield_Mg_ha_weight | TXH2_2014 | 2.534 | 0.068 | 0.228 | 37.042 | 0.300 | 11.113 |
| E+G+GE | Yield_Mg_ha_weight | WIH1_2014 | 0.658 | 1.015 | 0.571 | 0.648 | 1.779 | 1.153 |
| E+G+GE | Yield_Mg_ha_weight | Across | - | - | - | 4.794 | 2.285 | 2.333 |

**Table S2.** The prediction performance and the relative efficiency (RE) for **G2F_2014 dataset** in terms of mean squared error (MSE) for each Environment and for each trait, for the predictors E+G+BRR and E+G+GE+BRR under three different techniques to compute the Kernel for the effect of the Environment: without Environmental Covariates (No.EC), using Environmental covariates (EC) and using Environmental Covariates with Feature Engineering (FE).

| Predictor | Trait | Env | No.EC | EC | FE | NoEC_vs_EC | EC_vs_FE | NoEC_vs_FE |
| --- | --- | --- | --- | --- | --- | --- | --- | --- |
| E+G+BRR | Grain_Moisture_BLUE | DEH1_2014 | 1.190 | 19.827 | 5.703 | 0.060 | 3.477 | 0.209 |
| E+G+BRR | Grain_Moisture_BLUE | IAH1ab_2014 | 19.039 | 4.443 | 5.902 | 4.285 | 0.753 | 3.226 |
| E+G+BRR | Grain_Moisture_BLUE | IAH1c_2014 | 1.258 | 1.262 | 1.164 | 0.997 | 1.084 | 1.080 |
| E+G+BRR | Grain_Moisture_BLUE | IAH2_2014 | 20.289 | 1.609 | 1.327 | 12.614 | 1.212 | 15.288 |
| E+G+BRR | Grain_Moisture_BLUE | IAH3_2014 | 1.086 | 1.999 | 1.106 | 0.543 | 1.808 | 0.982 |
| E+G+BRR | Grain_Moisture_BLUE | IAH4_2014 | 1.296 | 10.487 | 1.990 | 0.124 | 5.269 | 0.651 |
| E+G+BRR | Grain_Moisture_BLUE | ILH1_2014 | 1.409 | 1.705 | 17.575 | 0.826 | 0.097 | 0.080 |
| E+G+BRR | Grain_Moisture_BLUE | INH1_2014 | 2.748 | 12.064 | 2.878 | 0.228 | 4.192 | 0.955 |
| E+G+BRR | Grain_Moisture_BLUE | MNH1_2014 | 114.022 | 20.368 | 39.311 | 5.598 | 0.518 | 2.901 |
| E+G+BRR | Grain_Moisture_BLUE | MOH1_2014 | 3.164 | 4.545 | 3.027 | 0.696 | 1.502 | 1.045 |
| E+G+BRR | Grain_Moisture_BLUE | MOH2_2014 | 59.809 | 10.701 | 24.879 | 5.589 | 0.430 | 2.404 |
| E+G+BRR | Grain_Moisture_BLUE | NCH1_2014 | 21.863 | 2.628 | 5.322 | 8.320 | 0.494 | 4.108 |
| E+G+BRR | Grain_Moisture_BLUE | NEH1_2014 | 9.572 | 7.726 | 6.582 | 1.239 | 1.174 | 1.454 |
| E+G+BRR | Grain_Moisture_BLUE | NEH2_2014 | 4.292 | 16.914 | 4.294 | 0.254 | 3.939 | 1.000 |
| E+G+BRR | Grain_Moisture_BLUE | NEH3_2014 | 6.397 | 17.586 | 7.522 | 0.364 | 2.338 | 0.851 |
| E+G+BRR | Grain_Moisture_BLUE | NYH1_2014 | 5.044 | 2.476 | 1.439 | 2.037 | 1.721 | 3.504 |
| E+G+BRR | Grain_Moisture_BLUE | TXH2_2014 | 30.572 | 184.131 | 2.608 | 0.166 | 70.597 | 11.722 |
| E+G+BRR | Grain_Moisture_BLUE | WIH1_2014 | 16.297 | 5.612 | 8.649 | 2.904 | 0.649 | 1.884 |
| E+G+BRR | Grain_Moisture_BLUE | Across | - | - | - | 2.602 | 5.625 | 2.964 |
| E+G+BRR | Grain_Moisture_weight | DEH1_2014 | 22.520 | 711.622 | 36.134 | 0.032 | 19.694 | 0.623 |
| E+G+BRR | Grain_Moisture_weight | IAH1ab_2014 | 18.024 | 3.070 | 3.884 | 5.871 | 0.790 | 4.640 |
| E+G+BRR | Grain_Moisture_weight | IAH1c_2014 | 17.107 | 1348.576 | 15.111 | 0.013 | 89.243 | 1.132 |
| E+G+BRR | Grain_Moisture_weight | IAH2_2014 | 23.359 | 137.197 | 0.783 | 0.170 | 175.220 | 29.833 |
| E+G+BRR | Grain_Moisture_weight | IAH3_2014 | 18.307 | 7.129 | 20.782 | 2.568 | 0.343 | 0.881 |
| E+G+BRR | Grain_Moisture_weight | IAH4_2014 | 3.554 | 91.941 | 1.982 | 0.039 | 46.385 | 1.793 |
| E+G+BRR | Grain_Moisture_weight | ILH1_2014 | 27.737 | 15.611 | 3.253 | 1.777 | 4.800 | 8.528 |
| E+G+BRR | Grain_Moisture_weight | INH1_2014 | 26.397 | 113.023 | 18.067 | 0.234 | 6.256 | 1.461 |
| E+G+BRR | Grain_Moisture_weight | MNH1_2014 | 49.919 | 363.880 | 6.290 | 0.137 | 57.847 | 7.936 |
| E+G+BRR | Grain_Moisture_weight | MOH1_2014 | 13.044 | 862.131 | 34.997 | 0.015 | 24.634 | 0.373 |
| E+G+BRR | Grain_Moisture_weight | MOH2_2014 | 3085.779 | 2692.357 | 2520.870 | 1.146 | 1.068 | 1.224 |
| E+G+BRR | Grain_Moisture_weight | NCH1_2014 | 1.903 | 149.510 | 2.184 | 0.013 | 68.460 | 0.871 |
| E+G+BRR | Grain_Moisture_weight | NEH1_2014 | 18.198 | 1293.071 | 6.893 | 0.014 | 187.603 | 2.640 |
| E+G+BRR | Grain_Moisture_weight | NEH2_2014 | 17.497 | 200.281 | 0.521 | 0.087 | 384.786 | 33.616 |
| E+G+BRR | Grain_Moisture_weight | NEH3_2014 | 61.654 | 321.899 | 2.024 | 0.192 | 159.018 | 30.457 |
| E+G+BRR | Grain_Moisture_weight | NYH1_2014 | 21.524 | 63.690 | 3.475 | 0.338 | 18.328 | 6.194 |
| E+G+BRR | Grain_Moisture_weight | TXH2_2014 | 17.256 | 902.032 | 20.005 | 0.019 | 45.090 | 0.863 |
| E+G+BRR | Grain_Moisture_weight | WIH1_2014 | 26.520 | 170.866 | 2.696 | 0.155 | 63.387 | 9.838 |
| E+G+BRR | Grain_Moisture_weight | Across | - | - | - | 0.712 | 75.164 | 7.939 |
| E+G+BRR | Yield_Mg_ha_BLUE | DEH1_2014 | 2.361 | 13.734 | 3.082 | 0.172 | 4.456 | 0.766 |
| E+G+BRR | Yield_Mg_ha_BLUE | IAH1ab_2014 | 5.568 | 2.855 | 1.976 | 1.950 | 1.445 | 2.818 |
| E+G+BRR | Yield_Mg_ha_BLUE | IAH1c_2014 | 1.677 | 2.021 | 5.148 | 0.830 | 0.393 | 0.326 |
| E+G+BRR | Yield_Mg_ha_BLUE | IAH2_2014 | 5.476 | 1.857 | 1.863 | 2.950 | 0.997 | 2.940 |
| E+G+BRR | Yield_Mg_ha_BLUE | IAH3_2014 | 20.378 | 3.238 | 4.054 | 6.293 | 0.799 | 5.027 |
| E+G+BRR | Yield_Mg_ha_BLUE | IAH4_2014 | 3.651 | 7.978 | 2.831 | 0.458 | 2.818 | 1.290 |
| E+G+BRR | Yield_Mg_ha_BLUE | ILH1_2014 | 8.269 | 5.979 | 8.696 | 1.383 | 0.688 | 0.951 |
| E+G+BRR | Yield_Mg_ha_BLUE | INH1_2014 | 3.560 | 1.748 | 2.123 | 2.036 | 0.824 | 1.677 |
| E+G+BRR | Yield_Mg_ha_BLUE | MNH1_2014 | 5.844 | 3.526 | 10.979 | 1.658 | 0.321 | 0.532 |
| E+G+BRR | Yield_Mg_ha_BLUE | MOH1_2014 | 4.122 | 8.255 | 2.088 | 0.499 | 3.953 | 1.974 |
| E+G+BRR | Yield_Mg_ha_BLUE | MOH2_2014 | 2.528 | 3.193 | 4.363 | 0.792 | 0.732 | 0.579 |
| E+G+BRR | Yield_Mg_ha_BLUE | NCH1_2014 | 8.638 | 10.473 | 5.392 | 0.825 | 1.943 | 1.602 |
| E+G+BRR | Yield_Mg_ha_BLUE | NEH1_2014 | 12.015 | 3.254 | 4.626 | 3.692 | 0.704 | 2.598 |
| E+G+BRR | Yield_Mg_ha_BLUE | NEH2_2014 | 11.924 | 2.718 | 2.780 | 4.387 | 0.978 | 4.289 |
| E+G+BRR | Yield_Mg_ha_BLUE | NEH3_2014 | 9.545 | 2.258 | 6.565 | 4.228 | 0.344 | 1.454 |
| E+G+BRR | Yield_Mg_ha_BLUE | NYH1_2014 | 1.488 | 180.148 | 8.639 | 0.008 | 20.853 | 0.172 |
| E+G+BRR | Yield_Mg_ha_BLUE | TXH2_2014 | 10.679 | 8.352 | 3.665 | 1.279 | 2.279 | 2.914 |
| E+G+BRR | Yield_Mg_ha_BLUE | WIH1_2014 | 8.269 | 2.706 | 8.081 | 3.056 | 0.335 | 1.023 |
| E+G+BRR | Yield_Mg_ha_BLUE | Across | - | - | - | 2.028 | 2.492 | 1.830 |
| E+G+BRR | Yield_Mg_ha_weight | DEH1_2014 | 0.388 | 11.139 | 1.378 | 0.035 | 8.084 | 0.282 |
| E+G+BRR | Yield_Mg_ha_weight | IAH1ab_2014 | 0.706 | 0.996 | 0.624 | 0.709 | 1.598 | 1.132 |
| E+G+BRR | Yield_Mg_ha_weight | IAH1c_2014 | 1.179 | 1.003 | 1.073 | 1.175 | 0.935 | 1.099 |
| E+G+BRR | Yield_Mg_ha_weight | IAH2_2014 | 0.145 | 0.728 | 0.145 | 0.199 | 5.037 | 1.000 |
| E+G+BRR | Yield_Mg_ha_weight | IAH3_2014 | 0.187 | 1.606 | 0.428 | 0.117 | 3.751 | 0.438 |
| E+G+BRR | Yield_Mg_ha_weight | IAH4_2014 | 0.211 | 0.486 | 0.328 | 0.435 | 1.479 | 0.644 |
| E+G+BRR | Yield_Mg_ha_weight | ILH1_2014 | 1.621 | 1.539 | 0.401 | 1.053 | 3.837 | 4.041 |
| E+G+BRR | Yield_Mg_ha_weight | INH1_2014 | 2.246 | 1.003 | 0.660 | 2.239 | 1.522 | 3.406 |
| E+G+BRR | Yield_Mg_ha_weight | MNH1_2014 | 0.399 | 0.384 | 0.409 | 1.038 | 0.939 | 0.974 |
| E+G+BRR | Yield_Mg_ha_weight | MOH1_2014 | 0.989 | 0.382 | 0.213 | 2.592 | 1.795 | 4.654 |
| E+G+BRR | Yield_Mg_ha_weight | MOH2_2014 | 0.958 | 0.487 | 0.503 | 1.967 | 0.970 | 1.907 |
| E+G+BRR | Yield_Mg_ha_weight | NCH1_2014 | 3.087 | 5.887 | 4.853 | 0.524 | 1.213 | 0.636 |
| E+G+BRR | Yield_Mg_ha_weight | NEH1_2014 | 0.848 | 0.062 | 0.215 | 13.655 | 0.289 | 3.944 |
| E+G+BRR | Yield_Mg_ha_weight | NEH2_2014 | 1.274 | 0.540 | 0.175 | 2.360 | 3.085 | 7.279 |
| E+G+BRR | Yield_Mg_ha_weight | NEH3_2014 | 0.361 | 0.975 | 0.405 | 0.370 | 2.409 | 0.891 |
| E+G+BRR | Yield_Mg_ha_weight | NYH1_2014 | 5.519 | 4.679 | 5.125 | 1.180 | 0.913 | 1.077 |
| E+G+BRR | Yield_Mg_ha_weight | TXH2_2014 | 0.997 | 0.190 | 0.300 | 5.245 | 0.634 | 3.324 |
| E+G+BRR | Yield_Mg_ha_weight | WIH1_2014 | 0.902 | 1.001 | 1.121 | 0.901 | 0.893 | 0.805 |
| E+G+BRR | Yield_Mg_ha_weight | Across | - | - | - | 1.989 | 2.188 | 2.085 |
| E+G+GE+BRR | Grain_Moisture_BLUE | DEH1_2014 | 1.176 | 28.570 | 3.478 | 0.041 | 8.215 | 0.338 |
| E+G+GE+BRR | Grain_Moisture_BLUE | IAH1ab_2014 | 21.293 | 3.704 | 4.089 | 5.750 | 0.906 | 5.208 |
| E+G+GE+BRR | Grain_Moisture_BLUE | IAH1c_2014 | 13.583 | 1.231 | 1.076 | 11.036 | 1.144 | 12.630 |
| E+G+GE+BRR | Grain_Moisture_BLUE | IAH2_2014 | 7.255 | 0.955 | 0.844 | 7.595 | 1.131 | 8.592 |
| E+G+GE+BRR | Grain_Moisture_BLUE | IAH3_2014 | 3.085 | 1.571 | 0.767 | 1.964 | 2.049 | 4.025 |
| E+G+GE+BRR | Grain_Moisture_BLUE | IAH4_2014 | 1.197 | 9.713 | 2.717 | 0.123 | 3.575 | 0.441 |
| E+G+GE+BRR | Grain_Moisture_BLUE | ILH1_2014 | 3.839 | 1.702 | 11.608 | 2.256 | 0.147 | 0.331 |
| E+G+GE+BRR | Grain_Moisture_BLUE | INH1_2014 | 3.230 | 2.763 | 6.758 | 1.169 | 0.409 | 0.478 |
| E+G+GE+BRR | Grain_Moisture_BLUE | MNH1_2014 | 73.658 | 25.267 | 48.188 | 2.915 | 0.524 | 1.529 |
| E+G+GE+BRR | Grain_Moisture_BLUE | MOH1_2014 | 10.349 | 1.976 | 2.545 | 5.239 | 0.776 | 4.066 |
| E+G+GE+BRR | Grain_Moisture_BLUE | MOH2_2014 | 55.131 | 6.937 | 20.602 | 7.948 | 0.337 | 2.676 |
| E+G+GE+BRR | Grain_Moisture_BLUE | NCH1_2014 | 29.901 | 1.861 | 9.045 | 16.064 | 0.206 | 3.306 |
| E+G+GE+BRR | Grain_Moisture_BLUE | NEH1_2014 | 11.048 | 3.800 | 6.058 | 2.907 | 0.627 | 1.824 |
| E+G+GE+BRR | Grain_Moisture_BLUE | NEH2_2014 | 4.302 | 24.797 | 4.180 | 0.174 | 5.932 | 1.029 |
| E+G+GE+BRR | Grain_Moisture_BLUE | NEH3_2014 | 3.418 | 6.122 | 7.733 | 0.558 | 0.792 | 0.442 |
| E+G+GE+BRR | Grain_Moisture_BLUE | NYH1_2014 | 3.699 | 2.576 | 1.758 | 1.436 | 1.465 | 2.104 |
| E+G+GE+BRR | Grain_Moisture_BLUE | TXH2_2014 | 16.714 | 73.082 | 3.674 | 0.229 | 19.890 | 4.549 |
| E+G+GE+BRR | Grain_Moisture_BLUE | WIH1_2014 | 18.051 | 4.943 | 6.483 | 3.652 | 0.762 | 2.784 |
| E+G+GE+BRR | Grain_Moisture_BLUE | Across | - | - | - | 3.948 | 2.716 | 3.131 |
| E+G+GE+BRR | Grain_Moisture_weight | DEH1_2014 | 10.659 | 278.019 | 67.259 | 0.038 | 4.134 | 0.159 |
| E+G+GE+BRR | Grain_Moisture_weight | IAH1ab_2014 | 22.067 | 2.760 | 3.304 | 7.995 | 0.835 | 6.678 |
| E+G+GE+BRR | Grain_Moisture_weight | IAH1c_2014 | 11.930 | 828.801 | 13.936 | 0.014 | 59.474 | 0.856 |
| E+G+GE+BRR | Grain_Moisture_weight | IAH2_2014 | 8.238 | 38.083 | 0.817 | 0.216 | 46.642 | 10.089 |
| E+G+GE+BRR | Grain_Moisture_weight | IAH3_2014 | 9.865 | 102.256 | 24.160 | 0.097 | 4.232 | 0.408 |
| E+G+GE+BRR | Grain_Moisture_weight | IAH4_2014 | 13.934 | 2.519 | 2.016 | 5.532 | 1.250 | 6.913 |
| E+G+GE+BRR | Grain_Moisture_weight | ILH1_2014 | 15.045 | 464.441 | 42.684 | 0.032 | 10.881 | 0.353 |
| E+G+GE+BRR | Grain_Moisture_weight | INH1_2014 | 25.821 | 3.311 | 6.853 | 7.798 | 0.483 | 3.768 |
| E+G+GE+BRR | Grain_Moisture_weight | MNH1_2014 | 35.611 | 303.848 | 3.233 | 0.117 | 93.989 | 11.016 |
| E+G+GE+BRR | Grain_Moisture_weight | MOH1_2014 | 22.565 | 824.200 | 22.806 | 0.027 | 36.139 | 0.989 |
| E+G+GE+BRR | Grain_Moisture_weight | MOH2_2014 | 2947.225 | 2739.022 | 2605.419 | 1.076 | 1.051 | 1.131 |
| E+G+GE+BRR | Grain_Moisture_weight | NCH1_2014 | 2.367 | 4.940 | 3.326 | 0.479 | 1.485 | 0.712 |
| E+G+GE+BRR | Grain_Moisture_weight | NEH1_2014 | 39.278 | 110.188 | 0.049 | 0.357 | 2253.335 | 803.237 |
| E+G+GE+BRR | Grain_Moisture_weight | NEH2_2014 | 27.701 | 15.478 | 0.065 | 1.790 | 236.665 | 423.557 |
| E+G+GE+BRR | Grain_Moisture_weight | NEH3_2014 | 70.926 | 43.751 | 0.895 | 1.621 | 48.873 | 79.229 |
| E+G+GE+BRR | Grain_Moisture_weight | NYH1_2014 | 16.284 | 142.399 | 10.356 | 0.114 | 13.751 | 1.573 |
| E+G+GE+BRR | Grain_Moisture_weight | TXH2_2014 | 48.764 | 850.617 | 11.546 | 0.057 | 73.669 | 4.223 |
| E+G+GE+BRR | Grain_Moisture_weight | WIH1_2014 | 30.889 | 162.312 | 2.108 | 0.190 | 77.002 | 14.654 |
| E+G+GE+BRR | Grain_Moisture_weight | Across | - | - | - | 1.531 | 164.661 | 76.086 |
| E+G+GE+BRR | Yield_Mg_ha_BLUE | DEH1_2014 | 5.741 | 9.006 | 5.192 | 0.638 | 1.734 | 1.106 |
| E+G+GE+BRR | Yield_Mg_ha_BLUE | IAH1ab_2014 | 3.587 | 2.498 | 1.990 | 1.436 | 1.255 | 1.803 |
| E+G+GE+BRR | Yield_Mg_ha_BLUE | IAH1c_2014 | 1.578 | 1.433 | 3.485 | 1.101 | 0.411 | 0.453 |
| E+G+GE+BRR | Yield_Mg_ha_BLUE | IAH2_2014 | 3.520 | 1.764 | 2.119 | 1.996 | 0.832 | 1.661 |
| E+G+GE+BRR | Yield_Mg_ha_BLUE | IAH3_2014 | 12.825 | 6.906 | 5.115 | 1.857 | 1.350 | 2.508 |
| E+G+GE+BRR | Yield_Mg_ha_BLUE | IAH4_2014 | 9.901 | 8.285 | 2.406 | 1.195 | 3.443 | 4.115 |
| E+G+GE+BRR | Yield_Mg_ha_BLUE | ILH1_2014 | 6.462 | 4.563 | 6.156 | 1.416 | 0.741 | 1.050 |
| E+G+GE+BRR | Yield_Mg_ha_BLUE | INH1_2014 | 1.324 | 3.716 | 3.749 | 0.356 | 0.991 | 0.353 |
| E+G+GE+BRR | Yield_Mg_ha_BLUE | MNH1_2014 | 7.807 | 3.874 | 9.573 | 2.015 | 0.405 | 0.816 |
| E+G+GE+BRR | Yield_Mg_ha_BLUE | MOH1_2014 | 1.713 | 10.768 | 3.687 | 0.159 | 2.921 | 0.465 |
| E+G+GE+BRR | Yield_Mg_ha_BLUE | MOH2_2014 | 2.618 | 2.720 | 3.846 | 0.963 | 0.707 | 0.681 |
| E+G+GE+BRR | Yield_Mg_ha_BLUE | NCH1_2014 | 8.503 | 12.824 | 10.559 | 0.663 | 1.215 | 0.805 |
| E+G+GE+BRR | Yield_Mg_ha_BLUE | NEH1_2014 | 9.048 | 2.313 | 4.317 | 3.913 | 0.536 | 2.096 |
| E+G+GE+BRR | Yield_Mg_ha_BLUE | NEH2_2014 | 10.312 | 2.477 | 3.055 | 4.163 | 0.811 | 3.375 |
| E+G+GE+BRR | Yield_Mg_ha_BLUE | NEH3_2014 | 10.025 | 1.892 | 1.717 | 5.299 | 1.102 | 5.840 |
| E+G+GE+BRR | Yield_Mg_ha_BLUE | NYH1_2014 | 1.779 | 185.311 | 7.777 | 0.010 | 23.828 | 0.229 |
| E+G+GE+BRR | Yield_Mg_ha_BLUE | TXH2_2014 | 3.449 | 15.599 | 2.904 | 0.221 | 5.372 | 1.188 |
| E+G+GE+BRR | Yield_Mg_ha_BLUE | WIH1_2014 | 8.653 | 2.623 | 7.932 | 3.300 | 0.331 | 1.091 |
| E+G+GE+BRR | Yield_Mg_ha_BLUE | Across | - | - | - | 1.706 | 2.666 | 1.646 |
| E+G+GE+BRR | Yield_Mg_ha_weight | DEH1_2014 | 0.601 | 11.480 | 1.330 | 0.052 | 8.629 | 0.452 |
| E+G+GE+BRR | Yield_Mg_ha_weight | IAH1ab_2014 | 0.489 | 0.926 | 0.630 | 0.528 | 1.469 | 0.775 |
| E+G+GE+BRR | Yield_Mg_ha_weight | IAH1c_2014 | 1.375 | 0.936 | 0.988 | 1.470 | 0.947 | 1.392 |
| E+G+GE+BRR | Yield_Mg_ha_weight | IAH2_2014 | 0.319 | 0.753 | 0.178 | 0.424 | 4.238 | 1.796 |
| E+G+GE+BRR | Yield_Mg_ha_weight | IAH3_2014 | 0.413 | 1.701 | 0.356 | 0.243 | 4.780 | 1.159 |
| E+G+GE+BRR | Yield_Mg_ha_weight | IAH4_2014 | 0.168 | 0.454 | 0.298 | 0.370 | 1.527 | 0.565 |
| E+G+GE+BRR | Yield_Mg_ha_weight | ILH1_2014 | 0.806 | 1.532 | 0.420 | 0.526 | 3.648 | 1.918 |
| E+G+GE+BRR | Yield_Mg_ha_weight | INH1_2014 | 0.753 | 1.015 | 0.717 | 0.742 | 1.416 | 1.050 |
| E+G+GE+BRR | Yield_Mg_ha_weight | MNH1_2014 | 1.927 | 0.442 | 0.416 | 4.359 | 1.062 | 4.630 |
| E+G+GE+BRR | Yield_Mg_ha_weight | MOH1_2014 | 0.202 | 0.381 | 0.172 | 0.531 | 2.207 | 1.172 |
| E+G+GE+BRR | Yield_Mg_ha_weight | MOH2_2014 | 0.112 | 0.479 | 0.312 | 0.235 | 1.536 | 0.360 |
| E+G+GE+BRR | Yield_Mg_ha_weight | NCH1_2014 | 6.787 | 8.497 | 5.633 | 0.799 | 1.508 | 1.205 |
| E+G+GE+BRR | Yield_Mg_ha_weight | NEH1_2014 | 1.080 | 0.037 | 0.176 | 29.113 | 0.211 | 6.154 |
| E+G+GE+BRR | Yield_Mg_ha_weight | NEH2_2014 | 1.191 | 0.495 | 0.143 | 2.406 | 3.471 | 8.353 |
| E+G+GE+BRR | Yield_Mg_ha_weight | NEH3_2014 | 0.385 | 0.401 | 0.326 | 0.958 | 1.231 | 1.179 |
| E+G+GE+BRR | Yield_Mg_ha_weight | NYH1_2014 | 6.280 | 5.295 | 5.016 | 1.186 | 1.056 | 1.252 |
| E+G+GE+BRR | Yield_Mg_ha_weight | TXH2_2014 | 2.534 | 0.229 | 0.528 | 11.064 | 0.434 | 4.797 |
| E+G+GE+BRR | Yield_Mg_ha_weight | WIH1_2014 | 0.658 | 0.998 | 0.936 | 0.659 | 1.066 | 0.703 |
| E+G+GE+BRR | Yield_Mg_ha_weight | Across | - | - | - | 3.092 | 2.246 | 2.162 |

**Table S3**. The prediction performance and the relative efficiency (RE) for **G2F_2015 dataset** in terms of mean squared error (MSE) for each Environment and for each trait, for the predictors E+G and E+G+GE under three different techniques to compute the Kernel for the effect of the Environment: without Environmental Covariates (No.EC), using Environmental covariates (EC) and using Environmental Covariates with Feature Engineering (FE).

| Predictor | Trait | Env | No.EC | EC | FE | NoEC_vs_EC | EC_vs_FE | NoEC_vs_FE |
| --- | --- | --- | --- | --- | --- | --- | --- | --- |
| E+G | Grain_Moisture_BLUE | DEH1_2015 | 10.930 | 13.369 | 10.881 | 0.818 | 1.229 | 1.005 |
| E+G | Grain_Moisture_BLUE | GAH1_2015 | 19.572 | 65.241 | 17.035 | 0.300 | 3.830 | 1.149 |
| E+G | Grain_Moisture_BLUE | INH1_2015 | 8.136 | 8.397 | 6.668 | 0.969 | 1.259 | 1.220 |
| E+G | Grain_Moisture_BLUE | MNH1_2015 | 1.106 | 2.318 | 1.133 | 0.477 | 2.046 | 0.976 |
| E+G | Grain_Moisture_BLUE | NCH1_2015 | 0.925 | 1.134 | 10.223 | 0.815 | 0.111 | 0.091 |
| E+G | Grain_Moisture_BLUE | NEH1_NEH4_2015 | 5.789 | 10.118 | 1.660 | 0.572 | 6.094 | 3.487 |
| E+G | Grain_Moisture_BLUE | NEH2_2015 | 10.138 | 29.154 | 9.776 | 0.348 | 2.982 | 1.037 |
| E+G | Grain_Moisture_BLUE | NEH3_2015 | 5.711 | 10.169 | 5.748 | 0.562 | 1.769 | 0.994 |
| E+G | Grain_Moisture_BLUE | NYH2_2015 | 1.617 | 1.447 | 1.144 | 1.118 | 1.265 | 1.414 |
| E+G | Grain_Moisture_BLUE | NYH3_2015 | 15.515 | 38.550 | 9.283 | 0.403 | 4.153 | 1.671 |
| E+G | Grain_Moisture_BLUE | OHH1_2015 | 3.056 | 4.878 | 2.832 | 0.627 | 1.723 | 1.079 |
| E+G | Grain_Moisture_BLUE | SDH1_2015 | 16.877 | 32.928 | 15.667 | 0.513 | 2.102 | 1.077 |
| E+G | Grain_Moisture_BLUE | Across | - | - | - | 0.627 | 2.380 | 1.267 |
| E+G | Grain_Moisture_weight | DEH1_2015 | 43.744 | 47.543 | 14.544 | 0.920 | 3.269 | 3.008 |
| E+G | Grain_Moisture_weight | GAH1_2015 | 190.772 | 65.716 | 105.604 | 2.903 | 0.622 | 1.807 |
| E+G | Grain_Moisture_weight | INH1_2015 | 15.393 | 12.789 | 5.914 | 1.204 | 2.163 | 2.603 |
| E+G | Grain_Moisture_weight | MNH1_2015 | 14.974 | 7.680 | 7.038 | 1.950 | 1.091 | 2.128 |
| E+G | Grain_Moisture_weight | NCH1_2015 | 53.832 | 27.789 | 36.930 | 1.937 | 0.753 | 1.458 |
| E+G | Grain_Moisture_weight | NEH1_NEH4_2015 | 5.515 | 17.372 | 3.542 | 0.318 | 4.905 | 1.557 |
| E+G | Grain_Moisture_weight | NEH2_2015 | 12.741 | 0.337 | 0.356 | 37.808 | 0.948 | 35.830 |
| E+G | Grain_Moisture_weight | NEH3_2015 | 2.195 | 2.114 | 2.628 | 1.038 | 0.805 | 0.835 |
| E+G | Grain_Moisture_weight | NYH2_2015 | 17.474 | 15.356 | 0.724 | 1.138 | 21.201 | 24.126 |
| E+G | Grain_Moisture_weight | NYH3_2015 | 41.134 | 12.575 | 3.303 | 3.271 | 3.807 | 12.453 |
| E+G | Grain_Moisture_weight | OHH1_2015 | 1.753 | 0.343 | 0.551 | 5.111 | 0.623 | 3.182 |
| E+G | Grain_Moisture_weight | SDH1_2015 | 10.138 | 1.484 | 4.604 | 6.831 | 0.322 | 2.202 |
| E+G | Grain_Moisture_weight | Across | - | - | - | 5.369 | 3.376 | 7.599 |
| E+G | Yield_Mg_ha_BLUE | DEH1_2015 | 15.270 | 7.328 | 11.487 | 2.084 | 0.638 | 1.329 |
| E+G | Yield_Mg_ha_BLUE | GAH1_2015 | 2.737 | 4.108 | 4.904 | 0.666 | 0.838 | 0.558 |
| E+G | Yield_Mg_ha_BLUE | INH1_2015 | 3.216 | 2.689 | 2.591 | 1.196 | 1.038 | 1.241 |
| E+G | Yield_Mg_ha_BLUE | MNH1_2015 | 3.886 | 2.551 | 7.027 | 1.523 | 0.363 | 0.553 |
| E+G | Yield_Mg_ha_BLUE | NCH1_2015 | 5.495 | 3.247 | 3.825 | 1.693 | 0.849 | 1.437 |
| E+G | Yield_Mg_ha_BLUE | NEH1_NEH4_2015 | 5.358 | 11.826 | 9.450 | 0.453 | 1.251 | 0.567 |
| E+G | Yield_Mg_ha_BLUE | NEH2_2015 | 1.777 | 2.264 | 2.498 | 0.785 | 0.906 | 0.711 |
| E+G | Yield_Mg_ha_BLUE | NEH3_2015 | 3.797 | 3.489 | 1.759 | 1.088 | 1.984 | 2.159 |
| E+G | Yield_Mg_ha_BLUE | NYH2_2015 | 4.071 | 4.064 | 3.787 | 1.002 | 1.073 | 1.075 |
| E+G | Yield_Mg_ha_BLUE | NYH3_2015 | 3.704 | 3.604 | 3.882 | 1.028 | 0.928 | 0.954 |
| E+G | Yield_Mg_ha_BLUE | OHH1_2015 | 2.448 | 2.258 | 3.911 | 1.084 | 0.577 | 0.626 |
| E+G | Yield_Mg_ha_BLUE | SDH1_2015 | 12.148 | 10.279 | 11.153 | 1.182 | 0.922 | 1.089 |
| E+G | Yield_Mg_ha_BLUE | Across | - | - | - | 1.149 | 0.947 | 1.025 |
| E+G | Yield_Mg_ha_weight | DEH1_2015 | 0.198 | 0.048 | 0.111 | 4.098 | 0.434 | 1.778 |
| E+G | Yield_Mg_ha_weight | GAH1_2015 | 0.328 | 0.598 | 0.443 | 0.549 | 1.348 | 0.740 |
| E+G | Yield_Mg_ha_weight | INH1_2015 | 0.221 | 1.347 | 0.632 | 0.164 | 2.131 | 0.350 |
| E+G | Yield_Mg_ha_weight | MNH1_2015 | 0.251 | 0.799 | 0.595 | 0.315 | 1.343 | 0.422 |
| E+G | Yield_Mg_ha_weight | NCH1_2015 | 0.149 | 0.167 | 0.100 | 0.891 | 1.675 | 1.493 |
| E+G | Yield_Mg_ha_weight | NEH1_NEH4_2015 | 0.238 | 0.176 | 0.567 | 1.355 | 0.310 | 0.420 |
| E+G | Yield_Mg_ha_weight | NEH2_2015 | 0.076 | 0.073 | 0.061 | 1.042 | 1.208 | 1.260 |
| E+G | Yield_Mg_ha_weight | NEH3_2015 | 0.141 | 0.434 | 0.438 | 0.326 | 0.990 | 0.323 |
| E+G | Yield_Mg_ha_weight | NYH2_2015 | 0.139 | 0.247 | 0.138 | 0.562 | 1.786 | 1.003 |
| E+G | Yield_Mg_ha_weight | NYH3_2015 | 0.482 | 0.268 | 0.423 | 1.802 | 0.632 | 1.139 |
| E+G | Yield_Mg_ha_weight | OHH1_2015 | 2.903 | 2.771 | 3.221 | 1.048 | 0.860 | 0.901 |
| E+G | Yield_Mg_ha_weight | SDH1_2015 | 0.090 | 1.141 | 0.095 | 0.079 | 11.959 | 0.944 |
| E+G | Yield_Mg_ha_weight | Across | - | - | - | 1.019 | 2.056 | 0.898 |
| E+G+GE | Grain_Moisture_BLUE | DEH1_2015 | 8.293 | 13.932 | 14.053 | 0.595 | 0.991 | 0.590 |
| E+G+GE | Grain_Moisture_BLUE | GAH1_2015 | 17.403 | 68.494 | 6.412 | 0.254 | 10.682 | 2.714 |
| E+G+GE | Grain_Moisture_BLUE | INH1_2015 | 2.106 | 5.427 | 4.064 | 0.388 | 1.336 | 0.518 |
| E+G+GE | Grain_Moisture_BLUE | MNH1_2015 | 1.919 | 2.196 | 1.103 | 0.874 | 1.991 | 1.740 |
| E+G+GE | Grain_Moisture_BLUE | NCH1_2015 | 1.200 | 1.122 | 20.096 | 1.070 | 0.056 | 0.060 |
| E+G+GE | Grain_Moisture_BLUE | NEH1_NEH4_2015 | 4.308 | 11.605 | 1.717 | 0.371 | 6.761 | 2.510 |
| E+G+GE | Grain_Moisture_BLUE | NEH2_2015 | 9.531 | 41.591 | 14.812 | 0.229 | 2.808 | 0.644 |
| E+G+GE | Grain_Moisture_BLUE | NEH3_2015 | 4.615 | 8.211 | 5.280 | 0.562 | 1.555 | 0.874 |
| E+G+GE | Grain_Moisture_BLUE | NYH2_2015 | 3.198 | 1.178 | 1.891 | 2.715 | 0.623 | 1.691 |
| E+G+GE | Grain_Moisture_BLUE | NYH3_2015 | 9.145 | 42.347 | 11.048 | 0.216 | 3.833 | 0.828 |
| E+G+GE | Grain_Moisture_BLUE | OHH1_2015 | 4.407 | 3.728 | 3.128 | 1.182 | 1.192 | 1.409 |
| E+G+GE | Grain_Moisture_BLUE | SDH1_2015 | 14.536 | 25.103 | 15.193 | 0.579 | 1.652 | 0.957 |
| E+G+GE | Grain_Moisture_BLUE | Across | - | - | - | 0.753 | 2.790 | 1.211 |
| E+G+GE | Grain_Moisture_weight | DEH1_2015 | 1.208 | 25.929 | 4.617 | 0.047 | 5.616 | 0.262 |
| E+G+GE | Grain_Moisture_weight | GAH1_2015 | 142.447 | 53.322 | 84.860 | 2.672 | 0.628 | 1.679 |
| E+G+GE | Grain_Moisture_weight | INH1_2015 | 28.465 | 6.267 | 0.731 | 4.542 | 8.569 | 38.924 |
| E+G+GE | Grain_Moisture_weight | MNH1_2015 | 11.750 | 2.744 | 4.886 | 4.282 | 0.562 | 2.405 |
| E+G+GE | Grain_Moisture_weight | NCH1_2015 | 207.235 | 37.988 | 62.029 | 5.455 | 0.612 | 3.341 |
| E+G+GE | Grain_Moisture_weight | NEH1_NEH4_2015 | 12.290 | 8.329 | 2.639 | 1.476 | 3.156 | 4.657 |
| E+G+GE | Grain_Moisture_weight | NEH2_2015 | 0.262 | 0.793 | 1.024 | 0.330 | 0.775 | 0.256 |
| E+G+GE | Grain_Moisture_weight | NEH3_2015 | 88.155 | 7.651 | 4.064 | 11.522 | 1.883 | 21.693 |
| E+G+GE | Grain_Moisture_weight | NYH2_2015 | 7.591 | 9.741 | 2.831 | 0.779 | 3.441 | 2.681 |
| E+G+GE | Grain_Moisture_weight | NYH3_2015 | 2.786 | 16.958 | 3.725 | 0.164 | 4.553 | 0.748 |
| E+G+GE | Grain_Moisture_weight | OHH1_2015 | 42.678 | 0.438 | 0.515 | 97.372 | 0.852 | 82.919 |
| E+G+GE | Grain_Moisture_weight | SDH1_2015 | 61.817 | 1.939 | 6.588 | 31.874 | 0.294 | 9.384 |
| E+G+GE | Grain_Moisture_weight | Across | - | - | - | 13.376 | 2.578 | 14.079 |
| E+G+GE | Yield_Mg_ha_BLUE | DEH1_2015 | 12.318 | 6.861 | 11.244 | 1.795 | 0.610 | 1.096 |
| E+G+GE | Yield_Mg_ha_BLUE | GAH1_2015 | 2.833 | 5.106 | 4.628 | 0.555 | 1.103 | 0.612 |
| E+G+GE | Yield_Mg_ha_BLUE | INH1_2015 | 2.710 | 2.687 | 2.598 | 1.009 | 1.034 | 1.043 |
| E+G+GE | Yield_Mg_ha_BLUE | MNH1_2015 | 7.126 | 2.487 | 7.481 | 2.866 | 0.333 | 0.953 |
| E+G+GE | Yield_Mg_ha_BLUE | NCH1_2015 | 4.574 | 3.163 | 3.601 | 1.446 | 0.878 | 1.270 |
| E+G+GE | Yield_Mg_ha_BLUE | NEH1_NEH4_2015 | 5.144 | 12.160 | 9.067 | 0.423 | 1.341 | 0.567 |
| E+G+GE | Yield_Mg_ha_BLUE | NEH2_2015 | 1.739 | 2.865 | 2.993 | 0.607 | 0.957 | 0.581 |
| E+G+GE | Yield_Mg_ha_BLUE | NEH3_2015 | 1.760 | 3.716 | 1.688 | 0.474 | 2.202 | 1.043 |
| E+G+GE | Yield_Mg_ha_BLUE | NYH2_2015 | 5.339 | 3.762 | 3.451 | 1.419 | 1.090 | 1.547 |
| E+G+GE | Yield_Mg_ha_BLUE | NYH3_2015 | 3.612 | 3.520 | 3.633 | 1.026 | 0.969 | 0.994 |
| E+G+GE | Yield_Mg_ha_BLUE | OHH1_2015 | 2.447 | 2.631 | 3.458 | 0.930 | 0.761 | 0.708 |
| E+G+GE | Yield_Mg_ha_BLUE | SDH1_2015 | 11.541 | 10.664 | 11.967 | 1.082 | 0.891 | 0.964 |
| E+G+GE | Yield_Mg_ha_BLUE | Across | - | - | - | 1.136 | 1.014 | 0.948 |
| E+G+GE | Yield_Mg_ha_weight | DEH1_2015 | 0.062 | 0.066 | 0.050 | 0.935 | 1.331 | 1.244 |
| E+G+GE | Yield_Mg_ha_weight | GAH1_2015 | 0.430 | 0.325 | 0.366 | 1.324 | 0.887 | 1.174 |
| E+G+GE | Yield_Mg_ha_weight | INH1_2015 | 0.225 | 1.312 | 0.225 | 0.172 | 5.836 | 1.002 |
| E+G+GE | Yield_Mg_ha_weight | MNH1_2015 | 0.168 | 0.651 | 0.541 | 0.258 | 1.203 | 0.311 |
| E+G+GE | Yield_Mg_ha_weight | NCH1_2015 | 0.271 | 0.071 | 0.112 | 3.844 | 0.631 | 2.426 |
| E+G+GE | Yield_Mg_ha_weight | NEH1_NEH4_2015 | 0.347 | 0.559 | 0.738 | 0.621 | 0.758 | 0.470 |
| E+G+GE | Yield_Mg_ha_weight | NEH2_2015 | 0.300 | 0.060 | 0.056 | 4.990 | 1.069 | 5.336 |
| E+G+GE | Yield_Mg_ha_weight | NEH3_2015 | 0.109 | 0.684 | 0.482 | 0.160 | 1.418 | 0.226 |
| E+G+GE | Yield_Mg_ha_weight | NYH2_2015 | 0.366 | 0.231 | 0.252 | 1.585 | 0.917 | 1.454 |
| E+G+GE | Yield_Mg_ha_weight | NYH3_2015 | 0.276 | 0.372 | 0.302 | 0.743 | 1.231 | 0.915 |
| E+G+GE | Yield_Mg_ha_weight | OHH1_2015 | 2.740 | 2.607 | 3.132 | 1.051 | 0.832 | 0.875 |
| E+G+GE | Yield_Mg_ha_weight | SDH1_2015 | 0.095 | 1.686 | 0.160 | 0.056 | 10.536 | 0.594 |
| E+G+GE | Yield_Mg_ha_weight | Across | - | - | - | 1.312 | 2.221 | 1.336 |
|  |  |  |  |  |  |  |  |  |

**Table S4**. The prediction performance and the relative efficiency (RE) for **G2F_2015** **dataset** in terms of mean squared error (MSE) for each Environment and for each trait, for the predictors E+G+BRR and E+G+GE+BRR under three different techniques to compute the Kernel for the effect of the Environment: without Environmental Covariates (No.EC), using Environmental covariates (EC) and using Environmental Covariates with Feature Engineering (FE).

| Predictor | Trait | Env | No.EC | EC | FE | NoEC_vs_EC | EC_vs_FE | NoEC_vs_FE |
| --- | --- | --- | --- | --- | --- | --- | --- | --- |
| E+G+BRR | Grain_Moisture_BLUE | DEH1_2015 | 10.930 | 13.355 | 5.231 | 0.818 | 2.553 | 2.090 |
| E+G+BRR | Grain_Moisture_BLUE | GAH1_2015 | 19.572 | 67.067 | 16.333 | 0.292 | 4.106 | 1.198 |
| E+G+BRR | Grain_Moisture_BLUE | INH1_2015 | 8.136 | 10.469 | 8.949 | 0.777 | 1.170 | 0.909 |
| E+G+BRR | Grain_Moisture_BLUE | MNH1_2015 | 1.106 | 1.819 | 1.425 | 0.608 | 1.277 | 0.776 |
| E+G+BRR | Grain_Moisture_BLUE | NCH1_2015 | 0.925 | 0.906 | 27.510 | 1.021 | 0.033 | 0.034 |
| E+G+BRR | Grain_Moisture_BLUE | NEH1_NEH4_2015 | 5.789 | 9.814 | 1.634 | 0.590 | 6.007 | 3.543 |
| E+G+BRR | Grain_Moisture_BLUE | NEH2_2015 | 10.138 | 29.612 | 8.281 | 0.342 | 3.576 | 1.224 |
| E+G+BRR | Grain_Moisture_BLUE | NEH3_2015 | 5.711 | 8.720 | 5.460 | 0.655 | 1.597 | 1.046 |
| E+G+BRR | Grain_Moisture_BLUE | NYH2_2015 | 1.617 | 1.247 | 1.497 | 1.297 | 0.833 | 1.080 |
| E+G+BRR | Grain_Moisture_BLUE | NYH3_2015 | 15.515 | 29.842 | 5.884 | 0.520 | 5.071 | 2.637 |
| E+G+BRR | Grain_Moisture_BLUE | OHH1_2015 | 3.056 | 3.838 | 2.360 | 0.796 | 1.626 | 1.295 |
| E+G+BRR | Grain_Moisture_BLUE | SDH1_2015 | 16.877 | 23.750 | 13.217 | 0.711 | 1.797 | 1.277 |
| E+G+BRR | Grain_Moisture_BLUE | Across | - | - | - | 0.702 | 2.471 | 1.426 |
| E+G+BRR | Grain_Moisture_weight | DEH1_2015 | 43.744 | 29.254 | 0.445 | 1.495 | 65.680 | 98.212 |
| E+G+BRR | Grain_Moisture_weight | GAH1_2015 | 190.772 | 71.741 | 162.806 | 2.659 | 0.441 | 1.172 |
| E+G+BRR | Grain_Moisture_weight | INH1_2015 | 15.393 | 4.963 | 1.782 | 3.102 | 2.786 | 8.640 |
| E+G+BRR | Grain_Moisture_weight | MNH1_2015 | 14.974 | 6.967 | 5.716 | 2.149 | 1.219 | 2.620 |
| E+G+BRR | Grain_Moisture_weight | NCH1_2015 | 53.832 | 27.344 | 68.472 | 1.969 | 0.399 | 0.786 |
| E+G+BRR | Grain_Moisture_weight | NEH1_NEH4_2015 | 5.515 | 16.439 | 3.552 | 0.336 | 4.628 | 1.553 |
| E+G+BRR | Grain_Moisture_weight | NEH2_2015 | 12.741 | 1.578 | 0.927 | 8.074 | 1.702 | 13.746 |
| E+G+BRR | Grain_Moisture_weight | NEH3_2015 | 2.195 | 3.500 | 2.466 | 0.627 | 1.419 | 0.890 |
| E+G+BRR | Grain_Moisture_weight | NYH2_2015 | 17.474 | 14.625 | 0.714 | 1.195 | 20.481 | 24.470 |
| E+G+BRR | Grain_Moisture_weight | NYH3_2015 | 41.134 | 13.601 | 1.966 | 3.024 | 6.918 | 20.921 |
| E+G+BRR | Grain_Moisture_weight | OHH1_2015 | 1.753 | 1.611 | 0.351 | 1.089 | 4.588 | 4.994 |
| E+G+BRR | Grain_Moisture_weight | SDH1_2015 | 10.138 | 1.350 | 3.093 | 7.510 | 0.436 | 3.277 |
| E+G+BRR | Grain_Moisture_weight | Across | - | - | - | 2.769 | 9.225 | 15.107 |
| E+G+BRR | Yield_Mg_ha_BLUE | DEH1_2015 | 15.270 | 7.063 | 11.567 | 2.162 | 0.611 | 1.320 |
| E+G+BRR | Yield_Mg_ha_BLUE | GAH1_2015 | 2.737 | 3.737 | 9.345 | 0.733 | 0.400 | 0.293 |
| E+G+BRR | Yield_Mg_ha_BLUE | INH1_2015 | 3.216 | 2.704 | 16.177 | 1.189 | 0.167 | 0.199 |
| E+G+BRR | Yield_Mg_ha_BLUE | MNH1_2015 | 3.886 | 2.376 | 7.154 | 1.636 | 0.332 | 0.543 |
| E+G+BRR | Yield_Mg_ha_BLUE | NCH1_2015 | 5.495 | 3.287 | 4.969 | 1.672 | 0.662 | 1.106 |
| E+G+BRR | Yield_Mg_ha_BLUE | NEH1_NEH4_2015 | 5.358 | 12.058 | 8.192 | 0.444 | 1.472 | 0.654 |
| E+G+BRR | Yield_Mg_ha_BLUE | NEH2_2015 | 1.777 | 2.555 | 2.497 | 0.696 | 1.023 | 0.712 |
| E+G+BRR | Yield_Mg_ha_BLUE | NEH3_2015 | 3.797 | 3.749 | 10.447 | 1.013 | 0.359 | 0.364 |
| E+G+BRR | Yield_Mg_ha_BLUE | NYH2_2015 | 4.071 | 3.903 | 3.531 | 1.043 | 1.105 | 1.153 |
| E+G+BRR | Yield_Mg_ha_BLUE | NYH3_2015 | 3.704 | 3.589 | 3.979 | 1.032 | 0.902 | 0.931 |
| E+G+BRR | Yield_Mg_ha_BLUE | OHH1_2015 | 2.448 | 2.498 | 3.781 | 0.980 | 0.661 | 0.648 |
| E+G+BRR | Yield_Mg_ha_BLUE | SDH1_2015 | 12.148 | 10.116 | 10.877 | 1.201 | 0.930 | 1.117 |
| E+G+BRR | Yield_Mg_ha_BLUE | Across | - | - | - | 1.150 | 0.719 | 0.753 |
| E+G+BRR | Yield_Mg_ha_weight | DEH1_2015 | 0.198 | 0.040 | 0.056 | 4.889 | 0.727 | 3.552 |
| E+G+BRR | Yield_Mg_ha_weight | GAH1_2015 | 0.328 | 0.584 | 0.483 | 0.561 | 1.210 | 0.679 |
| E+G+BRR | Yield_Mg_ha_weight | INH1_2015 | 0.221 | 1.657 | 0.657 | 0.134 | 2.522 | 0.337 |
| E+G+BRR | Yield_Mg_ha_weight | MNH1_2015 | 0.251 | 0.746 | 0.801 | 0.337 | 0.932 | 0.314 |
| E+G+BRR | Yield_Mg_ha_weight | NCH1_2015 | 0.149 | 0.089 | 0.064 | 1.678 | 1.382 | 2.318 |
| E+G+BRR | Yield_Mg_ha_weight | NEH1_NEH4_2015 | 0.238 | 0.417 | 0.587 | 0.570 | 0.711 | 0.405 |
| E+G+BRR | Yield_Mg_ha_weight | NEH2_2015 | 0.076 | 0.063 | 0.058 | 1.210 | 1.094 | 1.323 |
| E+G+BRR | Yield_Mg_ha_weight | NEH3_2015 | 0.141 | 0.686 | 0.463 | 0.206 | 1.481 | 0.305 |
| E+G+BRR | Yield_Mg_ha_weight | NYH2_2015 | 0.139 | 0.292 | 0.104 | 0.476 | 2.795 | 1.329 |
| E+G+BRR | Yield_Mg_ha_weight | NYH3_2015 | 0.482 | 0.216 | 0.329 | 2.236 | 0.655 | 1.465 |
| E+G+BRR | Yield_Mg_ha_weight | OHH1_2015 | 2.903 | 2.815 | 3.090 | 1.031 | 0.911 | 0.940 |
| E+G+BRR | Yield_Mg_ha_weight | SDH1_2015 | 0.090 | 2.080 | 0.146 | 0.043 | 14.209 | 0.615 |
| E+G+BRR | Yield_Mg_ha_weight | Across | - | - | - | 1.114 | 2.386 | 1.132 |
| E+G+GE+BRR | Grain_Moisture_BLUE | DEH1_2015 | 8.293 | 14.467 | 8.515 | 0.573 | 1.699 | 0.974 |
| E+G+GE+BRR | Grain_Moisture_BLUE | GAH1_2015 | 17.403 | 70.961 | 8.938 | 0.245 | 7.939 | 1.947 |
| E+G+GE+BRR | Grain_Moisture_BLUE | INH1_2015 | 2.106 | 7.254 | 5.391 | 0.290 | 1.346 | 0.391 |
| E+G+GE+BRR | Grain_Moisture_BLUE | MNH1_2015 | 1.919 | 1.848 | 1.189 | 1.038 | 1.555 | 1.615 |
| E+G+GE+BRR | Grain_Moisture_BLUE | NCH1_2015 | 1.200 | 1.380 | 17.104 | 0.870 | 0.081 | 0.070 |
| E+G+GE+BRR | Grain_Moisture_BLUE | NEH1_NEH4_2015 | 4.308 | 12.043 | 1.795 | 0.358 | 6.709 | 2.400 |
| E+G+GE+BRR | Grain_Moisture_BLUE | NEH2_2015 | 9.531 | 40.463 | 19.450 | 0.236 | 2.080 | 0.490 |
| E+G+GE+BRR | Grain_Moisture_BLUE | NEH3_2015 | 4.615 | 7.607 | 5.002 | 0.607 | 1.521 | 0.923 |
| E+G+GE+BRR | Grain_Moisture_BLUE | NYH2_2015 | 3.198 | 1.180 | 1.272 | 2.710 | 0.928 | 2.515 |
| E+G+GE+BRR | Grain_Moisture_BLUE | NYH3_2015 | 9.145 | 26.816 | 6.012 | 0.341 | 4.460 | 1.521 |
| E+G+GE+BRR | Grain_Moisture_BLUE | OHH1_2015 | 4.407 | 3.612 | 7.573 | 1.220 | 0.477 | 0.582 |
| E+G+GE+BRR | Grain_Moisture_BLUE | SDH1_2015 | 14.536 | 29.887 | 13.882 | 0.486 | 2.153 | 1.047 |
| E+G+GE+BRR | Grain_Moisture_BLUE | Across | - | - | - | 0.748 | 2.579 | 1.206 |
| E+G+GE+BRR | Grain_Moisture_weight | DEH1_2015 | 1.208 | 44.017 | 0.485 | 0.028 | 90.795 | 2.492 |
| E+G+GE+BRR | Grain_Moisture_weight | GAH1_2015 | 142.447 | 51.726 | 157.314 | 2.754 | 0.329 | 0.906 |
| E+G+GE+BRR | Grain_Moisture_weight | INH1_2015 | 28.465 | 8.825 | 1.469 | 3.226 | 6.008 | 19.379 |
| E+G+GE+BRR | Grain_Moisture_weight | MNH1_2015 | 11.750 | 1.737 | 3.879 | 6.765 | 0.448 | 3.029 |
| E+G+GE+BRR | Grain_Moisture_weight | NCH1_2015 | 207.235 | 23.765 | 73.513 | 8.720 | 0.323 | 2.819 |
| E+G+GE+BRR | Grain_Moisture_weight | NEH1_NEH4_2015 | 12.290 | 33.473 | 3.306 | 0.367 | 10.125 | 3.718 |
| E+G+GE+BRR | Grain_Moisture_weight | NEH2_2015 | 0.262 | 0.324 | 0.140 | 0.807 | 2.314 | 1.867 |
| E+G+GE+BRR | Grain_Moisture_weight | NEH3_2015 | 88.155 | 3.023 | 1.711 | 29.160 | 1.767 | 51.535 |
| E+G+GE+BRR | Grain_Moisture_weight | NYH2_2015 | 7.591 | 17.633 | 1.960 | 0.431 | 8.995 | 3.872 |
| E+G+GE+BRR | Grain_Moisture_weight | NYH3_2015 | 2.786 | 16.806 | 2.200 | 0.166 | 7.640 | 1.267 |
| E+G+GE+BRR | Grain_Moisture_weight | OHH1_2015 | 42.678 | 0.131 | 0.206 | 326.037 | 0.635 | 207.176 |
| E+G+GE+BRR | Grain_Moisture_weight | SDH1_2015 | 61.817 | 0.240 | 1.292 | 257.787 | 0.186 | 47.865 |
| E+G+GE+BRR | Grain_Moisture_weight | Across | - | - | - | 53.021 | 10.797 | 28.827 |
| E+G+GE+BRR | Yield_Mg_ha_BLUE | DEH1_2015 | 12.318 | 7.167 | 12.223 | 1.719 | 0.586 | 1.008 |
| E+G+GE+BRR | Yield_Mg_ha_BLUE | GAH1_2015 | 2.833 | 5.104 | 6.317 | 0.555 | 0.808 | 0.448 |
| E+G+GE+BRR | Yield_Mg_ha_BLUE | INH1_2015 | 2.710 | 2.770 | 15.087 | 0.978 | 0.184 | 0.180 |
| E+G+GE+BRR | Yield_Mg_ha_BLUE | MNH1_2015 | 7.126 | 2.183 | 6.983 | 3.265 | 0.313 | 1.021 |
| E+G+GE+BRR | Yield_Mg_ha_BLUE | NCH1_2015 | 4.574 | 3.190 | 4.476 | 1.434 | 0.713 | 1.022 |
| E+G+GE+BRR | Yield_Mg_ha_BLUE | NEH1_NEH4_2015 | 5.144 | 12.192 | 7.467 | 0.422 | 1.633 | 0.689 |
| E+G+GE+BRR | Yield_Mg_ha_BLUE | NEH2_2015 | 1.739 | 3.345 | 2.868 | 0.520 | 1.166 | 0.606 |
| E+G+GE+BRR | Yield_Mg_ha_BLUE | NEH3_2015 | 1.760 | 3.128 | 12.449 | 0.563 | 0.251 | 0.141 |
| E+G+GE+BRR | Yield_Mg_ha_BLUE | NYH2_2015 | 5.339 | 4.157 | 3.304 | 1.284 | 1.258 | 1.616 |
| E+G+GE+BRR | Yield_Mg_ha_BLUE | NYH3_2015 | 3.612 | 3.481 | 3.657 | 1.038 | 0.952 | 0.988 |
| E+G+GE+BRR | Yield_Mg_ha_BLUE | OHH1_2015 | 2.447 | 2.761 | 4.582 | 0.886 | 0.603 | 0.534 |
| E+G+GE+BRR | Yield_Mg_ha_BLUE | SDH1_2015 | 11.541 | 10.436 | 11.363 | 1.106 | 0.918 | 1.016 |
| E+G+GE+BRR | Yield_Mg_ha_BLUE | Across | - | - | - | 1.147 | 0.782 | 0.772 |
| E+G+GE+BRR | Yield_Mg_ha_weight | DEH1_2015 | 0.062 | 0.040 | 0.043 | 1.525 | 0.948 | 1.446 |
| E+G+GE+BRR | Yield_Mg_ha_weight | GAH1_2015 | 0.430 | 0.568 | 0.644 | 0.757 | 0.883 | 0.668 |
| E+G+GE+BRR | Yield_Mg_ha_weight | INH1_2015 | 0.225 | 1.297 | 0.175 | 0.174 | 7.395 | 1.285 |
| E+G+GE+BRR | Yield_Mg_ha_weight | MNH1_2015 | 0.168 | 0.587 | 0.469 | 0.287 | 1.250 | 0.359 |
| E+G+GE+BRR | Yield_Mg_ha_weight | NCH1_2015 | 0.271 | 0.167 | 0.065 | 1.628 | 2.566 | 4.176 |
| E+G+GE+BRR | Yield_Mg_ha_weight | NEH1_NEH4_2015 | 0.347 | 0.173 | 0.578 | 2.001 | 0.300 | 0.601 |
| E+G+GE+BRR | Yield_Mg_ha_weight | NEH2_2015 | 0.300 | 0.076 | 0.083 | 3.967 | 0.907 | 3.596 |
| E+G+GE+BRR | Yield_Mg_ha_weight | NEH3_2015 | 0.109 | 0.693 | 0.299 | 0.158 | 2.317 | 0.365 |
| E+G+GE+BRR | Yield_Mg_ha_weight | NYH2_2015 | 0.366 | 0.559 | 0.234 | 0.654 | 2.388 | 1.563 |
| E+G+GE+BRR | Yield_Mg_ha_weight | NYH3_2015 | 0.276 | 0.211 | 0.291 | 1.307 | 0.727 | 0.950 |
| E+G+GE+BRR | Yield_Mg_ha_weight | OHH1_2015 | 2.740 | 2.367 | 2.964 | 1.158 | 0.799 | 0.924 |
| E+G+GE+BRR | Yield_Mg_ha_weight | SDH1_2015 | 0.095 | 1.575 | 0.105 | 0.060 | 14.954 | 0.902 |
| E+G+GE+BRR | Yield_Mg_ha_weight | Across | - | - | - | 1.140 | 2.953 | 1.403 |
|  |  |  |  |  |  |  |  |  |
