# Supplementary material for: Feature engineering of environmental covariates improves plant genomic-enabled prediction
Source: Front Plant Sci. 2024 May 15;15:1349569. doi: 10.3389/fpls.2024.1349569 (PMC11135473; doi:10.3389/fpls.2024.1349569)
Supplement: Supplementary file 2 [file DataSheet_2.docx]

**APPENDIX A**

**BAYESIAN RIDGE REGRESSION**

Bayesian Ridge Regression (BRR) is a probabilistic approach to linear regression that incorporates Bayesian principles. It is a regularized regression method that extends traditional linear regression by introducing a prior distribution over the regression coefficients. This approach provides a way to express uncertainty in the model parameters and helps prevent overfitting by introducing regularization.

The model assumptions assumes a traditional linear regression, with a linear relationship between the independent variables and the dependent variable. The BRR assumes that the coefficients of the regression model follow a Gaussian (normal) distribution. This introduces a regularization term that penalizes large coefficients, helping to prevent overfitting.

The model formulation assumes that ***X*** is an independent variables with and a dependent variable ***y***, such that the BRR can be written as

***y***=***Xβ***+***ϵ***

where *y* is the dependent variable. ***X*** is the matrix of independent variables, ***β*** is the vector of regression coefficients and *ϵ* is the residual (error) term. From a Bayesian perspective, the prior distribution for β is assumed to be Gaussian (normal) **β**∼N(**0**,α^−1^**I**) with α being a hyperparameter controlling the strength of the regularization and **I** is the identity matrix. The goal is to estimate the posterior distribution of **β** given the data. The posterior distribution is proportional to the product of the likelihood and the prior P(**β**∣***X*,*y***)∝*P*(***y***∣***X*,*β***)⋅*P*(***β***). Once the posterior distribution is obtained, Bayesian inference can be performed with. point estimates (mean or mode) of the posterior distribution can be used as the regression coefficients. additionally, credible intervals can be computed to quantify uncertainty.

**APPENDIX B**

**Japonica dataset**

### *Predictor: E+G*

**Table B1** shows an adequate performance for the results under NoEC for the **GC trait** across all environments. The MSE values for 2009, 2010, 2011, 2012, and 2013 were 0.0035, 0.0110, 0.0019, 0.0281, and 0.0017, respectively. Comparing the NoEC results to the EC and FE techniques using Relative Efficiency (RE), all RE values were below 1. On average, NoEC presented 50.050% better performance compared to EC and 42.230% better performance compared to FE. However, when comparing EC and FE techniques based on RE, FE outperformed EC in 2010, 2011, 2012, and 2013, with RE values of 1.287, 2.686, 1.139, and 1.586, respectively. In 2009, EC had a lower RE value of 0.522. On average, the use of FE outperformed EC by 44.410%. Please refer to **Table B1** for more detailed information.

Concerning the GY trait, **Table B1** shows that the use of EC led to a superior performance in most environments based on MSE (796,963 [2009], 2,488,872 [2010] and 1,157,280 [2012]). However, the exceptions occurred in 2011 and 2013, when FE achieved the best MSE values of 2,615,758 and 377,719, respectively. By contrast, when comparing NoEC versus EC and NoEC versus FE using RE, most RE values were greater than 1. On average, the EC technique displayed an improvement of 105.610% (NoEC_vs_EC) regarding the NoEC method, and an improvement of 77.570% (NoEC_vs_FE) was observed with the use of FE compared to the conventional NoEC technique. Nonetheless, when assessing the performance of EC and FE techniques based on RE, FE only outperformed EC in 2011 (RE = 1.091) and 2013 (RE = 1.087). EC, on the other hand, outperformed FE in 2009 (RE = 0.777), 2010 (RE = 0.817), and 2012 (RE = 0.806), resulting in an average RE of 0.916. This indicates an overall performance loss of 8.450% when using FE compared to EC. **Table B1** provides further details.

In terms MSE for the PH trait, **Table B1** shows that the use of FE achieved the best performance in most environments (15.872 [2009], 10.959 [2010], and 164.039 [2012]). However, there were exceptions in 2011 and 2013, where the best MSE values were 28.573 (EC) and 18.363 (NoEC), respectively. On the other hand, when comparing NoEC versus EC and NoEC versus FE techniques using RE, most RE values were greater than 1. On average, the use of EC and FE displayed improvements of 61.570% and 70.210%, respectively, compared to the use of NoEC. Furthermore, when comparing the performance of EC and FE techniques based on RE, FE outperformed EC in all environments, resulting in an average RE of 1.0389. This indicates that using FE surpassed EC by 3.88% (**Table B1**).

In terms of MSE for the PHR trait, **Table B1** indicates that the use of FE yielded the best performance in most environments (0.001 [2009], 0.001 [2010], and 0.001[2013]). However, exceptions were found in 2011 and 2012, when the best MSE values were 0.001 (EC) and 0.006 (NoEC), respectively. On the other hand, when comparing EC versus FE and NoEC versus FE techniques using Relative Efficiency (RE), most RE values were at least 1. On average, the use of FE displayed a general improvement of 22.790%, compared to EC and 7.020% compared to the conventional NoEC technique. However, evaluating the performance of EC versus NoEC techniques based on RE showed that NoEC outperformed EC in most environments, resulting in an average RE of 0.938. This indicates a general accuracy loss of 6.200% when using EC compared to the conventional NoEC technique (**Table B1**).

### *Predictor: E+G+GE*

**Table B1** shows that, in most environments, the conventional NoEC technique yielded the best performance for the GC trait, with MSE values of 0.001 (2009), 0.013 (2010), and 0.002 (2011). The exceptions occurred in 2012 and 2013, with the best MSE values of 0.025 (EC) and 0.0023 (FE). The average RE for the comparison of NoEC versus EC and NoEC versus FE techniques across environments was 0.919 and 0.9023, respectively, indicating general losses of 8.080% and 9.740% for EC and FE compared to the conventional NoEC.

Regarding the GY trait, MSE values from **Table B1** reveal that the use of EC achieved the best performance in most environments (1152261.030 [2009], 3653811.510 [2010], and 989127.170 [2012]). However, exceptions were observed in 2011 and 2013, where the best MSE values were 1834248.25 (NoEC) and 30980.32 (FE), respectively. On the other hand, when comparing NoEC versus EC and NoEC versus FE techniques using RE, most RE values were greater than 1. The average RE for NoEC versus EC and NoEC versus FE was 2.219 and 2.075, respectively, indicating general improvements of 121.860% and 107.520% compared to the use of NoEC. However, an evaluation of the performance of EC and FE techniques based on RE showed that FE outperformed EC only in 2011 (1.0267) and 2013 (1.122), while EC outperformed FE in 2009 (0.789), 2010 (0.849), and 2012 (0.7278). Consequently, the average RE for EC versus FE was 0.9029, implying a general loss of 9.710% when using FE compared to EC (**Table B1**)

Concerning the PH trait, the analysis of MSE values from **Table B1** reveals that the use of FE yielded the best performance in most environments (17.631 [2009] and 23.544 [2012]). However, exceptions were observed in 2010, 2011, and 2013, where the best MSE values were 12.954 (EC), 44.689 (NoEC), and 164.891 (NoEC), respectively. On the other hand, comparing NoEC versus EC and NoEC versus FE techniques using RE showed that most RE values were greater than 1. The average RE for NoEC versus EC and NoEC versus FE was 1.618 and 1.700, respectively, indicating general improvements of 61.810% and 70.000% compared to the conventional NoEC technique. Furthermore, when evaluating the performance of EC and FE techniques based on RE, FE consistently outperformed EC in most environments. The average RE for EC versus FE was 1.047, indicating a 4.710% advantage in favor of FE (**Table B1)**.

Moreover, in the case of the PHR trait, the analysis of MSE values from **Table B1** shows that the use of FE yielded the best performance in most environments (0.001 [2009], 0.002 [2010], and 0.001 [2013]). However, there were exceptions in 2011 and 2012, where the best MSE values were 0.001 (EC) and 0.005 (NoEC), respectively. Furthermore, when comparing the RE values between NoEC versus EC and NoEC versus FE techniques, the average RE values of 0.966 and 1.168 indicate a slight loss of 3.440% and an improvement of 16.800%, respectively, for the use of EC and FE compared to the conventional NoEC technique. Nevertheless, when evaluating the performance of FE versus EC techniques based on RE, FE consistently outperformed EC in most environments. The average RE for FE versus EC was 1.282, indicating a significant improvement of 28.240% in accuracy for using FE compared to (**Table B1**).

### *Predictor: E+G+BRR*

According to **Table B2**, the GC trait displayed superior performances with the conventional NoEC technique in most environments, yielding MSE values of 0.004 (2009), 0.002 (2011), and 0.0012 (2013). However, exceptions were found in 2010 and 2012, where FE achieved the best MSE values of 0.0680 and 0.009, respectively. Comparing the RE values between NoEC versus EC and NoEC versus FE techniques showed that most RE values were below 1. Nonetheless, the average RE of 1.104 (NoEC_vs_EC) and 1.189 (NoEC_vs_FE) indicated that EC and FE outperformed the conventional NoEC technique by 10.360% and 18.930%, respectively. Furthermore, when evaluating the performance of EC and FE techniques based on RE, FE presented the best performance in 2009 (1.151), 2010 (1.353), 2011 (2.044), and 2012 (1.0623), while EC outperformed FE in 2013 (0.529). Overall, the average RE 1.228 indicated that FE outperformed EC by 22.800% (**Table B2**).

Regarding the GY trait, **Table B2** indicates that the conventional NoEC technique displayed superior performances in most environments, with MSE values of 5,683,515.750 (2010), 2,749,626.080 (2012), and 405,886.860 (2013). However, exceptions were observed in 2009 and 2011, where FE achieved the best MSE values of 3,049,246.320 and 4,024,422.450, respectively. When comparing the RE values between NoEC_vs_EC and NoEC_vs_FE techniques, most values were below 1. Nevertheless, the average RE of 1.124 (NoEC_vs_EC) and 0.896 (NoEC_vs_FE) indicated an overall improvement of 12.430% for EC and a general loss of 10.450% for FE compared to the conventional NoEC technique. However, when comparing the performance of EC and FE techniques based on RE, only FE presented a superior performance in 2010 (1.029), resulting in an average RE of 0.797, which indicates a general loss of 20.350% for FE compared to EC (**Table B2**).

For the PH trait, **Table B2** shows that FE yielded the best performance in environments 2009 (15.281) and 2012 (159.312), while EC led to superior performances in environments 2010 (22.962) and 2013 (10.981). Most notably, when comparing the RE values for NoEC_vs_EC and NoEC_vs_FE, values exceeding 1 were observed. The average RE values of 1.634 (NoEC_vs_EC) and 1.5434 (NoEC_vs_FE) indicated substantial improvements of 63.350% and 54.350% respectively for using EC and FE, compared to the conventional NoEC technique. However, in evaluating the performance of EC and FE based on RE, FE exhibited a superior performance in most environments, but still resulting in an average RE of 0.954. This suggests that EC marginally outperformed FE by 4.650%. For further details, see **Table B2.**

Additionally, for the PHR trait, using FE displayed a superior performance in most environments, as indicated in **Table B2.** The best MSE values were observed in 2009 (0.001), 2010 (0.001), and 2013 (0.001). However, exceptions were noted in 2011 and 2012, where the use of EC and NoEC resulted in the best MSE values of 8e-04 and 0.0055, respectively. Furthermore, most RE values comparing NoEC_vs_EC and NoEC_vs_FE techniques were greater than 1. The average RE values of 1.535 (NoEC_vs_EC) and 1.449 (NoEC_vs_FE) indicate significant improvements of 53.530% and 44.930% respectively, compared to the conventional NoEC technique. However, when comparing the performance of the EC versus the FE techniques, the RE values were lower than 1 in most environments, resulting in an average RE of 0.9212. This suggests a general accuracy loss of 7.820% in for using FE compared to using the EC technique (**Table B2).**

### *Predictor: E+G+GE+BRR*

According to **Table B2,** using FE displayed a superior performance for the GC trait in most environments. The best MSE values were observed in 2010 (0.008), 2012 (0.019), and 2013 (0.002). However, exceptions were noted in 2009 and 2011, where the conventional NoEC technique resulted in the best MSE values of 0.001 and 0.0012, respectively. Furthermore, most RE values comparing NoEC_vs_EC and NoEC_vs_FE techniques were greater than 1. The average RE values of 0.7556 (NoEC_vs_EC) and 1.371 (NoEC_vs_FE) indicate that the use of NoEC outperformed EC by 24.430%, while FE outperformed the conventional NoEC technique by 37.050%. Moreover, when comparing the performance of EC and FE techniques based on RE, the FE technique outperformed the EC technique in all environments. The average RE of 1.697 indicates a significant improvement of 69.690% for the use of FE compared to the use of using FE compared to using EC (**Table B2**).

Regarding the GY trait, the analysis in **Table B2** reveals that the use of EC yielded superior results in most environments (2009 [1333530.864], 2012 [1690390.524], and 2013 [584945.854]). However, exceptions were observed in 2010 and 2011, where the NoEC approach resulted in the best MSE values of 4339466.437 and 1834248.259, respectively. Moreover, most RE values for the comparison of NoEC_vs_EC and NoEC_vs_FE techniques were greater than 1. The average RE values of 1.570 (NoEC_vs_EC) and 1.198 (NoEC_vs_FE) indicate general improvements of 57.030% and 19.790% for the use of EC and FE, respectively, compared to the use of NoEC. However, when comparing the performance of EC and FE techniques based on RE, the FE technique did not outperform EC only in 2010, resulting in an average RE of 0.773. This suggests a general loss of 22.670% accuracy for using FE compared to EC.

Regarding the PH trait, **Table B2** shows that the use of FE achieved the best performance in environments 2009 (17.332) and 2011 (22.026), while the use of EC achieved the best performance in environments 2010 (14.9561) and 2013 (11.071). Similarly, most of the RE values for the comparison of NoEC_vs_EC and NoEC_vs_FE techniques were greater than 1. The average RE values of 2.5259 (NoEC_vs_EC) and 2.362 (NoEC_vs_FE) indicate general improvements of 152.590% and 136.210% for using EC and FE, respectively, compared to the conventional NoEC technique. However, when comparing the performance of EC and FE techniques based on RE, EC outperformed FE in most environments, resulting in an average RE of 0.909. This indicates that using EC achieved a 9.100% improvement compared to using FE. For more detailed information, refer to Table 2.

**Table B2** displays that using EC yielded the best performance for the PHR trait in most environments, as indicated by the MSE. Specifically, the MSE values were as follows: 2009 (0.001), 2010 (0.001), 2011 (0.001), and 2013 (0.001). However, in 2012, the best MSE values were 0.005, achieved using both EC and NoEC. Comparing NoEC_vs_EC and NoEC_vs_FE techniques, most RE values were at least 1, with average improvements of 60.350% and 48.570% when using EC and FE, respectively, compared to NoEC. Conversely, when comparing EC versus FE techniques, most environments resulted in an average RE of 0.877, indicating a 12.260% decrease in accuracy when using FE compared to EC (**Table B2**).

| **Table B1**. The prediction performance and the relative efficiency (RE) for **Japonica dataset** in terms of mean squared error (MSE) for each Environment and for each trait, for the predictors E+G and E+G+GE under three different techniques to compute the Kernel for the effect of the Environment: without Environmental Covariates (NoEC), using Environmental covariates (EC) and using Environmental Covariates with Feature Engineering (FE). | | | | | | | | |
| --- | --- | --- | --- | --- | --- | --- | --- | --- |
| Predictor | Trait | Env | NoEC | EC | FE | NoEC_vs_EC | EC_vs_FE | NoEC_vs_FE |
| E+G | GC | 2009 | 0.004 | 0.005 | 0.009 | 0.729 | 0.522 | 0.380 |
| E+G | GC | 2010 | 0.011 | 0.017 | 0.013 | 0.663 | 1.287 | 0.853 |
| E+G | GC | 2011 | 0.002 | 0.009 | 0.004 | 0.202 | 2.686 | 0.543 |
| E+G | GC | 2012 | 0.028 | 0.039 | 0.034 | 0.719 | 1.140 | 0.819 |
| E+G | GC | 2013 | 0.002 | 0.009 | 0.006 | 0.185 | 1.586 | 0.293 |
| E+G | GC | Across | - | - | - | 0.500 | 1.444 | 0.578 |
| E+G | GY | 2009 | 3049246.325 | 796963.009 | 1025847.337 | 3.826 | 0.777 | 2.972 |
| E+G | GY | 2010 | 5683515.755 | 2488872.780 | 3046722.045 | 2.284 | 0.817 | 1.866 |
| E+G | GY | 2011 | 4024422.454 | 2853854.731 | 2615758.363 | 1.410 | 1.091 | 1.539 |
| E+G | GY | 2012 | 2050745.031 | 1157280.313 | 1436429.272 | 1.772 | 0.806 | 1.428 |
| E+G | GY | 2013 | 405886.860 | 410565.496 | 377719.356 | 0.989 | 1.087 | 1.075 |
| E+G | GY | Across | - | - | - | 2.056 | 0.916 | 1.776 |
| E+G | PH | 2009 | 58.674 | 16.561 | 15.872 | 3.543 | 1.043 | 3.697 |
| E+G | PH | 2010 | 27.005 | 12.127 | 10.959 | 2.227 | 1.107 | 2.464 |
| E+G | PH | 2011 | 13.534 | 28.641 | 28.573 | 0.473 | 1.002 | 0.474 |
| E+G | PH | 2012 | 175.254 | 168.840 | 164.039 | 1.038 | 1.029 | 1.068 |
| E+G | PH | 2013 | 18.363 | 23.009 | 22.729 | 0.798 | 1.012 | 0.808 |
| E+G | PH | Across | - | - | - | 1.616 | 1.039 | 1.702 |
| E+G | PHR | 2009 | 0.001 | 0.001 | 0.001 | 0.750 | 1.333 | 1.000 |
| E+G | PHR | 2010 | 0.001 | 0.002 | 0.001 | 0.750 | 1.600 | 1.200 |
| E+G | PHR | 2011 | 0.002 | 0.001 | 0.002 | 1.643 | 0.778 | 1.278 |
| E+G | PHR | 2012 | 0.006 | 0.007 | 0.006 | 0.797 | 1.095 | 0.873 |
| E+G | PHR | 2013 | 0.001 | 0.001 | 0.001 | 0.750 | 1.333 | 1.000 |
| E+G | PHR | Across | - | - | - | 0.938 | 1.228 | 1.070 |
| E+G+GE | GC | 2009 | 0.001 | 0.001 | 0.003 | 0.769 | 0.433 | 0.333 |
| E+G+GE | GC | 2010 | 0.013 | 0.034 | 0.032 | 0.394 | 1.053 | 0.414 |
| E+G+GE | GC | 2011 | 0.002 | 0.006 | 0.003 | 0.281 | 2.462 | 0.692 |
| E+G+GE | GC | 2012 | 0.025 | 0.025 | 0.029 | 1.004 | 0.839 | 0.843 |
| E+G+GE | GC | 2013 | 0.006 | 0.003 | 0.003 | 2.148 | 1.039 | 2.231 |
| E+G+GE | GC | Across | - | - | - | 0.919 | 1.165 | 0.903 |
| E+G+GE | GY | 2009 | 3242702.030 | 1152261.036 | 1460144.165 | 2.814 | 0.789 | 2.221 |
| E+G+GE | GY | 2010 | 4339466.437 | 3653811.519 | 4302236.223 | 1.188 | 0.849 | 1.009 |
| E+G+GE | GY | 2011 | 1834248.259 | 3337540.514 | 3251492.136 | 0.550 | 1.027 | 0.564 |
| E+G+GE | GY | 2012 | 1894112.619 | 989127.176 | 1358843.398 | 1.915 | 0.728 | 1.394 |
| E+G+GE | GY | 2013 | 1924915.862 | 416054.225 | 370980.321 | 4.627 | 1.122 | 5.189 |
| E+G+GE | GY | Across | - | - | - | 2.219 | 0.903 | 2.075 |
| E+G+GE | PH | 2009 | 56.517 | 20.261 | 17.631 | 2.789 | 1.149 | 3.206 |
| E+G+GE | PH | 2010 | 17.957 | 12.954 | 16.142 | 1.386 | 0.803 | 1.112 |
| E+G+GE | PH | 2011 | 44.689 | 77.310 | 64.564 | 0.578 | 1.197 | 0.692 |
| E+G+GE | PH | 2012 | 164.891 | 175.005 | 168.680 | 0.942 | 1.038 | 0.978 |
| E+G+GE | PH | 2013 | 59.136 | 24.696 | 23.544 | 2.395 | 1.049 | 2.512 |
| E+G+GE | PH | Across | - | - | - | 1.618 | 1.047 | 1.700 |
| E+G+GE | PHR | 2009 | 0.001 | 0.001 | 0.001 | 0.750 | 1.333 | 1.000 |
| E+G+GE | PHR | 2010 | 0.002 | 0.002 | 0.002 | 0.818 | 1.467 | 1.200 |
| E+G+GE | PHR | 2011 | 0.002 | 0.001 | 0.001 | 1.727 | 0.917 | 1.583 |
| E+G+GE | PHR | 2012 | 0.005 | 0.007 | 0.006 | 0.783 | 1.095 | 0.857 |
| E+G+GE | PHR | 2013 | 0.001 | 0.001 | 0.001 | 0.750 | 1.600 | 1.200 |
| E+G+GE | PHR | Across | - | - | - | 0.966 | 1.282 | 1.168 |

| **Table B2**. The prediction performance and the relative efficiency (RE) for **Japonica dataset** in terms of mean squared error (MSE) for each Environment and for each trait, for the predictors E+G+BRR and E+G+GE+BRR under three different techniques to compute the Kernel for the effect of the Environment: without Environmental Covariates (NoEC), using Environmental covariates (EC) and using Environmental Covariates with Feature Engineering (FE). | | | | | | | | |
| --- | --- | --- | --- | --- | --- | --- | --- | --- |
| Predictor | Trait | Env | NoEC | EC | FE | NoEC_vs_EC | EC_vs_FE | NoEC_vs_FE |
| E+G+BRR | GC | 2009 | 0.004 | 0.008 | 0.007 | 0.417 | 1.151 | 0.480 |
| E+G+BRR | GC | 2010 | 0.011 | 0.009 | 0.007 | 1.196 | 1.353 | 1.618 |
| E+G+BRR | GC | 2011 | 0.002 | 0.009 | 0.005 | 0.207 | 2.044 | 0.422 |
| E+G+BRR | GC | 2012 | 0.028 | 0.010 | 0.010 | 2.755 | 1.063 | 2.927 |
| E+G+BRR | GC | 2013 | 0.002 | 0.002 | 0.003 | 0.944 | 0.529 | 0.500 |
| E+G+BRR | GC | Across | - | - | - | 1.104 | 1.228 | 1.189 |
| E+G+BRR | GY | 2009 | 3049246.325 | 1221342.669 | 1607864.482 | 2.497 | 0.760 | 1.897 |
| E+G+BRR | GY | 2010 | 5683515.755 | 7662804.296 | 7449307.222 | 0.742 | 1.029 | 0.763 |
| E+G+BRR | GY | 2011 | 4024422.454 | 3689043.326 | 3841776.983 | 1.091 | 0.960 | 1.048 |
| E+G+BRR | GY | 2012 | 2050745.031 | 2749626.084 | 5697594.878 | 0.746 | 0.483 | 0.360 |
| E+G+BRR | GY | 2013 | 405886.860 | 743092.012 | 988735.462 | 0.546 | 0.752 | 0.411 |
| E+G+BRR | GY | Across | - | - | - | 1.124 | 0.797 | 0.896 |
| E+G+BRR | PH | 2009 | 58.674 | 15.466 | 15.281 | 3.794 | 1.012 | 3.840 |
| E+G+BRR | PH | 2010 | 27.005 | 22.962 | 27.436 | 1.176 | 0.837 | 0.984 |
| E+G+BRR | PH | 2011 | 13.534 | 29.033 | 25.921 | 0.466 | 1.120 | 0.522 |
| E+G+BRR | PH | 2012 | 175.254 | 165.479 | 159.312 | 1.059 | 1.039 | 1.100 |
| E+G+BRR | PH | 2013 | 18.363 | 10.981 | 14.450 | 1.672 | 0.760 | 1.271 |
| E+G+BRR | PH | Across | - | - | - | 1.634 | 0.954 | 1.543 |
| E+G+BRR | PHR | 2009 | 0.001 | 0.001 | 0.001 | 1.000 | 1.000 | 1.000 |
| E+G+BRR | PHR | 2010 | 0.001 | 0.001 | 0.001 | 1.714 | 1.167 | 2.000 |
| E+G+BRR | PHR | 2011 | 0.002 | 0.001 | 0.001 | 2.875 | 0.889 | 2.556 |
| E+G+BRR | PHR | 2012 | 0.006 | 0.006 | 0.011 | 0.887 | 0.554 | 0.491 |
| E+G+BRR | PHR | 2013 | 0.001 | 0.001 | 0.001 | 1.200 | 1.000 | 1.200 |
| E+G+BRR | PHR | Across | - | - | - | 1.535 | 0.922 | 1.449 |
| E+G+GE+BRR | GC | 2009 | 0.001 | 0.007 | 0.006 | 0.154 | 1.083 | 0.167 |
| E+G+GE+BRR | GC | 2010 | 0.013 | 0.017 | 0.008 | 0.796 | 2.012 | 1.602 |
| E+G+GE+BRR | GC | 2011 | 0.002 | 0.007 | 0.003 | 0.273 | 2.000 | 0.546 |
| E+G+GE+BRR | GC | 2012 | 0.025 | 0.024 | 0.019 | 1.029 | 1.278 | 1.316 |
| E+G+GE+BRR | GC | 2013 | 0.006 | 0.004 | 0.002 | 1.526 | 2.111 | 3.222 |
| E+G+GE+BRR | GC | Across | - | - | - | 0.756 | 1.697 | 1.371 |
| E+G+GE+BRR | GY | 2009 | 3242702.030 | 1333530.864 | 1860560.276 | 2.432 | 0.717 | 1.743 |
| E+G+GE+BRR | GY | 2010 | 4339466.437 | 7649947.049 | 7468881.672 | 0.567 | 1.024 | 0.581 |
| E+G+GE+BRR | GY | 2011 | 1834248.259 | 4157537.398 | 4872981.083 | 0.441 | 0.853 | 0.376 |
| E+G+GE+BRR | GY | 2012 | 1894112.619 | 1690390.524 | 4082192.704 | 1.121 | 0.414 | 0.464 |
| E+G+GE+BRR | GY | 2013 | 1924915.862 | 584945.854 | 681359.148 | 3.291 | 0.859 | 2.825 |
| E+G+GE+BRR | GY | Across | - | - | - | 1.570 | 0.773 | 1.198 |
| E+G+GE+BRR | PH | 2009 | 56.517 | 18.089 | 17.332 | 3.124 | 1.044 | 3.261 |
| E+G+GE+BRR | PH | 2010 | 17.957 | 14.956 | 26.970 | 1.201 | 0.555 | 0.666 |
| E+G+GE+BRR | PH | 2011 | 44.689 | 22.351 | 22.026 | 1.999 | 1.015 | 2.029 |
| E+G+GE+BRR | PH | 2012 | 164.891 | 171.095 | 167.745 | 0.964 | 1.020 | 0.983 |
| E+G+GE+BRR | PH | 2013 | 59.136 | 11.071 | 12.138 | 5.342 | 0.912 | 4.872 |
| E+G+GE+BRR | PH | Across | - | - | - | 2.526 | 0.909 | 2.362 |
| E+G+GE+BRR | PHR | 2009 | 0.001 | 0.001 | 0.001 | 1.000 | 1.000 | 1.000 |
| E+G+GE+BRR | PHR | 2010 | 0.002 | 0.001 | 0.001 | 2.571 | 1.000 | 2.571 |
| E+G+GE+BRR | PHR | 2011 | 0.002 | 0.001 | 0.001 | 2.375 | 1.000 | 2.375 |
| E+G+GE+BRR | PHR | 2012 | 0.005 | 0.006 | 0.011 | 0.871 | 0.554 | 0.482 |
| E+G+GE+BRR | PHR | 2013 | 0.001 | 0.001 | 0.001 | 1.200 | 0.833 | 1.000 |
| E+G+GE+BRR | PHR | Across | - | - | - | 1.604 | 0.877 | 1.486 |

### USP dataset

### *Predictor: E+G*

Upon examining **Table B3**, it becomes apparent that the conventional NoEC technique achieved the best performance in terms of MSE in environments Env2 (4.073) and Env3 (5.246). However, exceptions were found in Env1 and Env4, where the optimal MSE values were 3.141 (FE) and 7.814 (EC), respectively. For further detail, refer to **Table B3.**

**Table B3** present our comparison results between the NoEC and EC techniques, assessed through the RE metric. The EC technique displayed its best performance in environments Env1 (1.059) and Env4 (1.046), showcasing improvements of 5.920% and 4.610% over the NoEC technique, respectively. However, NoEC outperformed EC in environments Env2 (0.869) and Env3 (0.831), resulting in an average RE of 0.951. This average RE indicates a general loss of 4.890% in accuracy when using EC compared to NoEC (see **Table B3**).

The EC and FE techniques were compared, using the RE metric to assess their performance. The findings indicate that the FE technique achieved its best performance in environments Env1 (1.045) and Env2 (1.048), displaying improvements of 4.480% and 4.790% over EC. However, EC exhibited a slightly better performance in environments Env3 (0.979) and Env4 (0.946), resulting in an average RE of 1.004. This average RE suggests a modest improvement of 0.430% when using FE compared to EC (see **Table B3**).

### *Predictor: E+G+GE*

**Table B3** reveals the performance of the FE technique in terms of MSE across different environments. The FE technique achieved its best performance in environments Env1 (2.789) and Env2 (4.636), although exceptions were found in Env3 and Env4, where the optimal MSE values were 5.833 (NoEC) and 7.792 (EC), respectively (see **Table 3**)

**Table B3** present our comparison results between the NoEC and EC techniques, based on the RE metric. The EC technique displayed its best performance in environments Env1 (1.107) and Env4 (1.120), showing improvements of 10.72% and 12.040% over the NoEC technique. However, the NoEC technique outperformed EC in environments Env2 (0.961) and Env3 (0.925), resulting in an average RE of 1.028. This average RE indicates a general improvement of 2.840% of the EC method regarding the NoEC technique (see **Table B3**).

The EC and FE techniques were compared, using the RE metric to assess their performance. The findings indicate that the FE technique achieved its best performance in environments Env1 (1.054) and Env2 (1.057), displaying improvements of 5.380% and 5.650% over EC. However, using EC exhibited a better performance in environments Env3 (0.986) and Env4 (0.949), resulting in an average RE of 1.012. This average RE indicates a 1.150% improvement of the FE technique over EC (see **Table B3**).

### *Predictor: E+G+BRR*

**Table B4** presents the results of our analysis regarding the MSE about the FE technique. The FE technique performed best in Env1 (2.859) and Env3 (4.413) environments. However, exceptions were observed in Env2 and Env4, where the optimal MSE values were 4.073 (NoEC) and 5.638 (EC), respectively. For further details, see **Table B4**.

The results of our comparison between the NoEC and EC techniques, based on the RE metric, are presented in **Table B4.** The EC technique exhibited its best performance in environments Env1 (1.171) and Env4 (1.450), suggesting improvements of 17.1000% and 45.000%, respectively, compared to the NoEC technique. However, the NoEC technique outperformed EC in environments Env2 (0.823) and Env3 (0.836), resulting in an average RE of 1.070. This average RE indicates a general improvement of 7.000% of the EC regarding the NoEC technique (see **Table B4**)

We compared the EC and FE techniques, evaluating their performance with the RE metric. The findings indicate that the FE technique achieved its best performance in environments Env1 (1.038) and Env3 (1.423), displaying respective improvements of 3.840% and 42.290% over EC. However, EC performed better in environments Env2 (0.934) and Env4 (0.990), resulting in an average RE of 1.096. This average RE indicates a 9.600% better performance of the FE technique over EC (see **Table B4**).

### *Predictor: E+G+GE+BRR*

**Table B4** presents the performance results of the FE technique in terms of MSE. The best performance was observed in environments Env1 (2.644), Env3 (4.265), and Env4 (5.856). The only exception was Env2, where the optimal MSE value was 4.708, achieved using NoEC. For further information, see **Table B4.**

Based on the RE metric, the results of our comparison between the NoEC and EC techniques are presented in **Table B4**. EC performed best in environments Env1 (1.175) and Env4 (1.465), with improvements of 17.510% and 46.530%, respectively, compared to the NoEC technique. However, the NoEC technique outperformed EC in environments Env2 (0.958) and Env3 (0.915), resulting in an average RE of 1.128. This average RE indicates a general improvement of 12.830% of EC regarding NoEC. For more specific information, see **Table B4**.

We compared the EC and FE techniques based on the RE metric. The analysis revealed that the FE technique displayed its best performance in Env1 (1.047), Env3 (1.494), and Env4 (1.017). These results indicate improvements of 4.740%, 49.430%, and 1.740%, respectively, when compared to using EC. However, EC displayed a better performance in Env2 (0.941), but in general, the FE technique outperformed EC by 12.500%, since an average RE of 1.125 was observed (see **Table B4**).

| **Table B3.** The prediction performance and the relative efficiency (RE) for **USP dataset** in terms of mean squared error (MSE) for each Environment and for each trait, for the predictors E+G and E+G+GE under three different techniques to compute the Kernel for the effect of the Environment: without Environmental Covariates (NoEC), using Environmental covariates (EC) and using Environmental Covariates with Feature Engineering (FE). | | | | | | | | |
| --- | --- | --- | --- | --- | --- | --- | --- | --- |
| Predictor | Trait | Env | NoEC | EC | FE | NoEC_vs_EC | EC_vs_FE | NoEC_vs_FE |
| E+G | GY | Env1 | 3.476 | 3.281 | 3.141 | 1.059 | 1.045 | 1.107 |
| E+G | GY | Env2 | 4.073 | 4.689 | 4.475 | 0.869 | 1.048 | 0.910 |
| E+G | GY | Env3 | 5.246 | 6.317 | 6.455 | 0.831 | 0.979 | 0.813 |
| E+G | GY | Env4 | 8.174 | 7.814 | 8.262 | 1.046 | 0.946 | 0.989 |
| E+G | GY | Across | - | - | - | 0.951 | 1.004 | 0.955 |
| E+G+GE | GY | Env1 | 3.254 | 2.939 | 2.789 | 1.107 | 1.054 | 1.167 |
| E+G+GE | GY | Env2 | 4.708 | 4.898 | 4.636 | 0.961 | 1.057 | 1.016 |
| E+G+GE | GY | Env3 | 5.833 | 6.307 | 6.396 | 0.925 | 0.986 | 0.912 |
| E+G+GE | GY | Env4 | 8.730 | 7.792 | 8.206 | 1.120 | 0.950 | 1.064 |
| E+G+GE | GY | Across | - | - | - | 1.028 | 1.012 | 1.040 |

| **Table B4**. The prediction performance and the relative efficiency (RE) for **USP dataset** in terms of mean squared error (MSE) for each Environment and for each trait, for the predictors E+G+BRR and E+G+GE+BRR under three different techniques to compute the Kernel for the effect of the Environment: without Environmental Covariates (NoEC), using Environmental covariates (EC) and using Environmental Covariates with Feature Engineering (FE). | | | | | | | | |
| --- | --- | --- | --- | --- | --- | --- | --- | --- |
| Predictor | Trait | Env | NoEC | EC | FE | NoEC_vs_EC | EC_vs_FE | NoEC_vs_FE |
| E+G+BRR | GY | Env1 | 3.476 | 2.968 | 2.859 | 1.171 | 1.038 | 1.216 |
| E+G+BRR | GY | Env2 | 4.073 | 4.951 | 5.301 | 0.823 | 0.934 | 0.768 |
| E+G+BRR | GY | Env3 | 5.246 | 6.279 | 4.413 | 0.836 | 1.423 | 1.189 |
| E+G+BRR | GY | Env4 | 8.174 | 5.638 | 5.696 | 1.450 | 0.990 | 1.435 |
| E+G+BRR | GY | Across | - | - | - | 1.070 | 1.096 | 1.152 |
| E+G+GE+BRR | GY | Env1 | 3.254 | 2.769 | 2.644 | 1.175 | 1.047 | 1.231 |
| E+G+GE+BRR | GY | Env2 | 4.708 | 4.917 | 5.224 | 0.958 | 0.941 | 0.901 |
| E+G+GE+BRR | GY | Env3 | 5.833 | 6.373 | 4.265 | 0.915 | 1.494 | 1.368 |
| E+G+GE+BRR | GY | Env4 | 8.730 | 5.958 | 5.856 | 1.465 | 1.017 | 1.491 |
| E+G+GE+BRR | GY | Across | - | - | - | 1.128 | 1.125 | 1.248 |

### G2F_2016 dataset

### *Predictor: E+G*

**Table B5** illustrates that FE yielded the best performance for the Grain_Moisture_BLUE trait in most environments. MSE values were 4.645 (DEH1_2016), 2.154 (GAH1_2016), 2.703 (IAH1_2016), 0.467 (IAH4_2016), 0.668 (MOH1_2016), 3.598 (NCH1_2016), 2.092 (NYH2_2016), and 1.601 (WIH2_2016). The average RE values showed that FE outperformed EC and NoEC by 87.970% and 119.370%, respectively. Additionally, EC displayed an average RE improvement of 63.960% over NoEC. For further detail, see **Table B5**.

For the Grain_Moisture_weight trait, EC presented the best performance based on MSE values in several environments listed in Table 5 (ARH1_2016 [24.235], DEH1_2016 [0.207], IAH1_2016 [2.568], ILH1_2016 [2.172], INH1_2016 [0.210], MOH1_2016 [7.450], OHH1_2016 [0.454] and WIH2_2016 [0.194]). The average RE values revealed that EC and FE outperformed the conventional NoEC technique by 1682.340% and 245.980%, respectively. Furthermore, FE displayed a 58.100% improvement over EC (See **Table B5**)

Regarding the Yield_Mg_ha_BLUE trait, NoEC displayed a superior performance in most environments based on MSE values listed in **Table B5** (GAH1_2016 [3.579], IAH4_2016 [2.576], MIH1_2016 [4.045], MNH1_2016 [1.268], NYH2_2016 [16.252], OHH1_2016 [1.830] and WIH1_2016 (3.665]). The average RE values indicated that FE resulted in general improvements of 21.030% and 1.400% over EC and NoEC, respectively. However, a comparison between NoEC and EC showed a slight decrease of 0.190% in average RE for EC (see **Table B5**).

For the Yield_Mg_ha_weight trait, NoEC showed the best performance based on MSE values in most environments (DEH1_2016 [0.078], IAH4_2016 [0.091], ILH1_2016 [0.351], MIH1_2016 [0.1156], MNH1_2016 [0.391], NYH2_2016 [0.087], WIH1_2016 [0.063] and WIH2_2016 [0.019]). The average RE values indicated general improvements of 52.860% and 22.630% for EC and FE, respectively, compared to NoEC. Moreover, on average, FE outperformed EC by 89.600% (see **Table B5**).

### *Predictor: E+G+GE*

**Table B5** shows that FE yielded the best performance for the Grain_Moisture_BLUE trait in the majority of environments, with MSE values ranging from 0.519 to 5.813 (IAH4_2016, ILH1_2016, MNH1_2016, NEH1_2016, NYH2_2016, OHH1_2016 and WIH1_2016). Comparing RE values, using FE outperformed EC and NoEC techniques by 42.480% and 114.740%, respectively. Additionally, EC outperformed NoEC with an average RE of 1.552, indicating a superiority of 55.210% for EC. For further details, see **Table B5.**

For the Grain_Moisture_weight trait, **Table B5** reveals that FE displayed a better performance in most environments, as indicated by the MSE values (DEH1_2016 [0.132], IAH3_2016 [0.418], IAH4_2016 [139.446], MIH1_2016 [1.668], MNH1_2016 [1.316], NCH1_2016 [6.953], NYH2_2016 [5.565], OHH1_2016 [0.195] and WIH1_2016 [1.508]). Moreover, the average RE values showed that FE outperformed EC and NoEC by 831.910% and 825.260%, respectively. Comparing NoEC and EC techniques, there was a general improvement of 357.000% for EC over NoEC, with an average RE of 4.570 (see **Table B5**).

Regarding the Yield_Mg_ha_BLUE trait, **Table B5** shows that the use of NoEC achieved the best performance in most environments, as indicated by the MSE values (GAH1_2016 [3.379], IAH1_2016 [2.287], IAH2_2016 [7.505], IAH4_2016 [3.565], MIH1_2016 [4.748], NYH2_2016 [17.271], WIH1_2016 [2.210] and WIH2_2016 [4.667]). However, most RE values comparing NoEC_vs_EC and NoEC_vs_FENoEC_vs_FEtechniques were greater than 1. On average, EC displayed a 7.450% improvement and FE showed an 11.690% improvement compared to the conventional NoEC technique. Furthermore, comparing EC and FE techniques, an average RE of 1.227 was observed, indicating that FE outperformed NoEC by 22.700% (see **Table B5**)

In terms of the Yield_Mg_ha_weight trait, **Table B5** shows that the use of NoEC achieved the best performance in most environments, as evident from the MSE values (DEH1_2016 [0.051], GAH1_2016 [0.026], IAH1_2016 [2.914], IAH2_2016 [0.0689], MIH1_2016 [0.055], MNH1_2016 [0.146], NEH1_2016 [0.033], NYH2_2016 [0.449] and OHH1_2016 [1.202]). The average RE values indicated slight losses of 2.210% and 2.570% when comparing EC versus NoEC and FE versus NoEC, respectively. This implies that EC and FE techniques did not perform as adequately as the conventional NoEC technique. However, comparing EC and FE techniques based on RE showed that FE outperformed EC in most environments, resulting in an average RE of 1.339, indicating a 33.930% superiority of FE over EC. For more detailed information, see **Table B5.**

### *Predictor: E+G+BRR*

In **Table B6,** it is evident that for the Grain_Moisture_BLUE trait, the use of FE provided the best performance in most environments, as indicated by the MSE values (DEH1_2016 [4.376], GAH1_2016 [2.002], IAH1_2016 [2.036], IAH3_2016 [1.237], IAH4_2016 [0.496], MNH1_2016 [3.685], MOH1_2016 [0.678], NCH1_2016 [3.499], NYH2_2016 [2.213] and WIH2_2016 [1.648]). On average, the RE values indicate that FE outperformed EC and NoEC by 67.090% and 129.850%, respectively. Additionally, comparing NoEC and EC techniques showed that EC outperformed NoEC by an average of 84.880%. For further information, see **Table B6.**

For the Grain_Moisture_weight trait, **Table B6** shows that the use of NoEC provided the best performance in most environments, as indicated by the MSE values (GAH1_2016 [1.272], IAH1_2016 [401.574], IAH3_2016 [0.199], ILH1_2016 [5.447], MIH1_2016 [0.715[, NCH1_2016 [1.174] and NEH1_2016 [42.758]). On average, the RE values indicate that FE outperformed EC and NoEC by 167.270% and 25.410%, respectively. Furthermore, comparing NoEC and EC shows that EC outperformed NoEC with an average RE of 3.495, representing a general improvement of 149.510%. For more detailed information, see **Table B6.**

**Table B6,** for the Yield_Mg_ha_BLUE trait, shows that the use of NoEC led to the best performance in most environments, as indicated by the MSE values (ARH1_2016 [3.713], GAH1_2016 [3.579], IAH4_2016 [2.576], INH1_2016 [2016], MIH1_2016 [4.045], MNH1_2016 [1.268], NYH2_2016 [16.252], OHH1_2016 [1.829] and WIH2_2016 [4.629]). On average, the RE values indicate general improvements of 10.650% for FE compared to EC, and 3.780% for EC compared to NoEC. However, when comparing the performance of NoEC and FE techniques, an average RE of 0.986 indicates a slight loss for FE compared to NoEC. For more detailed information, see **Table B6.**

For the Yield_Mg_ha_weight trait, the use of NoEC achieved the best performance in most environments, as indicated by the MSE values (ARH1_2016 [0.989], GAH1_3016 [0.035], IAH2_2016 [0.175], IAH3_2016 [0.783], MIH1_2016 [0.1201], MHH1_2016 [0.393], MOH1_2016 [0.232], NYH2_2016 [0.402], OHH1_2016 [0.533] and WIH2_2016 [0.055]). On average, the RE values indicate a general improvement of 51.630% for EC compared to NoEC and 3.960% for FE compared to EC. However, when comparing the performance of NoEC and FE based on RE, the best performance was displayed by NoEC in most environments, resulting in an average RE of 0.9012, indicating that NoEC outperformed FE by 9.820%. For more detailed information, see **Table B6.**

### *Predictor: E+G+GE+BRR*

**Table B6** shows that EC yielded the most favorable results for the Grain_Moisture_BLUE trait in various environments. The corresponding MSE values for EC were 4.2801 (DEH1_2016), 0.519 (IAH4_2016), 4.964 (ILH1_2016), 4.762 (MNH1_2016), 6.047 (NEH1_2016), 4.030 (NYH2_2016), 3.072 (OHH1_2016), and 3.495 (WIH1_2016). Additionally, the average RE values indicated that using FE outperformed both EC and NoEC by 29.090% and 125.150%, respectively (1.291 for EC_vs_FE, and 2.252 for NoEC_vs_FE). Furthermore, when comparing the NoEC and EC techniques, an average RE of 1.6512 displays the superior performance of EC over NoEC by 65.180%. For more comprehensive information, see **Table B6.**

When considering the Grain_Moisture_weight trait, the use of EC presented amor adequate performance in most environments based on the MSE values provided in Table 6 (DEH1_2016 [0.595], IAH1_2016 [360.363], IAH2_2016 [1.219], IAH4_2016 [120.354], ILH1_2016 [10.357], NCH1_2016 [2.0245], NYH2_2016 [2.534], and OHH1_2016 [4.124]). Moreover, the average RE values reveal that EC and FE outperformed the conventional NoEC by 408.510% and 240.900% respectively (5.085 for NoEC_vs_EC and 3.409 for NoEC_vs_FE). Furthermore, a comparison between EC and FE techniques indicates that an average RE of 7.899 suggests that FE outperformed EC by 689.960%. For more detailed information, see **Table B6.**

When examining the Yield_Mg_ha_BLUE trait, the use of NoEC displayed the best performance in most environments based on the MSE values presented in **Table B6** (ARH1_2016 [3.928], GAH1_2016 [3.379], IAH1_2016 [2.287], IAH2_2016 [7.505], IAH4_2016 [2.565], MIH1_2016 [4.748], NYH2_2016 [17.271], WIH1_2016 [2.210] and WIH2_2016 [4.667]). However, it is worth noting that EC and FE outperformed the conventional NoEC by 11.690% and 9.490% in terms of average RE values (1.117 for NoEC_vs_EC and 1.095 for NoEC_vs_FE). Nevertheless, when comparing FE versus EC techniques, a slight loss of 0.400% was observed for using FE compared to EC, as indicated by an average RE of 0.996. For more detailed information, see **Table B6.**

Regarding the Yield_Mg_ha_weight trait, **Table B6** shows that the use of EC yielded the best performance in most environments, as evidenced by the following MSE values: ARH1_2016 (0.719), DEH1_2016 (0.020), IAH1_2016 (2.808), IAH4_2016 (0.058), ILH1_2016 (0.594), NYH2_2016 (0.418), and WIH2_2016 (0.073). The average RE values indicated improvements of 22.200% (NoEC_vs_EC) and 11.380% (NoEC_vs_FE), highlighting the superior performance of EC and FE over the conventional NoEC technique. Conversely, when comparing EC and FE techniques, most environments performed better with an average RE of 1.384, indicating that FE outperformed EC by 38.420%. For additional information, see **Table B6.**

| **Table B5**. The prediction performance and the relative efficiency (RE) for **G2F_2016** **dataset** in terms of mean squared error (MSE) for each Environment and for each trait, for the predictors E+G and E+G+GE under three different techniques to compute the Kernel for the effect of the Environment: without Environmental Covariates (NoEC), using Environmental covariates (EC) and using Environmental Covariates with Feature Engineering (FE). | | | | | | | | |
| --- | --- | --- | --- | --- | --- | --- | --- | --- |
| Predictor | Trait | Env | NoEC | EC | FE | NoEC_vs_EC | EC_vs_FE | NoEC_vs_FE |
| E+G | Grain_Moisture_BLUE | ARH1_2016 | 1.733 | 6.108 | 1.886 | 0.284 | 3.238 | 0.919 |
| E+G | Grain_Moisture_BLUE | DEH1_2016 | 7.863 | 5.829 | 4.645 | 1.349 | 1.255 | 1.693 |
| E+G | Grain_Moisture_BLUE | GAH1_2016 | 6.686 | 5.107 | 2.154 | 1.309 | 2.371 | 3.105 |
| E+G | Grain_Moisture_BLUE | IAH1_2016 | 9.814 | 7.419 | 2.703 | 1.323 | 2.745 | 3.632 |
| E+G | Grain_Moisture_BLUE | IAH2_2016 | 3.124 | 0.866 | 1.694 | 3.608 | 0.511 | 1.844 |
| E+G | Grain_Moisture_BLUE | IAH3_2016 | 1.456 | 2.981 | 1.486 | 0.489 | 2.006 | 0.980 |
| E+G | Grain_Moisture_BLUE | IAH4_2016 | 2.495 | 0.506 | 0.467 | 4.932 | 1.084 | 5.344 |
| E+G | Grain_Moisture_BLUE | ILH1_2016 | 4.556 | 3.436 | 9.783 | 1.326 | 0.351 | 0.466 |
| E+G | Grain_Moisture_BLUE | INH1_2016 | 1.934 | 9.982 | 2.887 | 0.194 | 3.457 | 0.670 |
| E+G | Grain_Moisture_BLUE | MIH1_2016 | 2.988 | 3.101 | 3.366 | 0.963 | 0.922 | 0.888 |
| E+G | Grain_Moisture_BLUE | MNH1_2016 | 17.117 | 4.471 | 4.483 | 3.829 | 0.997 | 3.818 |
| E+G | Grain_Moisture_BLUE | MOH1_2016 | 0.809 | 3.068 | 0.668 | 0.264 | 4.593 | 1.211 |
| E+G | Grain_Moisture_BLUE | NCH1_2016 | 21.208 | 10.860 | 3.598 | 1.953 | 3.018 | 5.895 |
| E+G | Grain_Moisture_BLUE | NEH1_2016 | 6.193 | 4.897 | 10.060 | 1.265 | 0.487 | 0.616 |
| E+G | Grain_Moisture_BLUE | NYH2_2016 | 7.475 | 3.625 | 2.092 | 2.062 | 1.732 | 3.573 |
| E+G | Grain_Moisture_BLUE | OHH1_2016 | 4.840 | 2.834 | 5.728 | 1.708 | 0.495 | 0.845 |
| E+G | Grain_Moisture_BLUE | WIH1_2016 | 5.143 | 2.599 | 3.788 | 1.979 | 0.686 | 1.358 |
| E+G | Grain_Moisture_BLUE | WIH2_2016 | 4.219 | 6.224 | 1.601 | 0.678 | 3.887 | 2.634 |
| E+G | Grain_Moisture_BLUE | Across | - | - | - | 1.640 | 1.880 | 2.194 |
| E+G | Grain_Moisture_weight | ARH1_2016 | 30.391 | 24.235 | 30.934 | 1.254 | 0.783 | 0.982 |
| E+G | Grain_Moisture_weight | DEH1_2016 | 14.987 | 0.207 | 2.910 | 72.261 | 0.071 | 5.150 |
| E+G | Grain_Moisture_weight | GAH1_2016 | 1.272 | 2.339 | 7.133 | 0.544 | 0.328 | 0.178 |
| E+G | Grain_Moisture_weight | IAH1_2016 | 401.574 | 481.573 | 510.263 | 0.834 | 0.944 | 0.787 |
| E+G | Grain_Moisture_weight | IAH2_2016 | 6.212 | 2.568 | 25.510 | 2.419 | 0.101 | 0.244 |
| E+G | Grain_Moisture_weight | IAH3_2016 | 0.199 | 10.913 | 31.831 | 0.018 | 0.343 | 0.006 |
| E+G | Grain_Moisture_weight | IAH4_2016 | 311.023 | 244.775 | 180.018 | 1.271 | 1.360 | 1.728 |
| E+G | Grain_Moisture_weight | ILH1_2016 | 5.447 | 2.172 | 25.900 | 2.507 | 0.084 | 0.210 |
| E+G | Grain_Moisture_weight | INH1_2016 | 1.274 | 0.210 | 0.325 | 6.058 | 0.647 | 3.916 |
| E+G | Grain_Moisture_weight | MIH1_2016 | 0.715 | 8.311 | 0.872 | 0.086 | 9.531 | 0.820 |
| E+G | Grain_Moisture_weight | MNH1_2016 | 7.866 | 43.427 | 6.379 | 0.181 | 6.808 | 1.233 |
| E+G | Grain_Moisture_weight | MOH1_2016 | 27.122 | 7.450 | 44.113 | 3.640 | 0.169 | 0.615 |
| E+G | Grain_Moisture_weight | NCH1_2016 | 1.174 | 4.278 | 10.042 | 0.274 | 0.426 | 0.117 |
| E+G | Grain_Moisture_weight | NEH1_2016 | 42.758 | 63.944 | 64.869 | 0.669 | 0.986 | 0.659 |
| E+G | Grain_Moisture_weight | NYH2_2016 | 1.893 | 2.551 | 11.545 | 0.742 | 0.221 | 0.164 |
| E+G | Grain_Moisture_weight | OHH1_2016 | 63.776 | 0.454 | 27.250 | 140.383 | 0.017 | 2.340 |
| E+G | Grain_Moisture_weight | WIH1_2016 | 1.373 | 7.073 | 1.371 | 0.194 | 5.160 | 1.001 |
| E+G | Grain_Moisture_weight | WIH2_2016 | 16.972 | 0.194 | 0.403 | 87.485 | 0.482 | 42.125 |
| E+G | Grain_Moisture_weight | Across | - | - | - | 17.823 | 1.581 | 3.460 |
| E+G | Yield_Mg_ha_BLUE | ARH1_2016 | 3.713 | 3.199 | 14.552 | 1.161 | 0.220 | 0.255 |
| E+G | Yield_Mg_ha_BLUE | DEH1_2016 | 5.330 | 3.354 | 4.354 | 1.589 | 0.770 | 1.224 |
| E+G | Yield_Mg_ha_BLUE | GAH1_2016 | 3.580 | 10.264 | 4.606 | 0.349 | 2.229 | 0.777 |
| E+G | Yield_Mg_ha_BLUE | IAH1_2016 | 3.187 | 2.897 | 1.395 | 1.100 | 2.077 | 2.286 |
| E+G | Yield_Mg_ha_BLUE | IAH2_2016 | 7.921 | 7.684 | 8.073 | 1.031 | 0.952 | 0.981 |
| E+G | Yield_Mg_ha_BLUE | IAH3_2016 | 5.918 | 4.741 | 3.772 | 1.248 | 1.257 | 1.569 |
| E+G | Yield_Mg_ha_BLUE | IAH4_2016 | 2.576 | 2.718 | 3.708 | 0.948 | 0.733 | 0.695 |
| E+G | Yield_Mg_ha_BLUE | ILH1_2016 | 8.719 | 4.698 | 6.260 | 1.856 | 0.750 | 1.393 |
| E+G | Yield_Mg_ha_BLUE | INH1_2016 | 2.415 | 3.018 | 2.406 | 0.800 | 1.254 | 1.004 |
| E+G | Yield_Mg_ha_BLUE | MIH1_2016 | 4.045 | 5.627 | 16.686 | 0.719 | 0.337 | 0.242 |
| E+G | Yield_Mg_ha_BLUE | MNH1_2016 | 1.268 | 1.301 | 1.270 | 0.975 | 1.025 | 0.999 |
| E+G | Yield_Mg_ha_BLUE | MOH1_2016 | 7.968 | 4.191 | 10.428 | 1.901 | 0.402 | 0.764 |
| E+G | Yield_Mg_ha_BLUE | NCH1_2016 | 4.467 | 10.571 | 3.293 | 0.423 | 3.211 | 1.357 |
| E+G | Yield_Mg_ha_BLUE | NEH1_2016 | 4.993 | 4.832 | 4.188 | 1.033 | 1.154 | 1.192 |
| E+G | Yield_Mg_ha_BLUE | NYH2_2016 | 16.252 | 22.790 | 16.626 | 0.713 | 1.371 | 0.978 |
| E+G | Yield_Mg_ha_BLUE | OHH1_2016 | 1.830 | 4.790 | 2.558 | 0.382 | 1.872 | 0.715 |
| E+G | Yield_Mg_ha_BLUE | WIH1_2016 | 3.665 | 5.021 | 3.785 | 0.730 | 1.326 | 0.968 |
| E+G | Yield_Mg_ha_BLUE | WIH2_2016 | 4.630 | 4.588 | 5.420 | 1.009 | 0.846 | 0.854 |
| E+G | Yield_Mg_ha_BLUE | Across | - | - | - | 0.998 | 1.210 | 1.014 |
| E+G | Yield_Mg_ha_weight | ARH1_2016 | 0.989 | 1.000 | 1.542 | 0.989 | 0.649 | 0.641 |
| E+G | Yield_Mg_ha_weight | DEH1_2016 | 0.163 | 0.078 | 0.230 | 2.088 | 0.340 | 0.710 |
| E+G | Yield_Mg_ha_weight | GAH1_2016 | 0.035 | 0.439 | 0.288 | 0.079 | 1.522 | 0.120 |
| E+G | Yield_Mg_ha_weight | IAH1_2016 | 3.743 | 3.345 | 3.151 | 1.119 | 1.061 | 1.188 |
| E+G | Yield_Mg_ha_weight | IAH2_2016 | 0.175 | 0.668 | 0.077 | 0.262 | 8.629 | 2.261 |
| E+G | Yield_Mg_ha_weight | IAH3_2016 | 0.788 | 1.704 | 1.583 | 0.462 | 1.076 | 0.498 |
| E+G | Yield_Mg_ha_weight | IAH4_2016 | 0.498 | 0.091 | 0.105 | 5.491 | 0.861 | 4.729 |
| E+G | Yield_Mg_ha_weight | ILH1_2016 | 1.113 | 0.351 | 0.754 | 3.172 | 0.465 | 1.476 |
| E+G | Yield_Mg_ha_weight | INH1_2016 | 0.055 | 0.077 | 0.052 | 0.709 | 1.486 | 1.054 |
| E+G | Yield_Mg_ha_weight | MIH1_2016 | 0.121 | 0.116 | 0.132 | 1.042 | 0.875 | 0.912 |
| E+G | Yield_Mg_ha_weight | MNH1_2016 | 0.393 | 0.391 | 0.711 | 1.005 | 0.551 | 0.553 |
| E+G | Yield_Mg_ha_weight | MOH1_2016 | 0.232 | 1.501 | 0.172 | 0.155 | 8.721 | 1.348 |
| E+G | Yield_Mg_ha_weight | NCH1_2016 | 0.083 | 0.343 | 0.085 | 0.241 | 4.062 | 0.978 |
| E+G | Yield_Mg_ha_weight | NEH1_2016 | 0.036 | 0.038 | 0.029 | 0.963 | 1.279 | 1.231 |
| E+G | Yield_Mg_ha_weight | NYH2_2016 | 0.402 | 0.087 | 0.139 | 4.601 | 0.630 | 2.899 |
| E+G | Yield_Mg_ha_weight | OHH1_2016 | 0.533 | 1.326 | 0.876 | 0.402 | 1.514 | 0.608 |
| E+G | Yield_Mg_ha_weight | WIH1_2016 | 0.117 | 0.063 | 0.209 | 1.877 | 0.299 | 0.561 |
| E+G | Yield_Mg_ha_weight | WIH2_2016 | 0.055 | 0.019 | 0.180 | 2.860 | 0.107 | 0.306 |
| E+G | Yield_Mg_ha_weight | Across | - | - | - | 1.529 | 1.896 | 1.226 |
| E+G+GE | Grain_Moisture_BLUE | ARH1_2016 | 2.003 | 6.545 | 4.641 | 0.306 | 1.410 | 0.432 |
| E+G+GE | Grain_Moisture_BLUE | DEH1_2016 | 5.256 | 5.689 | 10.400 | 0.924 | 0.547 | 0.505 |
| E+G+GE | Grain_Moisture_BLUE | GAH1_2016 | 5.841 | 3.993 | 2.715 | 1.463 | 1.471 | 2.152 |
| E+G+GE | Grain_Moisture_BLUE | IAH1_2016 | 2.857 | 5.585 | 3.541 | 0.512 | 1.577 | 0.807 |
| E+G+GE | Grain_Moisture_BLUE | IAH2_2016 | 0.713 | 1.504 | 1.785 | 0.475 | 0.843 | 0.400 |
| E+G+GE | Grain_Moisture_BLUE | IAH3_2016 | 2.933 | 4.648 | 2.860 | 0.631 | 1.625 | 1.025 |
| E+G+GE | Grain_Moisture_BLUE | IAH4_2016 | 1.622 | 0.519 | 0.695 | 3.123 | 0.747 | 2.333 |
| E+G+GE | Grain_Moisture_BLUE | ILH1_2016 | 8.071 | 4.093 | 9.622 | 1.972 | 0.425 | 0.839 |
| E+G+GE | Grain_Moisture_BLUE | INH1_2016 | 5.315 | 10.531 | 4.891 | 0.505 | 2.153 | 1.087 |
| E+G+GE | Grain_Moisture_BLUE | MIH1_2016 | 2.448 | 3.313 | 5.501 | 0.739 | 0.602 | 0.445 |
| E+G+GE | Grain_Moisture_BLUE | MNH1_2016 | 13.571 | 5.813 | 6.414 | 2.335 | 0.906 | 2.116 |
| E+G+GE | Grain_Moisture_BLUE | MOH1_2016 | 3.450 | 5.296 | 1.357 | 0.651 | 3.904 | 2.543 |
| E+G+GE | Grain_Moisture_BLUE | NCH1_2016 | 14.869 | 8.231 | 2.333 | 1.806 | 3.528 | 6.374 |
| E+G+GE | Grain_Moisture_BLUE | NEH1_2016 | 12.527 | 5.166 | 10.466 | 2.425 | 0.494 | 1.197 |
| E+G+GE | Grain_Moisture_BLUE | NYH2_2016 | 9.727 | 4.423 | 5.172 | 2.199 | 0.855 | 1.881 |
| E+G+GE | Grain_Moisture_BLUE | OHH1_2016 | 6.975 | 2.849 | 6.176 | 2.448 | 0.461 | 1.129 |
| E+G+GE | Grain_Moisture_BLUE | WIH1_2016 | 6.024 | 3.056 | 5.975 | 1.971 | 0.512 | 1.008 |
| E+G+GE | Grain_Moisture_BLUE | WIH2_2016 | 21.532 | 6.235 | 1.739 | 3.454 | 3.585 | 12.382 |
| E+G+GE | Grain_Moisture_BLUE | Across | - | - | - | 1.552 | 1.425 | 2.147 |
| E+G+GE | Grain_Moisture_weight | ARH1_2016 | 14.116 | 3.206 | 9.123 | 4.403 | 0.351 | 1.547 |
| E+G+GE | Grain_Moisture_weight | DEH1_2016 | 1.608 | 10.772 | 0.132 | 0.149 | 81.919 | 12.231 |
| E+G+GE | Grain_Moisture_weight | GAH1_2016 | 0.862 | 0.883 | 4.521 | 0.976 | 0.195 | 0.191 |
| E+G+GE | Grain_Moisture_weight | IAH1_2016 | 501.269 | 514.108 | 546.300 | 0.975 | 0.941 | 0.918 |
| E+G+GE | Grain_Moisture_weight | IAH2_2016 | 43.354 | 23.631 | 36.310 | 1.835 | 0.651 | 1.194 |
| E+G+GE | Grain_Moisture_weight | IAH3_2016 | 11.456 | 7.015 | 0.418 | 1.633 | 16.769 | 27.387 |
| E+G+GE | Grain_Moisture_weight | IAH4_2016 | 265.697 | 167.322 | 139.446 | 1.588 | 1.200 | 1.905 |
| E+G+GE | Grain_Moisture_weight | ILH1_2016 | 35.818 | 2.973 | 32.902 | 12.047 | 0.090 | 1.089 |
| E+G+GE | Grain_Moisture_weight | INH1_2016 | 51.327 | 1.919 | 3.812 | 26.741 | 0.504 | 13.465 |
| E+G+GE | Grain_Moisture_weight | MIH1_2016 | 18.430 | 38.977 | 1.668 | 0.473 | 23.368 | 11.049 |
| E+G+GE | Grain_Moisture_weight | MNH1_2016 | 11.304 | 39.937 | 1.316 | 0.283 | 30.345 | 8.589 |
| E+G+GE | Grain_Moisture_weight | MOH1_2016 | 3.665 | 14.395 | 291.204 | 0.255 | 0.049 | 0.013 |
| E+G+GE | Grain_Moisture_weight | NCH1_2016 | 7.758 | 7.873 | 6.953 | 0.985 | 1.132 | 1.116 |
| E+G+GE | Grain_Moisture_weight | NEH1_2016 | 113.669 | 88.519 | 99.451 | 1.284 | 0.890 | 1.143 |
| E+G+GE | Grain_Moisture_weight | NYH2_2016 | 80.595 | 16.174 | 5.565 | 4.983 | 2.906 | 14.482 |
| E+G+GE | Grain_Moisture_weight | OHH1_2016 | 12.108 | 0.596 | 0.195 | 20.319 | 3.054 | 62.060 |
| E+G+GE | Grain_Moisture_weight | WIH1_2016 | 11.902 | 4.475 | 1.508 | 2.660 | 2.967 | 7.892 |
| E+G+GE | Grain_Moisture_weight | WIH2_2016 | 0.917 | 1.365 | 3.320 | 0.672 | 0.411 | 0.276 |
| E+G+GE | Grain_Moisture_weight | Across | - | - | - | 4.570 | 9.319 | 9.253 |
| E+G+GE | Yield_Mg_ha_BLUE | ARH1_2016 | 3.928 | 2.896 | 14.301 | 1.357 | 0.203 | 0.275 |
| E+G+GE | Yield_Mg_ha_BLUE | DEH1_2016 | 5.964 | 3.522 | 3.831 | 1.694 | 0.919 | 1.557 |
| E+G+GE | Yield_Mg_ha_BLUE | GAH1_2016 | 3.379 | 10.667 | 4.157 | 0.317 | 2.566 | 0.813 |
| E+G+GE | Yield_Mg_ha_BLUE | IAH1_2016 | 2.287 | 2.778 | 2.820 | 0.823 | 0.985 | 0.811 |
| E+G+GE | Yield_Mg_ha_BLUE | IAH2_2016 | 7.505 | 8.311 | 7.733 | 0.903 | 1.075 | 0.971 |
| E+G+GE | Yield_Mg_ha_BLUE | IAH3_2016 | 7.908 | 6.619 | 5.280 | 1.195 | 1.254 | 1.498 |
| E+G+GE | Yield_Mg_ha_BLUE | IAH4_2016 | 2.565 | 2.895 | 3.811 | 0.886 | 0.760 | 0.673 |
| E+G+GE | Yield_Mg_ha_BLUE | ILH1_2016 | 8.036 | 4.761 | 5.919 | 1.688 | 0.804 | 1.358 |
| E+G+GE | Yield_Mg_ha_BLUE | INH1_2016 | 6.533 | 2.424 | 1.994 | 2.696 | 1.216 | 3.277 |
| E+G+GE | Yield_Mg_ha_BLUE | MIH1_2016 | 4.748 | 7.252 | 19.667 | 0.655 | 0.369 | 0.241 |
| E+G+GE | Yield_Mg_ha_BLUE | MNH1_2016 | 1.422 | 1.479 | 1.265 | 0.961 | 1.169 | 1.124 |
| E+G+GE | Yield_Mg_ha_BLUE | MOH1_2016 | 12.381 | 5.928 | 9.392 | 2.089 | 0.631 | 1.318 |
| E+G+GE | Yield_Mg_ha_BLUE | NCH1_2016 | 5.713 | 11.008 | 3.515 | 0.519 | 3.132 | 1.626 |
| E+G+GE | Yield_Mg_ha_BLUE | NEH1_2016 | 5.446 | 5.707 | 5.214 | 0.954 | 1.095 | 1.045 |
| E+G+GE | Yield_Mg_ha_BLUE | NYH2_2016 | 17.271 | 24.594 | 19.504 | 0.702 | 1.261 | 0.886 |
| E+G+GE | Yield_Mg_ha_BLUE | OHH1_2016 | 2.503 | 4.763 | 2.138 | 0.526 | 2.227 | 1.171 |
| E+G+GE | Yield_Mg_ha_BLUE | WIH1_2016 | 2.210 | 5.855 | 3.805 | 0.378 | 1.539 | 0.581 |
| E+G+GE | Yield_Mg_ha_BLUE | WIH2_2016 | 4.667 | 4.667 | 5.288 | 1.000 | 0.883 | 0.883 |
| E+G+GE | Yield_Mg_ha_BLUE | Across | - | - | - | 1.075 | 1.227 | 1.117 |
| E+G+GE | Yield_Mg_ha_weight | ARH1_2016 | 2.359 | 1.339 | 1.540 | 1.762 | 0.869 | 1.532 |
| E+G+GE | Yield_Mg_ha_weight | DEH1_2016 | 0.051 | 0.124 | 0.284 | 0.410 | 0.437 | 0.179 |
| E+G+GE | Yield_Mg_ha_weight | GAH1_2016 | 0.026 | 0.357 | 0.258 | 0.074 | 1.385 | 0.102 |
| E+G+GE | Yield_Mg_ha_weight | IAH1_2016 | 2.914 | 3.540 | 3.508 | 0.823 | 1.009 | 0.831 |
| E+G+GE | Yield_Mg_ha_weight | IAH2_2016 | 0.069 | 0.410 | 0.076 | 0.168 | 5.378 | 0.903 |
| E+G+GE | Yield_Mg_ha_weight | IAH3_2016 | 0.670 | 0.608 | 1.186 | 1.102 | 0.513 | 0.565 |
| E+G+GE | Yield_Mg_ha_weight | IAH4_2016 | 0.199 | 0.110 | 0.082 | 1.807 | 1.343 | 2.426 |
| E+G+GE | Yield_Mg_ha_weight | ILH1_2016 | 0.751 | 0.468 | 0.539 | 1.605 | 0.868 | 1.394 |
| E+G+GE | Yield_Mg_ha_weight | INH1_2016 | 0.112 | 0.056 | 0.046 | 1.981 | 1.227 | 2.429 |
| E+G+GE | Yield_Mg_ha_weight | MIH1_2016 | 0.055 | 0.189 | 0.172 | 0.291 | 1.098 | 0.320 |
| E+G+GE | Yield_Mg_ha_weight | MNH1_2016 | 0.146 | 0.352 | 0.502 | 0.415 | 0.701 | 0.291 |
| E+G+GE | Yield_Mg_ha_weight | MOH1_2016 | 0.283 | 0.295 | 0.263 | 0.959 | 1.122 | 1.076 |
| E+G+GE | Yield_Mg_ha_weight | NCH1_2016 | 0.113 | 0.388 | 0.104 | 0.292 | 3.730 | 1.090 |
| E+G+GE | Yield_Mg_ha_weight | NEH1_2016 | 0.033 | 0.073 | 0.081 | 0.458 | 0.900 | 0.412 |
| E+G+GE | Yield_Mg_ha_weight | NYH2_2016 | 0.449 | 0.781 | 0.709 | 0.575 | 1.102 | 0.633 |
| E+G+GE | Yield_Mg_ha_weight | OHH1_2016 | 1.202 | 1.667 | 1.328 | 0.721 | 1.255 | 0.905 |
| E+G+GE | Yield_Mg_ha_weight | WIH1_2016 | 0.204 | 0.096 | 0.132 | 2.125 | 0.729 | 1.550 |
| E+G+GE | Yield_Mg_ha_weight | WIH2_2016 | 0.185 | 0.091 | 0.204 | 2.036 | 0.443 | 0.903 |
| E+G+GE | Yield_Mg_ha_weight | Across | - | - | - | 0.978 | 1.339 | 0.974 |

| **Table B6**. The prediction performance and the relative efficiency (RE) for **G2F_2016 dataset** in terms of mean squared error (MSE) for each Environment and for each trait, for the predictor E+G+BRR and E+G+GE+BRR under three different techniques to compute the Kernel for the effect of the Environment: without Environmental Covariates (NoEC), using Environmental covariates (EC) and using Environmental Covariates with Feature Engineering (FE). | | | | | | | | |
| --- | --- | --- | --- | --- | --- | --- | --- | --- |
| Predictor | Trait | Env | NoEC | EC | FE | NoEC_vs_EC | EC_vs_FE | NoEC_vs_FE |
| E+G+BRR | Grain_Moisture_BLUE | ARH1_2016 | 1.733 | 8.086 | 2.856 | 0.214 | 2.832 | 0.607 |
| E+G+BRR | Grain_Moisture_BLUE | DEH1_2016 | 7.863 | 5.151 | 4.376 | 1.526 | 1.177 | 1.797 |
| E+G+BRR | Grain_Moisture_BLUE | GAH1_2016 | 6.686 | 5.025 | 2.002 | 1.331 | 2.511 | 3.341 |
| E+G+BRR | Grain_Moisture_BLUE | IAH1_2016 | 9.814 | 3.372 | 2.036 | 2.911 | 1.656 | 4.821 |
| E+G+BRR | Grain_Moisture_BLUE | IAH2_2016 | 3.124 | 1.172 | 1.650 | 2.665 | 0.711 | 1.894 |
| E+G+BRR | Grain_Moisture_BLUE | IAH3_2016 | 1.456 | 2.060 | 1.237 | 0.707 | 1.665 | 1.178 |
| E+G+BRR | Grain_Moisture_BLUE | IAH4_2016 | 2.495 | 0.515 | 0.496 | 4.845 | 1.039 | 5.031 |
| E+G+BRR | Grain_Moisture_BLUE | ILH1_2016 | 4.556 | 2.970 | 9.745 | 1.534 | 0.305 | 0.468 |
| E+G+BRR | Grain_Moisture_BLUE | INH1_2016 | 1.934 | 12.122 | 2.526 | 0.160 | 4.798 | 0.766 |
| E+G+BRR | Grain_Moisture_BLUE | MIH1_2016 | 2.988 | 3.562 | 3.335 | 0.839 | 1.068 | 0.896 |
| E+G+BRR | Grain_Moisture_BLUE | MNH1_2016 | 17.117 | 3.852 | 3.685 | 4.444 | 1.045 | 4.645 |
| E+G+BRR | Grain_Moisture_BLUE | MOH1_2016 | 0.809 | 1.362 | 0.678 | 0.594 | 2.009 | 1.193 |
| E+G+BRR | Grain_Moisture_BLUE | NCH1_2016 | 21.208 | 10.060 | 3.499 | 2.108 | 2.875 | 6.061 |
| E+G+BRR | Grain_Moisture_BLUE | NEH1_2016 | 6.193 | 2.846 | 9.795 | 2.176 | 0.291 | 0.632 |
| E+G+BRR | Grain_Moisture_BLUE | NYH2_2016 | 7.475 | 2.344 | 2.213 | 3.189 | 1.059 | 3.378 |
| E+G+BRR | Grain_Moisture_BLUE | OHH1_2016 | 4.840 | 2.898 | 5.870 | 1.670 | 0.494 | 0.825 |
| E+G+BRR | Grain_Moisture_BLUE | WIH1_2016 | 5.143 | 3.045 | 4.014 | 1.689 | 0.759 | 1.281 |
| E+G+BRR | Grain_Moisture_BLUE | WIH2_2016 | 4.219 | 6.235 | 1.648 | 0.677 | 3.785 | 2.560 |
| E+G+BRR | Grain_Moisture_BLUE | Across | - | - | - | 1.849 | 1.671 | 2.299 |
| E+G+BRR | Grain_Moisture_weight | ARH1_2016 | 30.391 | 7.962 | 7.088 | 3.817 | 1.123 | 4.288 |
| E+G+BRR | Grain_Moisture_weight | DEH1_2016 | 14.987 | 0.443 | 5.442 | 33.869 | 0.081 | 2.754 |
| E+G+BRR | Grain_Moisture_weight | GAH1_2016 | 1.272 | 5.393 | 2.233 | 0.236 | 2.415 | 0.569 |
| E+G+BRR | Grain_Moisture_weight | IAH1_2016 | 401.574 | 459.125 | 508.319 | 0.875 | 0.903 | 0.790 |
| E+G+BRR | Grain_Moisture_weight | IAH2_2016 | 6.212 | 1.611 | 176.584 | 3.855 | 0.009 | 0.035 |
| E+G+BRR | Grain_Moisture_weight | IAH3_2016 | 0.199 | 51.438 | 110.303 | 0.004 | 0.466 | 0.002 |
| E+G+BRR | Grain_Moisture_weight | IAH4_2016 | 311.023 | 188.044 | 160.261 | 1.654 | 1.173 | 1.941 |
| E+G+BRR | Grain_Moisture_weight | ILH1_2016 | 5.447 | 22.946 | 64.425 | 0.237 | 0.356 | 0.085 |
| E+G+BRR | Grain_Moisture_weight | INH1_2016 | 1.274 | 0.691 | 0.685 | 1.843 | 1.009 | 1.860 |
| E+G+BRR | Grain_Moisture_weight | MIH1_2016 | 0.715 | 31.083 | 1.554 | 0.023 | 20.002 | 0.460 |
| E+G+BRR | Grain_Moisture_weight | MNH1_2016 | 7.866 | 43.882 | 6.124 | 0.179 | 7.165 | 1.284 |
| E+G+BRR | Grain_Moisture_weight | MOH1_2016 | 27.122 | 21.394 | 393.212 | 1.268 | 0.054 | 0.069 |
| E+G+BRR | Grain_Moisture_weight | NCH1_2016 | 1.174 | 5.985 | 24.041 | 0.196 | 0.249 | 0.049 |
| E+G+BRR | Grain_Moisture_weight | NEH1_2016 | 42.758 | 57.295 | 90.340 | 0.746 | 0.634 | 0.473 |
| E+G+BRR | Grain_Moisture_weight | NYH2_2016 | 1.893 | 0.666 | 46.015 | 2.842 | 0.015 | 0.041 |
| E+G+BRR | Grain_Moisture_weight | OHH1_2016 | 63.776 | 7.228 | 19.206 | 8.823 | 0.376 | 3.321 |
| E+G+BRR | Grain_Moisture_weight | WIH1_2016 | 1.373 | 13.412 | 1.266 | 0.102 | 10.595 | 1.084 |
| E+G+BRR | Grain_Moisture_weight | WIH2_2016 | 16.972 | 7.246 | 4.891 | 2.342 | 1.482 | 3.470 |
| E+G+BRR | Grain_Moisture_weight | Across | - | - | - | 3.495 | 2.673 | 1.254 |
| E+G+BRR | Yield_Mg_ha_BLUE | ARH1_2016 | 3.713 | 3.799 | 14.569 | 0.977 | 0.261 | 0.255 |
| E+G+BRR | Yield_Mg_ha_BLUE | DEH1_2016 | 5.330 | 2.922 | 3.946 | 1.824 | 0.740 | 1.351 |
| E+G+BRR | Yield_Mg_ha_BLUE | GAH1_2016 | 3.580 | 11.055 | 5.613 | 0.324 | 1.970 | 0.638 |
| E+G+BRR | Yield_Mg_ha_BLUE | IAH1_2016 | 3.187 | 1.743 | 1.393 | 1.829 | 1.252 | 2.289 |
| E+G+BRR | Yield_Mg_ha_BLUE | IAH2_2016 | 7.921 | 7.568 | 8.528 | 1.047 | 0.888 | 0.929 |
| E+G+BRR | Yield_Mg_ha_BLUE | IAH3_2016 | 5.918 | 5.873 | 6.247 | 1.008 | 0.940 | 0.947 |
| E+G+BRR | Yield_Mg_ha_BLUE | IAH4_2016 | 2.576 | 2.618 | 3.773 | 0.984 | 0.694 | 0.683 |
| E+G+BRR | Yield_Mg_ha_BLUE | ILH1_2016 | 8.719 | 4.687 | 7.329 | 1.860 | 0.640 | 1.190 |
| E+G+BRR | Yield_Mg_ha_BLUE | INH1_2016 | 2.415 | 2.675 | 2.435 | 0.903 | 1.098 | 0.992 |
| E+G+BRR | Yield_Mg_ha_BLUE | MIH1_2016 | 4.045 | 6.342 | 17.412 | 0.638 | 0.364 | 0.232 |
| E+G+BRR | Yield_Mg_ha_BLUE | MNH1_2016 | 1.268 | 1.350 | 1.270 | 0.939 | 1.063 | 0.999 |
| E+G+BRR | Yield_Mg_ha_BLUE | MOH1_2016 | 7.968 | 4.093 | 10.724 | 1.947 | 0.382 | 0.743 |
| E+G+BRR | Yield_Mg_ha_BLUE | NCH1_2016 | 4.467 | 9.870 | 3.889 | 0.453 | 2.538 | 1.149 |
| E+G+BRR | Yield_Mg_ha_BLUE | NEH1_2016 | 4.993 | 4.703 | 3.515 | 1.062 | 1.338 | 1.421 |
| E+G+BRR | Yield_Mg_ha_BLUE | NYH2_2016 | 16.252 | 22.892 | 17.091 | 0.710 | 1.339 | 0.951 |
| E+G+BRR | Yield_Mg_ha_BLUE | OHH1_2016 | 1.830 | 4.374 | 2.456 | 0.418 | 1.781 | 0.745 |
| E+G+BRR | Yield_Mg_ha_BLUE | WIH1_2016 | 3.665 | 4.548 | 2.558 | 0.806 | 1.778 | 1.433 |
| E+G+BRR | Yield_Mg_ha_BLUE | WIH2_2016 | 4.630 | 4.859 | 5.700 | 0.953 | 0.853 | 0.812 |
| E+G+BRR | Yield_Mg_ha_BLUE | Across | - | - | - | 1.038 | 1.107 | 0.986 |
| E+G+BRR | Yield_Mg_ha_weight | ARH1_2016 | 0.989 | 1.219 | 1.311 | 0.811 | 0.930 | 0.755 |
| E+G+BRR | Yield_Mg_ha_weight | DEH1_2016 | 0.163 | 0.029 | 0.076 | 5.723 | 0.375 | 2.143 |
| E+G+BRR | Yield_Mg_ha_weight | GAH1_2016 | 0.035 | 0.251 | 0.134 | 0.138 | 1.870 | 0.259 |
| E+G+BRR | Yield_Mg_ha_weight | IAH1_2016 | 3.743 | 3.506 | 3.050 | 1.068 | 1.150 | 1.227 |
| E+G+BRR | Yield_Mg_ha_weight | IAH2_2016 | 0.175 | 0.372 | 3.081 | 0.471 | 0.121 | 0.057 |
| E+G+BRR | Yield_Mg_ha_weight | IAH3_2016 | 0.788 | 0.976 | 2.401 | 0.808 | 0.407 | 0.328 |
| E+G+BRR | Yield_Mg_ha_weight | IAH4_2016 | 0.498 | 0.065 | 0.179 | 7.678 | 0.362 | 2.782 |
| E+G+BRR | Yield_Mg_ha_weight | ILH1_2016 | 1.113 | 0.336 | 0.581 | 3.316 | 0.578 | 1.916 |
| E+G+BRR | Yield_Mg_ha_weight | INH1_2016 | 0.055 | 0.044 | 0.058 | 1.239 | 0.761 | 0.943 |
| E+G+BRR | Yield_Mg_ha_weight | MIH1_2016 | 0.121 | 0.297 | 0.300 | 0.406 | 0.992 | 0.402 |
| E+G+BRR | Yield_Mg_ha_weight | MNH1_2016 | 0.393 | 0.721 | 0.682 | 0.546 | 1.057 | 0.577 |
| E+G+BRR | Yield_Mg_ha_weight | MOH1_2016 | 0.232 | 0.521 | 0.252 | 0.445 | 2.066 | 0.920 |
| E+G+BRR | Yield_Mg_ha_weight | NCH1_2016 | 0.083 | 0.311 | 0.078 | 0.266 | 4.012 | 1.066 |
| E+G+BRR | Yield_Mg_ha_weight | NEH1_2016 | 0.036 | 0.030 | 0.031 | 1.203 | 0.984 | 1.183 |
| E+G+BRR | Yield_Mg_ha_weight | NYH2_2016 | 0.402 | 0.419 | 0.700 | 0.960 | 0.598 | 0.574 |
| E+G+BRR | Yield_Mg_ha_weight | OHH1_2016 | 0.533 | 1.561 | 1.276 | 0.341 | 1.224 | 0.418 |
| E+G+BRR | Yield_Mg_ha_weight | WIH1_2016 | 0.117 | 0.067 | 0.207 | 1.746 | 0.324 | 0.566 |
| E+G+BRR | Yield_Mg_ha_weight | WIH2_2016 | 0.055 | 0.424 | 0.469 | 0.130 | 0.904 | 0.118 |
| E+G+BRR | Yield_Mg_ha_weight | Across | - | - | - | 1.516 | 1.040 | 0.902 |
| E+G+GE+BRR | Grain_Moisture_BLUE | ARH1_2016 | 2.003 | 8.861 | 8.335 | 0.226 | 1.063 | 0.240 |
| E+G+GE+BRR | Grain_Moisture_BLUE | DEH1_2016 | 5.256 | 4.281 | 9.799 | 1.228 | 0.437 | 0.536 |
| E+G+GE+BRR | Grain_Moisture_BLUE | GAH1_2016 | 5.841 | 4.396 | 2.596 | 1.329 | 1.693 | 2.250 |
| E+G+GE+BRR | Grain_Moisture_BLUE | IAH1_2016 | 2.857 | 3.613 | 2.833 | 0.791 | 1.275 | 1.008 |
| E+G+GE+BRR | Grain_Moisture_BLUE | IAH2_2016 | 0.713 | 1.881 | 1.708 | 0.379 | 1.101 | 0.418 |
| E+G+GE+BRR | Grain_Moisture_BLUE | IAH3_2016 | 2.933 | 3.283 | 2.610 | 0.893 | 1.258 | 1.124 |
| E+G+GE+BRR | Grain_Moisture_BLUE | IAH4_2016 | 1.622 | 0.519 | 0.724 | 3.127 | 0.717 | 2.241 |
| E+G+GE+BRR | Grain_Moisture_BLUE | ILH1_2016 | 8.071 | 4.964 | 9.657 | 1.626 | 0.514 | 0.836 |
| E+G+GE+BRR | Grain_Moisture_BLUE | INH1_2016 | 5.315 | 11.384 | 4.258 | 0.467 | 2.674 | 1.248 |
| E+G+GE+BRR | Grain_Moisture_BLUE | MIH1_2016 | 2.448 | 3.737 | 3.645 | 0.655 | 1.025 | 0.672 |
| E+G+GE+BRR | Grain_Moisture_BLUE | MNH1_2016 | 13.571 | 4.762 | 6.621 | 2.850 | 0.719 | 2.050 |
| E+G+GE+BRR | Grain_Moisture_BLUE | MOH1_2016 | 3.450 | 2.107 | 1.221 | 1.637 | 1.725 | 2.825 |
| E+G+GE+BRR | Grain_Moisture_BLUE | NCH1_2016 | 14.869 | 7.756 | 2.226 | 1.917 | 3.485 | 6.681 |
| E+G+GE+BRR | Grain_Moisture_BLUE | NEH1_2016 | 12.527 | 6.047 | 10.506 | 2.072 | 0.576 | 1.192 |
| E+G+GE+BRR | Grain_Moisture_BLUE | NYH2_2016 | 9.727 | 4.030 | 5.378 | 2.414 | 0.749 | 1.809 |
| E+G+GE+BRR | Grain_Moisture_BLUE | OHH1_2016 | 6.975 | 3.072 | 8.466 | 2.270 | 0.363 | 0.824 |
| E+G+GE+BRR | Grain_Moisture_BLUE | WIH1_2016 | 6.024 | 3.495 | 6.151 | 1.723 | 0.568 | 0.979 |
| E+G+GE+BRR | Grain_Moisture_BLUE | WIH2_2016 | 21.532 | 5.216 | 1.584 | 4.128 | 3.293 | 13.594 |
| E+G+GE+BRR | Grain_Moisture_BLUE | Across | - | - | - | 1.652 | 1.291 | 2.252 |
| E+G+GE+BRR | Grain_Moisture_weight | ARH1_2016 | 14.116 | 33.005 | 48.706 | 0.428 | 0.678 | 0.290 |
| E+G+GE+BRR | Grain_Moisture_weight | DEH1_2016 | 1.608 | 0.595 | 0.683 | 2.701 | 0.872 | 2.355 |
| E+G+GE+BRR | Grain_Moisture_weight | GAH1_2016 | 0.862 | 3.261 | 1.258 | 0.264 | 2.593 | 0.685 |
| E+G+GE+BRR | Grain_Moisture_weight | IAH1_2016 | 501.269 | 360.363 | 452.522 | 1.391 | 0.796 | 1.108 |
| E+G+GE+BRR | Grain_Moisture_weight | IAH2_2016 | 43.354 | 1.219 | 28.797 | 35.562 | 0.042 | 1.506 |
| E+G+GE+BRR | Grain_Moisture_weight | IAH3_2016 | 11.456 | 92.472 | 220.035 | 0.124 | 0.420 | 0.052 |
| E+G+GE+BRR | Grain_Moisture_weight | IAH4_2016 | 265.697 | 120.354 | 139.962 | 2.208 | 0.860 | 1.898 |
| E+G+GE+BRR | Grain_Moisture_weight | ILH1_2016 | 35.818 | 10.357 | 65.451 | 3.459 | 0.158 | 0.547 |
| E+G+GE+BRR | Grain_Moisture_weight | INH1_2016 | 51.327 | 29.589 | 16.709 | 1.735 | 1.771 | 3.072 |
| E+G+GE+BRR | Grain_Moisture_weight | MIH1_2016 | 18.430 | 47.158 | 9.360 | 0.391 | 5.039 | 1.969 |
| E+G+GE+BRR | Grain_Moisture_weight | MNH1_2016 | 11.304 | 52.703 | 0.445 | 0.215 | 118.486 | 25.414 |
| E+G+GE+BRR | Grain_Moisture_weight | MOH1_2016 | 3.665 | 5.633 | 128.039 | 0.651 | 0.044 | 0.029 |
| E+G+GE+BRR | Grain_Moisture_weight | NCH1_2016 | 7.758 | 2.025 | 11.167 | 3.831 | 0.181 | 0.695 |
| E+G+GE+BRR | Grain_Moisture_weight | NEH1_2016 | 113.669 | 56.705 | 50.862 | 2.005 | 1.115 | 2.235 |
| E+G+GE+BRR | Grain_Moisture_weight | NYH2_2016 | 80.595 | 2.534 | 6.431 | 31.802 | 0.394 | 12.532 |
| E+G+GE+BRR | Grain_Moisture_weight | OHH1_2016 | 12.108 | 4.124 | 14.744 | 2.936 | 0.280 | 0.821 |
| E+G+GE+BRR | Grain_Moisture_weight | WIH1_2016 | 11.902 | 7.400 | 2.403 | 1.608 | 3.080 | 4.954 |
| E+G+GE+BRR | Grain_Moisture_weight | WIH2_2016 | 0.917 | 4.113 | 0.764 | 0.223 | 5.385 | 1.201 |
| E+G+GE+BRR | Grain_Moisture_weight | Across | - | - | - | 5.085 | 7.900 | 3.409 |
| E+G+GE+BRR | Yield_Mg_ha_BLUE | ARH1_2016 | 3.928 | 14.301 | 15.060 | 0.275 | 0.950 | 0.261 |
| E+G+GE+BRR | Yield_Mg_ha_BLUE | DEH1_2016 | 5.964 | 3.831 | 3.763 | 1.557 | 1.018 | 1.585 |
| E+G+GE+BRR | Yield_Mg_ha_BLUE | GAH1_2016 | 3.379 | 4.157 | 4.699 | 0.813 | 0.885 | 0.719 |
| E+G+GE+BRR | Yield_Mg_ha_BLUE | IAH1_2016 | 2.287 | 2.820 | 2.767 | 0.811 | 1.019 | 0.826 |
| E+G+GE+BRR | Yield_Mg_ha_BLUE | IAH2_2016 | 7.505 | 7.733 | 8.012 | 0.971 | 0.965 | 0.937 |
| E+G+GE+BRR | Yield_Mg_ha_BLUE | IAH3_2016 | 7.908 | 5.280 | 4.834 | 1.498 | 1.092 | 1.636 |
| E+G+GE+BRR | Yield_Mg_ha_BLUE | IAH4_2016 | 2.565 | 3.811 | 3.842 | 0.673 | 0.992 | 0.668 |
| E+G+GE+BRR | Yield_Mg_ha_BLUE | ILH1_2016 | 8.036 | 5.919 | 7.366 | 1.358 | 0.804 | 1.091 |
| E+G+GE+BRR | Yield_Mg_ha_BLUE | INH1_2016 | 6.533 | 1.994 | 2.069 | 3.277 | 0.964 | 3.158 |
| E+G+GE+BRR | Yield_Mg_ha_BLUE | MIH1_2016 | 4.748 | 19.667 | 20.508 | 0.241 | 0.959 | 0.232 |
| E+G+GE+BRR | Yield_Mg_ha_BLUE | MNH1_2016 | 1.422 | 1.265 | 1.248 | 1.124 | 1.014 | 1.140 |
| E+G+GE+BRR | Yield_Mg_ha_BLUE | MOH1_2016 | 12.381 | 9.392 | 11.632 | 1.318 | 0.807 | 1.064 |
| E+G+GE+BRR | Yield_Mg_ha_BLUE | NCH1_2016 | 5.713 | 3.515 | 3.888 | 1.626 | 0.904 | 1.470 |
| E+G+GE+BRR | Yield_Mg_ha_BLUE | NEH1_2016 | 5.446 | 5.214 | 4.593 | 1.045 | 1.135 | 1.186 |
| E+G+GE+BRR | Yield_Mg_ha_BLUE | NYH2_2016 | 17.271 | 19.504 | 19.128 | 0.886 | 1.020 | 0.903 |
| E+G+GE+BRR | Yield_Mg_ha_BLUE | OHH1_2016 | 2.503 | 2.138 | 2.234 | 1.171 | 0.957 | 1.121 |
| E+G+GE+BRR | Yield_Mg_ha_BLUE | WIH1_2016 | 2.210 | 3.805 | 2.586 | 0.581 | 1.471 | 0.855 |
| E+G+GE+BRR | Yield_Mg_ha_BLUE | WIH2_2016 | 4.667 | 5.288 | 5.442 | 0.883 | 0.972 | 0.858 |
| E+G+GE+BRR | Yield_Mg_ha_BLUE | Across | - | - | - | 1.117 | 0.996 | 1.095 |
| E+G+GE+BRR | Yield_Mg_ha_weight | ARH1_2016 | 2.359 | 0.719 | 1.152 | 3.281 | 0.624 | 2.047 |
| E+G+GE+BRR | Yield_Mg_ha_weight | DEH1_2016 | 0.051 | 0.020 | 0.186 | 2.540 | 0.108 | 0.273 |
| E+G+GE+BRR | Yield_Mg_ha_weight | GAH1_2016 | 0.026 | 0.387 | 0.568 | 0.068 | 0.682 | 0.046 |
| E+G+GE+BRR | Yield_Mg_ha_weight | IAH1_2016 | 2.914 | 2.808 | 2.836 | 1.038 | 0.990 | 1.027 |
| E+G+GE+BRR | Yield_Mg_ha_weight | IAH2_2016 | 0.069 | 0.110 | 0.135 | 0.626 | 0.813 | 0.509 |
| E+G+GE+BRR | Yield_Mg_ha_weight | IAH3_2016 | 0.670 | 1.666 | 3.383 | 0.402 | 0.493 | 0.198 |
| E+G+GE+BRR | Yield_Mg_ha_weight | IAH4_2016 | 0.199 | 0.058 | 0.113 | 3.423 | 0.516 | 1.766 |
| E+G+GE+BRR | Yield_Mg_ha_weight | ILH1_2016 | 0.751 | 0.594 | 0.808 | 1.264 | 0.736 | 0.930 |
| E+G+GE+BRR | Yield_Mg_ha_weight | INH1_2016 | 0.112 | 0.099 | 0.064 | 1.130 | 1.550 | 1.750 |
| E+G+GE+BRR | Yield_Mg_ha_weight | MIH1_2016 | 0.055 | 0.247 | 0.074 | 0.223 | 3.325 | 0.741 |
| E+G+GE+BRR | Yield_Mg_ha_weight | MNH1_2016 | 0.146 | 0.338 | 0.673 | 0.432 | 0.502 | 0.217 |
| E+G+GE+BRR | Yield_Mg_ha_weight | MOH1_2016 | 0.283 | 0.522 | 0.082 | 0.542 | 6.396 | 3.466 |
| E+G+GE+BRR | Yield_Mg_ha_weight | NCH1_2016 | 0.113 | 0.227 | 0.100 | 0.499 | 2.277 | 1.135 |
| E+G+GE+BRR | Yield_Mg_ha_weight | NEH1_2016 | 0.033 | 0.127 | 0.076 | 0.263 | 1.665 | 0.438 |
| E+G+GE+BRR | Yield_Mg_ha_weight | NYH2_2016 | 0.449 | 0.418 | 0.553 | 1.074 | 0.756 | 0.812 |
| E+G+GE+BRR | Yield_Mg_ha_weight | OHH1_2016 | 1.202 | 1.483 | 0.850 | 0.811 | 1.745 | 1.414 |
| E+G+GE+BRR | Yield_Mg_ha_weight | WIH1_2016 | 0.204 | 0.110 | 0.066 | 1.858 | 1.672 | 3.107 |
| E+G+GE+BRR | Yield_Mg_ha_weight | WIH2_2016 | 0.185 | 0.073 | 1.076 | 2.524 | 0.068 | 0.172 |
| E+G+GE+BRR | Yield_Mg_ha_weight | Across | - | - | - | 1.222 | 1.384 | 1.114 |

| **Table B7**. Variance components (Var_Comp) for environment (Env) Line and Genotype  by environment (Env:Line) interaction for each data set. CV denotes coefficient of variation  and n_Env denotes the average of number of environments in each data set. | | | | | | |
| --- | --- | --- | --- | --- | --- | --- |
| Data | Component | VarComp | Trait | Heritability | CV | n_Env |
| Japonica | Env:Line | 186065.908 | GY | 0.285 | 0.163 | 3.597 |
| Japonica | Line | 257287.998 | GY | 0.285 | 0.163 | 3.597 |
| Japonica | Env | 1860782.427 | GY | 0.285 | 0.163 | 3.597 |
| Japonica | Residual | 272836.420 | GY | 0.285 | 0.163 | 3.597 |
| Japonica | Env:Line | 0.000 | PHR | 0.462 | 0.073 | 3.597 |
| Japonica | Line | 0.000 | PHR | 0.462 | 0.073 | 3.597 |
| Japonica | Env | 0.001 | PHR | 0.462 | 0.073 | 3.597 |
| Japonica | Residual | 0.000 | PHR | 0.462 | 0.073 | 3.597 |
| Japonica | Env:Line | 0.000 | GC | 0.249 | 0.818 | 3.597 |
| Japonica | Line | 0.001 | GC | 0.249 | 0.818 | 3.597 |
| Japonica | Env | 0.006 | GC | 0.249 | 0.818 | 3.597 |
| Japonica | Residual | 0.001 | GC | 0.249 | 0.818 | 3.597 |
| Japonica | Env:Line | 0.002 | PH | 0.624 | 0.097 | 3.597 |
| Japonica | Line | 20.528 | PH | 0.624 | 0.097 | 3.597 |
| Japonica | Env | 35.950 | PH | 0.624 | 0.097 | 3.597 |
| Japonica | Residual | 8.576 | PH | 0.624 | 0.097 | 3.597 |
| USP | Env:Line | 0.983 | GY | 0.533 | 0.378 | 4 |
| USP | Line | 1.129 | GY | 0.533 | 0.378 | 4 |
| USP | Env | 2.123 | GY | 0.533 | 0.378 | 4 |
| USP | Residual | 0.850 | GY | 0.533 | 0.378 | 4 |
| G2F_2014 | Env:Line | 0.001 | Grain_Moisture_BLUE | 0.609 | 0.196 | 5.376 |
| G2F_2014 | Line | 3.913 | Grain_Moisture_BLUE | 0.609 | 0.196 | 5.376 |
| G2F_2014 | Env | 11.492 | Grain_Moisture_BLUE | 0.609 | 0.196 | 5.376 |
| G2F_2014 | Residual | 2.006 | Grain_Moisture_BLUE | 0.609 | 0.196 | 5.376 |
| G2F_2014 | Env:Line | 1.061 | Grain_Moisture_weight | 0.010 | 1.877 | 5.376 |
| G2F_2014 | Line | 0.344 | Grain_Moisture_weight | 0.010 | 1.877 | 5.376 |
| G2F_2014 | Env | 175.200 | Grain_Moisture_weight | 0.010 | 1.877 | 5.376 |
| G2F_2014 | Residual | 3.331 | Grain_Moisture_weight | 0.010 | 1.877 | 5.376 |
| G2F_2014 | Env:Line | 0.697 | Yield_Mg_ha_BLUE | 0.423 | 0.271 | 5.376 |
| G2F_2014 | Line | 0.822 | Yield_Mg_ha_BLUE | 0.423 | 0.271 | 5.376 |
| G2F_2014 | Env | 4.475 | Yield_Mg_ha_BLUE | 0.423 | 0.271 | 5.376 |
| G2F_2014 | Residual | 0.853 | Yield_Mg_ha_BLUE | 0.423 | 0.271 | 5.376 |
| G2F_2014 | Env:Line | 0.118 | Yield_Mg_ha_weight | 0.461 | 0.576 | 5.376 |
| G2F_2014 | Line | 0.162 | Yield_Mg_ha_weight | 0.461 | 0.576 | 5.376 |
| G2F_2014 | Env | 0.699 | Yield_Mg_ha_weight | 0.461 | 0.576 | 5.376 |
| G2F_2014 | Residual | 0.202 | Yield_Mg_ha_weight | 0.461 | 0.576 | 5.376 |
| G2F_2015 | Env:Line | 0.001 | Grain_Moisture_BLUE | 0.603 | 0.160 | 4.217 |
| G2F_2015 | Line | 2.004 | Grain_Moisture_BLUE | 0.603 | 0.160 | 4.217 |
| G2F_2015 | Env | 3.286 | Grain_Moisture_BLUE | 0.603 | 0.160 | 4.217 |
| G2F_2015 | Residual | 2.270 | Grain_Moisture_BLUE | 0.603 | 0.160 | 4.217 |
| G2F_2015 | Env:Line | 0.001 | Grain_Moisture_weight | 0.109 | 1.435 | 4.217 |
| G2F_2015 | Line | 0.655 | Grain_Moisture_weight | 0.109 | 1.435 | 4.217 |
| G2F_2015 | Env | 19.808 | Grain_Moisture_weight | 0.109 | 1.435 | 4.217 |
| G2F_2015 | Residual | 2.699 | Grain_Moisture_weight | 0.109 | 1.435 | 4.217 |
| G2F_2015 | Env:Line | 1.002 | Yield_Mg_ha_BLUE | 0.359 | 0.272 | 4.217 |
| G2F_2015 | Line | 0.633 | Yield_Mg_ha_BLUE | 0.359 | 0.272 | 4.217 |
| G2F_2015 | Env | 2.604 | Yield_Mg_ha_BLUE | 0.359 | 0.272 | 4.217 |
| G2F_2015 | Residual | 1.164 | Yield_Mg_ha_BLUE | 0.359 | 0.272 | 4.217 |
| G2F_2015 | Env:Line | 0.007 | Yield_Mg_ha_weight | 0.361 | 0.660 | 4.217 |
| G2F_2015 | Line | 0.048 | Yield_Mg_ha_weight | 0.361 | 0.660 | 4.217 |
| G2F_2015 | Env | 0.284 | Yield_Mg_ha_weight | 0.361 | 0.660 | 4.217 |
| G2F_2015 | Residual | 0.070 | Yield_Mg_ha_weight | 0.361 | 0.660 | 4.217 |
| G2F_2016 | Env:Line | 0.000 | Grain_Moisture_BLUE | 0.830 | 0.142 | 10.055 |
| G2F_2016 | Line | 2.387 | Grain_Moisture_BLUE | 0.830 | 0.142 | 10.055 |
| G2F_2016 | Env | 3.584 | Grain_Moisture_BLUE | 0.830 | 0.142 | 10.055 |
| G2F_2016 | Residual | 1.335 | Grain_Moisture_BLUE | 0.830 | 0.142 | 10.055 |
| G2F_2016 | Env:Line | 0.014 | Grain_Moisture_weight | 0.109 | 1.259 | 10.055 |
| G2F_2016 | Line | 0.468 | Grain_Moisture_weight | 0.109 | 1.259 | 10.055 |
| G2F_2016 | Env | 34.317 | Grain_Moisture_weight | 0.109 | 1.259 | 10.055 |
| G2F_2016 | Residual | 4.322 | Grain_Moisture_weight | 0.109 | 1.259 | 10.055 |
| G2F_2016 | Env:Line | 1.477 | Yield_Mg_ha_BLUE | 0.736 | 0.252 | 10.055 |
| G2F_2016 | Line | 1.337 | Yield_Mg_ha_BLUE | 0.736 | 0.252 | 10.055 |
| G2F_2016 | Env | 2.211 | Yield_Mg_ha_BLUE | 0.736 | 0.252 | 10.055 |
| G2F_2016 | Residual | 1.133 | Yield_Mg_ha_BLUE | 0.736 | 0.252 | 10.055 |
| G2F_2016 | Env:Line | 0.020 | Yield_Mg_ha_weight | 0.341 | 0.598 | 10.055 |
| G2F_2016 | Line | 0.023 | Yield_Mg_ha_weight | 0.341 | 0.598 | 10.055 |
| G2F_2016 | Env | 0.372 | Yield_Mg_ha_weight | 0.341 | 0.598 | 10.055 |
| G2F_2016 | Residual | 0.051 | Yield_Mg_ha_weight | 0.341 | 0.598 | 10.055 |
